# Preparation and Evaluation of Hepatoma-Targeting Glycyrrhetinic Acid Composite Micelles Loaded with Curcumin

**DOI:** 10.3390/ph18040448

**Published:** 2025-03-23

**Authors:** Xueli Guo, Zhongyan Liu, Lina Wu, Pan Guo

**Affiliations:** 1 State Key Laboratory of Component-Based Chinese Medicine, Tianjin University of Traditional Chinese Medicine, Tianjin 301617, China; 15516164560@163.com (X.G.); lzy1337518827@163.com (Z.L.); wulina13108@163.com (L.W.); 2 Ministry of Education, Tianjin University of Traditional Chinese Medicine, Tianjin 301617, China

**Keywords:** glycyrrhizic acid, glycyrrhetinic acid, liver cancer, target delivery, micelles

## Abstract

**Background**: Liver cancer, especially hepatocellular carcinoma, a prevalent malignant tumor of the digestive system, poses significant therapeutic challenges. While traditional chemotherapy can inhibit tumor progression, its clinical application is limited by insufficient efficacy. Hydrophobic therapeutic agents further encounter challenges including low tumor specificity, poor bioavailability, and severe systemic toxicity. This study aimed to develop a liver-targeted, glutathione (GSH)-responsive micellar system to synergistically enhance drug delivery and antitumor efficacy. **Methods**: A GSH-responsive disulfide bond was chemically synthesized to conjugate glycyrrhetinic acid (GA) with curcumin (Cur) at a molar ratio of 1:1, forming a prodrug Cur-GA (CGA). This prodrug was co-assembled with glycyrrhizic acid (GL) at a 300% *w*/*w* loading ratio into micelles. The system was characterized for physicochemical properties, in vitro drug release in PBS (7.4) without GSH and in PBS (5.0) with 0, 5, or 10 mM GSH, cellular uptake in HepG2 cells, and in vivo efficacy in H22 hepatoma-bearing BALB/c mice. **Results**: The optimized micelles exhibited a hydrodynamic diameter of 157.67 ± 2.14 nm (PDI: 0.20 ± 0.02) and spherical morphology under TEM. The concentration of CUR in micelles can reach 1.04 mg/mL. In vitro release profiles confirmed GSH-dependent drug release, with 67.5% vs. <40% cumulative Cur release observed at 24 h with/without 10 mM GSH. Flow cytometry and high-content imaging revealed 1.8-fold higher cellular uptake of CGA-GL micelles compared to free drug (*p* < 0.001). In vivo, CGA-GL micelles achieving 3.6-fold higher tumor accumulation than non-targeted controls (*p* < 0.001), leading to 58.7% tumor volume reduction (*p* < 0.001). **Conclusions**: The GA/GL-based micellar system synergistically enhanced efficacy through active targeting and stimuli-responsive release, providing a promising approach to overcome current limitations in hydrophobic drug delivery for hepatocellular carcinoma therapy.

## 1. Introduction

Malignant tumors, characterized by aberrant cellular differentiation, uncontrolled proliferation, and metastatic potential, represent a major threat to global health [1,2,3]. In China, hepatocellular carcinoma (HCC) accounts for over 50% of global cases, with rising incidence and mortality rates [4,5,6]. Hepatocellular carcinoma (HCC) is currently one of the most common malignant liver cancers, with more than half of new cases globally reported in China, severely endangering the lives and health of the Chinese population [7,8]. Despite advances in surgical and radiological interventions, approximately 70% of HCC patients are diagnosed at advanced stages, where systemic therapies remain the primary option [9,10]. While surgery and radiotherapy are limited to local tumor targeting, drug therapy is more suitable for tumors with systemic dissemination, especially in patients with advanced-stage tumors or metastases [11,12,13]. However, conventional chemotherapeutic agents face critical limitations, including poor aqueous solubility, non-specific biodistribution, and dose-limiting toxicity [14,15]. These issues considerably limit the clinical application of therapeutic drugs. Thus, developing drugs with high antitumor efficacy, strong in vivo targeting, high bioavailability, and minimal systemic toxicity is crucial.

Micellar systems, as nanocarriers for drugs, exhibit unique advantages in addressing numerous challenges in cancer treatment, yet limitations in current designs necessitate further innovation. Their design is based on the following core mechanisms. First, the amphiphilic nature of micelles allows them to efficiently encapsulate hydrophobic anticancer drugs (such as paclitaxel and doxorubicin) through their hydrophobic core [16,17]. This significantly enhances the water solubility and in vivo stability of these drugs, overcoming the limitations of traditional formulations, which often have low bioavailability and high toxicity. Then, the nanoscale size of micelles (20–200 nm) enables them to exploit the enhanced permeability and retention (EPR) effect in tumor tissues [18]. This results in selective accumulation at tumor sites, achieving passive targeting. Studies have shown that the EPR effect can increase drug concentrations in tumor tissues compared to normal tissues [19,20]. In addition, by incorporating responsive moieties (such as pH-sensitive ketals [21], reduction-sensitive disulfide bonds [22], or enzyme-sensitive peptides [23]), micelles can trigger precise drug release in the tumor microenvironment (characterized by acidity, high glutathione concentrations, or specific enzymes) [24]. This effectively reduces systemic toxicity while enhancing therapeutic efficacy. Despite these advances, current micellar systems face some unresolved challenges: the low stability of micelle structure; overreliance on passive EPR targeting; and limited biocompatibility of synthetic stabilizers (e.g., PEG and pluronics). These gaps highlight the need for more efficient and natural micellar systems [25,26].

Traditional Chinese medicine (TCM) has emerged as a valuable resource for tumor and targeting therapeutics [27]. Curcumin (Cur), a natural polyphenol derived from *Curcuma longa*, exhibits potent antitumor activity via multiple mechanisms, including anti-angiogenesis, cell cycle arrest, and the inhibition of NF-κB signaling [28,29]. However, its clinical translation is hindered by extremely low aqueous solubility (<1 μg/mL), rapid systemic metabolism, and poor tumor accumulation [30]. To address these limitations, the encapsulation of Cur into micellar systems has emerged as a promising strategy. For instance, studies have demonstrated that micelles can enhance Cur solubility by 2500-fold and prolong its circulation half-life [31], yet existing carriers (e.g., PLGA nanoparticles [32] and liposomes [32]) lack liver-specific targeting and microenvironment-responsive release, limiting their efficacy in HCC.

Glycyrrhizic acid (GL), a triterpenoid saponin from *Glycyrrhiza* species, consists of a hydrophilic glucuronide moiety and a hydrophobic aglycone (glycyrrhetinic acid, GA) [33]. GL functions as a natural surfactant with amphiphilic properties, demonstrating good biocompatibility and safety, and has been approved by the European Union as a food additive (E958) and pharmaceutical excipient [34,35]. GL and GA bind to receptors highly expressed on hepatocyte membranes, facilitating liver-targeted drug delivery [36]. Notably, GA exhibits higher affinity than GL for hepatocyte membrane receptors (e.g., protein kinase C-α, PKCα), enabling selective liver targeting [37]. Mechanistically, GA binds to PKCα, which is overexpressed in HCC cells compared to normal hepatocytes (1.5- to 5-fold, *p* < 0.01) [38]. Structural-activity studies further reveal that the C18 β-hydrogen configuration is critical for receptor binding, while C3/C11 modifications minimally affect targeting [39]. These properties have spurred the development of GA-based nanocarriers that have demonstrated 4.7-fold higher tumor accumulation compared to non-targeted counterparts in HCC models [40].

Apparently, Cur and GA/GL exhibit synergistic antitumor effects. While Cur suppresses angiogenesis and metastasis via VEGF and MMP-9 inhibition [41], GA directly induces apoptosis in HCC cells through PKCα-mediated caspase activation [42]. This combination may overcome compensatory survival pathways triggered by single-target therapies [43]. Despite progress in GL/GA-based carriers [44], such as liposomes [45], two critical challenges remain: (1) low drug loading capacity for hydrophobic agents like Cur due to insufficient core hydrophobicity and (2) a lack of microenvironment-responsive release to minimize off-target toxicity. 

Furthermore, recent advances in stimuli-responsive drug delivery systems exploit tumor microenvironment (TME) features such as redox gradients [46]. The intracellular glutathione (GSH) concentration in tumors (3–10 mM) is 100–1000-fold higher than extracellular levels (2–20 μM) [47,48], creating a reductive milieu conducive to disulfide bond cleavage, providing a robust trigger for TME-specific drug release. Additionally, most existing GA carriers rely solely on passive EPR targeting, neglecting active receptor-mediated uptake and TME-triggered release.

Herein, we report a GSH-activatable, liver-targeted micellar system co-delivering Cur, GA, and GL. The prodrug CUR-GA (CGA) was synthesized via a disulfide linkage of Cur to GA, then combined with GL to form nanocomposite micelles. Key innovations include the following:

1. Dual targeting via GA receptor-mediated active uptake and EPR-driven passive accumulation, achieving higher HCC selectivity than non-targeted micelles.
2. Synergistic antitumor effects from Cur and GA/GL.
3. Enhanced biocompatibility through using natural surfactant stabilization of GL, avoiding synthetic polymers that induce accelerated blood clearance, while enabling GSH-triggered drug release.

## 2. Results

### 2.1. Characterization of CGA Polymer

#### 2.1.1. FTIR Analysis

**Figure 1:**
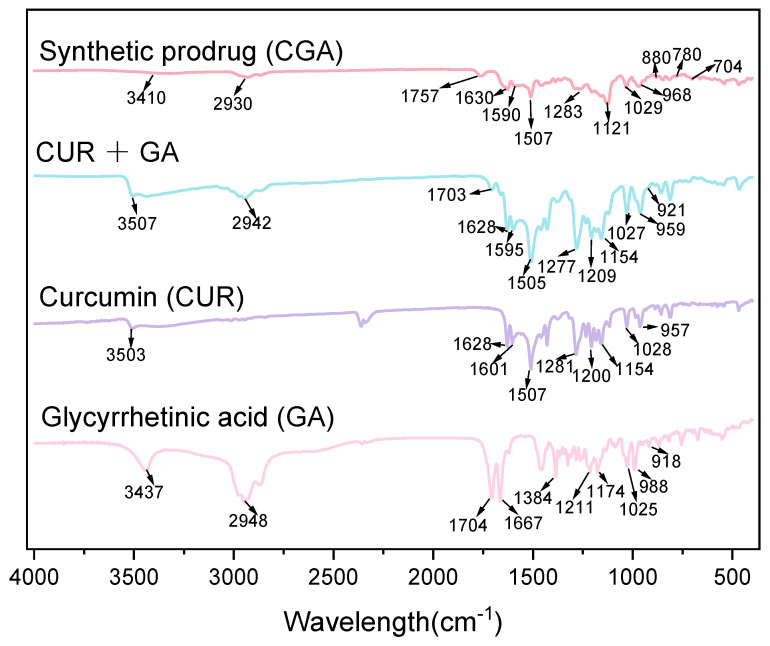
FTIR spectra of CUR, GA, their physical mixture (CUR + GA), and the synthesized compound CGA.

The infrared spectra of CGA, GA, CUR, and GA + CUR are shown in Figure 1. In CUR, the broad peak at 3550–3300 cm⁻^1^ corresponds to the alcoholic or phenolic hydroxyl group. Peaks at 1601 cm⁻^1^ and 1507 cm⁻^1^ are attributed to ν(C=C) vibrations of the aromatic ring, the peak at 1281 cm⁻^1^ corresponds to the ν(Ar-O) stretching vibration of the phenolic hydroxyl group, and the peak at 1028 cm⁻^1^ is assigned to the ν(C-O) stretching vibration of the methoxy group (-OCH_3_) on the benzene ring. The peak at 957 cm⁻^1^ corresponds to the C-H out-of-plane bending vibration (β(C=C-H)) of the trans-configured double bond in the aliphatic chain. This assignment is consistent with the typical range for trans C=C-H bending (960–980 cm⁻^1^) and literature data on CUR. The peak at 1628 cm⁻^1^ is associated with the conjugation of α, β-unsaturated ketones in CUR, which shifts the ν(C=O) absorption peak to lower wavenumbers [49]. For GA, the broad O-H stretching vibration of hydrogen-bonded hydroxyl groups is observed around 3437 cm⁻^1^ (peak at 3550–3300 cm⁻^1^). The carboxylic acid absorption peaks include ν(C=O) (1740–1680 cm⁻^1^), ν(O-H) (3050–2800 cm⁻^1^), and γ(O-H) (950–918 cm⁻^1^). The conjugation of α, β-unsaturated ketones in GA shifts the strong ν(C=O) peak from 1715 cm⁻^1^ to 1704 cm⁻^1^. The saturated C-H stretching peak of GA is observed at 2948 cm⁻^1^ [50].

In the FTIR of the physical mixture of CUR and GA, the characteristic peaks of CUR and GA remain present, particularly the C=O stretching vibration peak of the free carboxylic acid at 1703 cm⁻^1^, indicating that no chemical bonding (e.g., esterification or covalent linkage) has formed between the two compounds. In CGA, peaks at 1590 cm⁻^1^ and 1507 cm⁻^1^ correspond to the ν(C=C) of the benzene ring, while peaks at 704 cm⁻^1^, 780 cm⁻^1^, and 880 cm⁻^1^ are assigned to γ(Ar-H). Peaks at 1680–1630 cm⁻^1^, 3500–3100 cm⁻^1^, and 1540–1500 cm⁻^1^ represent ν(C=O), ν(N-H), and β(N-H) vibrations of amides. The broad band at 1300–1000 cm⁻^1^ confirms the presence of hydroxyl groups. The γ(OH) peak at 949–917 cm⁻^1^ in GA/CUR + GA is absent in CGA, suggesting that GA’s carboxyl group is involved in amide bond formation. This analysis confirms the successful synthesis of CGA.

#### 2.1.2. 1H-NMR Analysis

**Figure 2:**
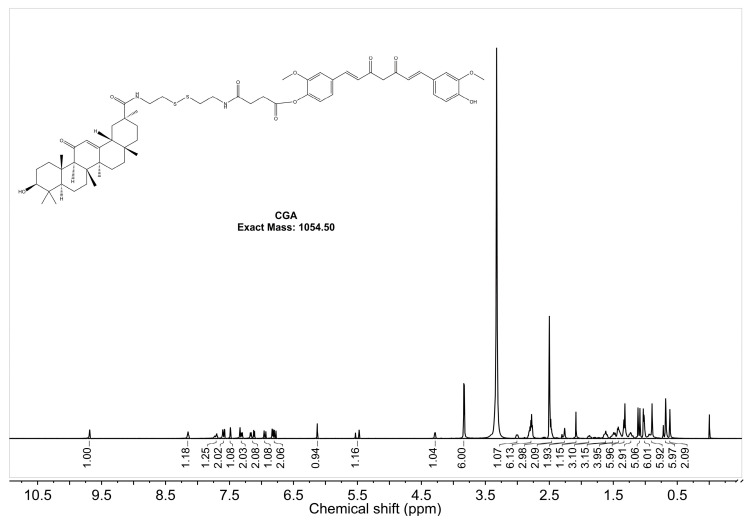
^1^H-NMR (600 MHz, DMSO-d_6_) spectrum of the synthesized compound CGA.

The ^1^H-NMR spectrum of CGA in DMSO-d_6_ with a resonance frequency of 600 MHz is shown in Figure 2. The single peak at 9.69 ppm corresponds to the OH group in CUR, indicating a shift from two peaks to one [51]. The absence of a peak at 12.18 ppm suggests that GA’s -COOH group has reacted [52]. The aromatic hydrocarbon region (6.5–8.0 ppm, 16H) corresponds to CUR (13H), amides (2H), and GA (1H). The methoxy peak of CUR (3.8457 ppm, 6H) splits into two peaks (3.8397 ppm, 3H; 3.8317 ppm, 3H), reflecting a change in the chemical environment. The double bond peak of GA remains visible, confirming that GA retains its structural integrity. These results verify the successful synthesis of CGA. Peak assignments for CUR, GA, and CGA are detailed in Table A1.

#### 2.1.3. HPLC-MS Analysis

**Figure 3:**
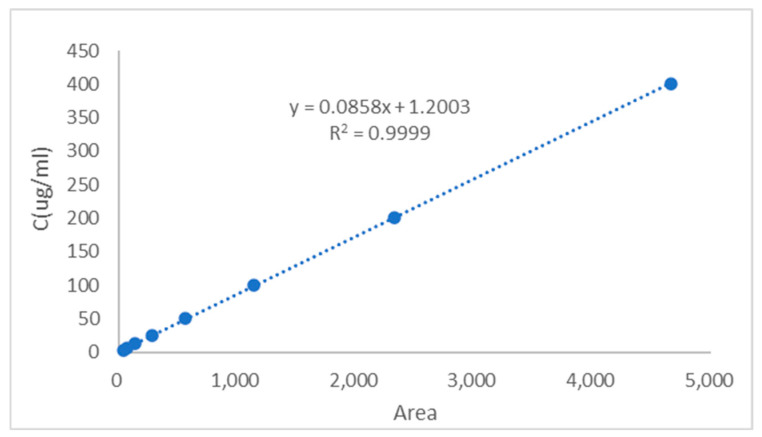
HPLC-UV calibration curve of CGA in methanol. The calibration equation *y* = 0.0858*x* + 1.2003 with *R*^2^ = 0.9999 indicates excellent linearity.

**Figure 4:**
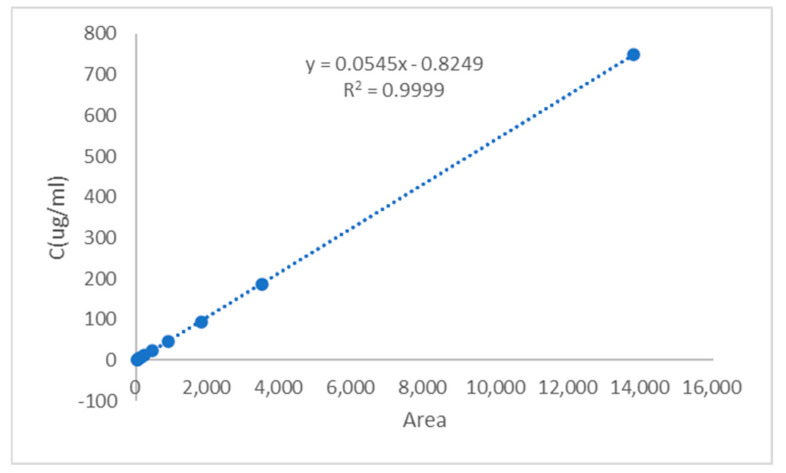
HPLC-UV calibration curve of CUR in methanol. The calibration equation *y* = 0.0545*x* − 0.8249 with *R*^2^ = 0.9999 indicates excellent linearity.

The regression equation was established by plotting the CGA/CUR solution concentration *y* (μg/mL) as the *y*-axis and peak area *x* as the *x*-axis (Figure 3 and Figure 4). The calibration curve equation of CGA was *y* = 0.0858*x* + 1.2003, with a correlation coefficient of *R*^2^ = 0.9999, indicating excellent linearity for CGA within the concentration range of 3.13–405.00 μg/mL. The calibration curve equation of CUR was *y* = 0.0545*x* − 0.8249, with a correlation coefficient of *R*^2^ = 0.9999, demonstrating good linearity for CUR across the range of 1.46–749.55 μg/mL.

The HPLC method was validated for sensitivity, linearity, and precision. The limit of detection (LOD) and limit of quantification (LOQ) were calculated based on the standard deviation of the blank (*n* = 3) and the slope of the calibration curve, using the following equations:

LOD = 3.3σ/S (1)

LOQ = 10σ/S (2)

where σ is the standard deviation of the blank signal, and S is the slope.

The LOD and LOQ for CGA were determined to be 0.015 μg/mL and 0.046 μg/mL, respectively. The LOD and LOQ for CUR were determined to be 0.024 μg/mL and 0.073 μg/mL, respectively, indicating high sensitivity for trace analysis. These results are presented in the Table 1.

The precision results are summarized in Table 1. Within the linear ranges, the relative standard deviations (RSDs) of intra-day and inter-day precision for both CUR and CGA were below 3%, confirming satisfactory instrumental precision. The method proved suitable for quantifying CUR and CGA with high accuracy and reliability.

The precision of the analytical method was assessed by analyzing three replicates (*n* = 3) of CGA and CUR at low, medium, and high concentrations on the same day (intra-day) and over three consecutive days (inter-day). As shown in Table 2, both compounds exhibited excellent precision with RSD values consistently below 2%; these results indicate that the method is sufficiently precise for the routine analysis of both compounds. Furthermore, the 24 h stability study (Table 3) revealed that both CGA and CUR solutions maintained consistent concentrations across all tested levels with minor fluctuations observed at 0-24 h. The stability demonstrated less than 3% variation from initial concentrations, confirming the compounds’ structural integrity under experimental conditions over the 24 h period.

**Figure 5:**
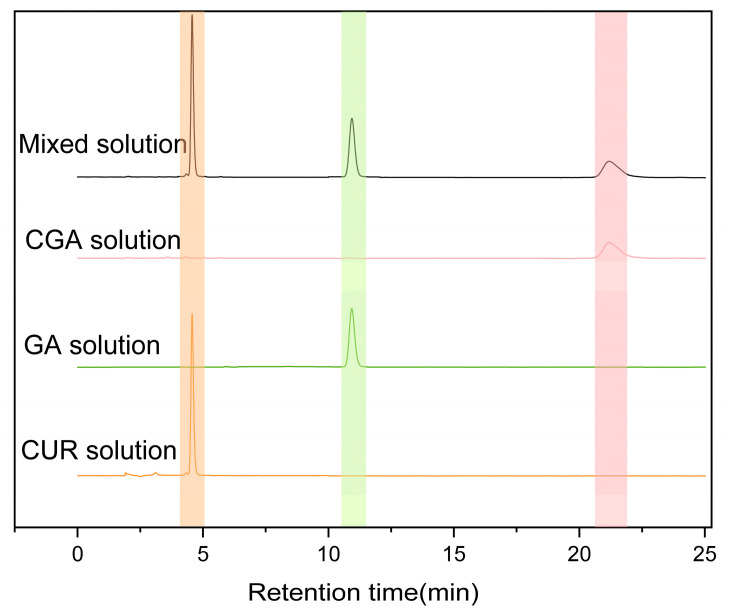
HPLC chromatograms of CUR, GA, CGA, and their physical mixture (mixed solution). CUR solution: main peak at 4.9 min (Orange mark); GA solution: main peak at 10.4 min (Green mark); CGA solution: main peak at 21.0 min (Pink mark); and mixed solution: peaks at 20.9 min (CGA) and peaks at 4.9 min (CUR) and 10.5 min (GA) retained. Data processing: peaks integrated with auto-threshold (*n* = 3, RSD% < 3%).

As shown in Figure 5, under the established chromatographic conditions, CUR, CGA, and GA exhibited good resolution with no interference in content determination, indicating excellent specificity of CGA under these conditions. The retention times for CUR and CGA at 254 nm were approximately 4.9 min and 21 min, respectively.

**Figure 6:**
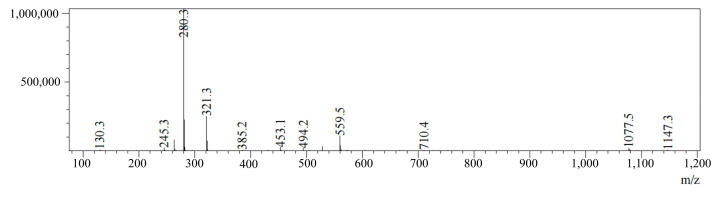
HPLC-MS analysis of CGA.

The HPLC-MS analysis of CGA reveals a retention time of 2.275 min and an [M + Na]⁺ signal at *m*/*z* = 1077.5, as shown in Figure 6. This is consistent with CGA’s theoretical molecular mass, confirming successful synthesis.

### 2.2. Characterization of Drug-Loaded Micelles

**Figure 7:**
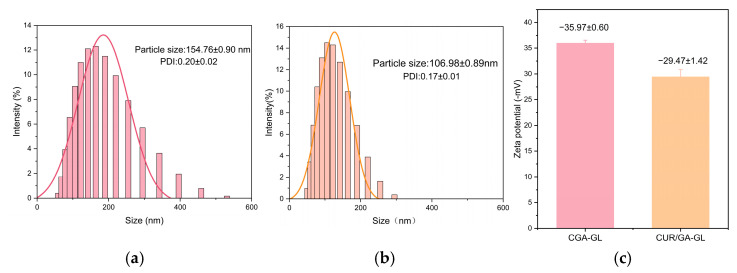
Characterization of micelle size, polydispersity index (PDI), and zeta potential. (**a**) Particle size distribution (154.76 ± 0.90 nm) and PDI (0.20 ± 0.02) of CGA-GL micelles; (**b**) particle size distribution (106.98 ± 0.89 nm) and PDI (0.17 ± 0.01) of CUR/GA-GL micelles; and (**c**) comparative zeta potential of CGA-GL micelles (−35.97 ± 0.60 mV) and CUR/GA-GL micelles (−29.47 ± 1.42 mV). Micelles dispersed in deionized water (25 °C). Data expressed as mean ± SD (*n* = 3).

**Figure 8:**
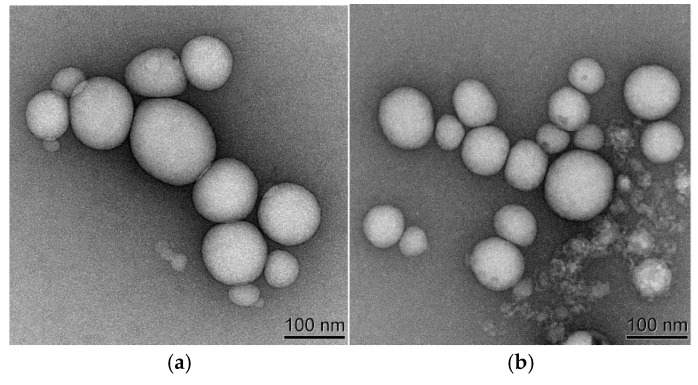
Transmission electron microscopy (TEM) images of micelles: (**a**) CGA-GL micelles and (**b**) CUR/GA-GL micelles. (Scale bar: 100 nm).

**Figure 9:**
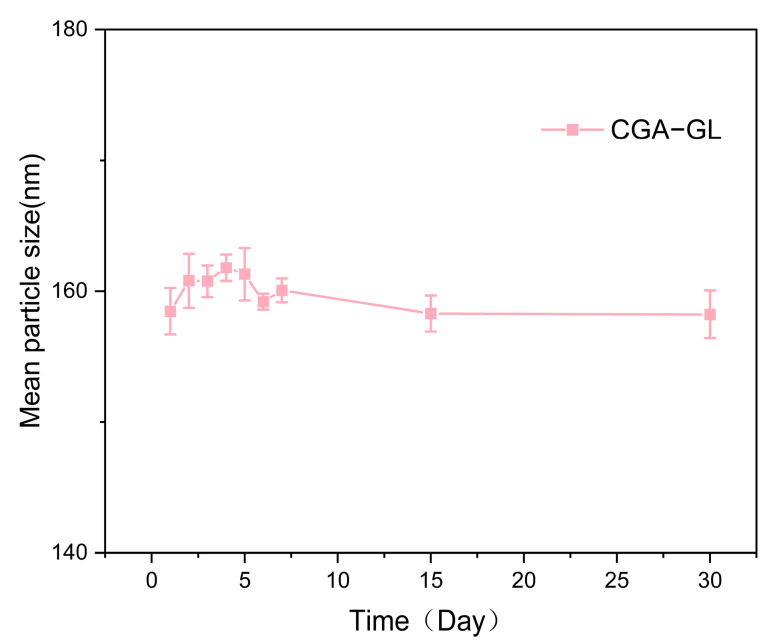
Stability of CGA-GL micelles: particles size changes over 30 days. (Micelles in deionized water at 4 °C protected from light, *n* = 3).

Table 4 and Figure 7a–c summarized the particle size, PDI, and potential of CGA-GL and CUR/GA-GL micelles. The particle size of CUR/GA-GL micelles is approximately 106.98 nm, while that of CGA-GL micelles increases slightly to 154.76 nm due to chemical modification. The zeta potential of CGA-GL micelles becomes less negative compared to CUR/GA-GL micelles but remains negative overall. TEM images in Figure 8a,b, Figures S1 and S2 show that both CUR/GA-GL and CGA-GL micelles are spherical, uniform in size, and well-dispersed without aggregation. Figure 9 demonstrates the stability of CGA-GL micelles, with particle size remaining consistent over time.

### 2.3. Drug Loading and Encapsulation Efficiency of Micelles

**Figure 10:**
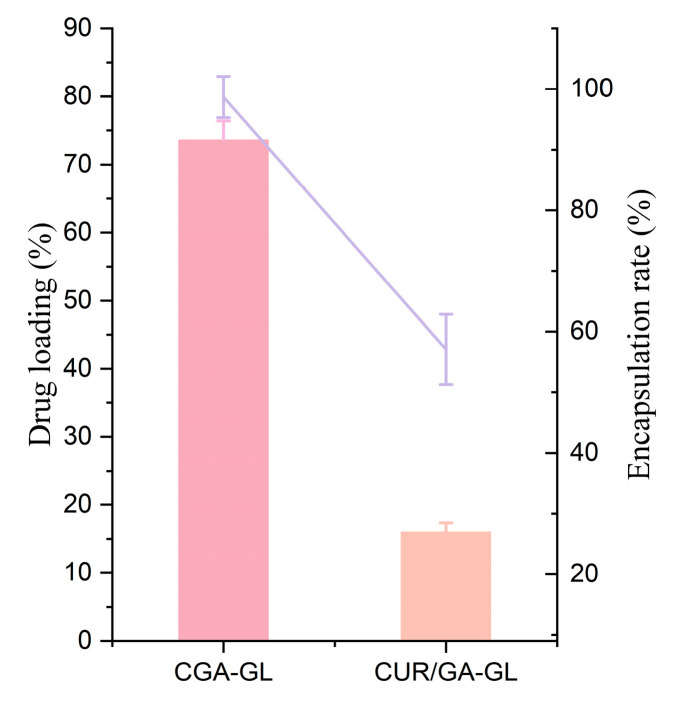
Drug loading (histogram) and encapsulation efficiency (line chart) of CGA-GL micelles and CUR/GA-GL micelles.

As shown in Table 4 and Figure 10, CGA-GL micelles exhibit higher drug loading and encapsulation efficiency than CUR/GA-GL micelles. The concentration of CGA in CGA-GL micelles can reach 2.97 mg/mL, and the encapsulation efficiency of CUR can reach 25.93%. The chemical synthesis of CGA improves encapsulation performance compared to the physical mixing of CUR and GA.

### 2.4. In Vitro Drug Release

**Figure 11:**
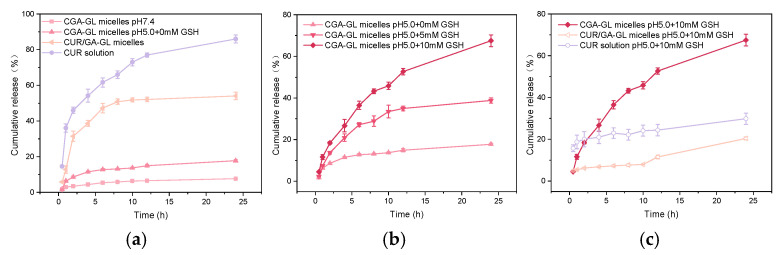
In vitro drug release profiles of micelles under different conditions. (**a**) pH-dependent release: CUR solution, CUR/GA-GL micelles, and CGA-GL micelles at pH 7.4 vs. CGA-GL micelles at pH 5.0. (**b**) Redox-triggered release: CGA-GL micelles with 0, 5, or 10 mM glutathione (GSH) at pH 5.0. (**c**) Simulating the release conditions of tumor microenvironment (TME): CUR solution, CUR/GA-GL micelles, and CGA-GL micelles with 10 mM GSH at pH 5.0. (*n* = 3).

The in vitro release results are shown in Figure 11. The CUR solution group had a cumulative release rate of (85.97 ± 2.19) % in pH 7.4 buffer solution after 24 h, which was ultimately released. The CUR/GA-GL micelles achieve a cumulative release rate of 50.83 ± 1.52% at 8 h, demonstrating sustained release properties and effectively delaying CUR release. At pH 7.4 without GSH, the cumulative release of CGA-GL micelles after 24 h is merely 7.64 ± 0.28%, increasing to 17.73 ± 0.56% at pH 5.0. However, in simulated tumor microenvironment conditions (pH 5.0 with 10 mM GSH), the cumulative release reaches 67.5 ± 2.81%, significantly higher than the 38.81 ± 1.28% observed in 5 mM GSH. In contrast, the cumulative release of CUR/GA-GL micelles and CUR solution only reach 29.86% and 20.41% under a high GSH concentration in pH 5.0. Notably, CGA-GL micelles exhibit a pH- and GSH-responsive release behavior. These results demonstrate the GSH-responsive release caused by disulfide bonds, further demonstrating the advantages of CGA-GL micelles.

### 2.5. Results of CMC

**Figure 12:**
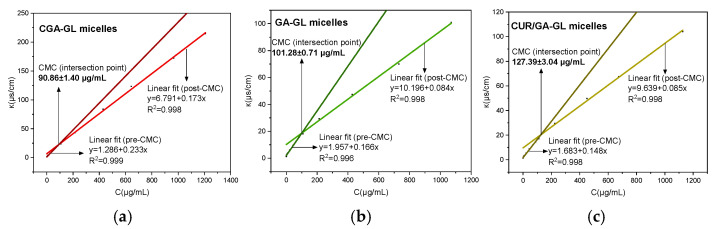
Critical micelle concentration (CMC) determination by conductivity measurements. (**a**) CGA-GL micelles, (**b**) GA-GL micelles, and (**c**) CUR/GA-GL micelles. (The experiment was conducted in parallel three times, and one of them was selected for plotting, mean ± SD, *n* = 3, RSD < 3%).

The critical micelle concentrations (CMCs) of CGA-GL, GA-GL, and CUR/GA-GL micelles were determined via the conductivity method [53]. Figure 12 illustrates the characteristic sigmoidal relationship between conductivity (κ) and the concentration (C). The CMC values were identified as the intersection point of the two linear regimes in the κ vs. C plots, corresponding to the transition from monomeric to micellar states [54]. The CGA-GL micellar system exhibited the lowest CMC (90.86 ± 1.40 μg/mL), indicating superior micellization capability compared to the GA-GL micellar system (101.28 ± 0.71 μg/mL) and the CUR/GA-GL micellar system (127.39 ± 3.04 μg/mL) [55]. This can be attributed to the covalent conjugation of CUR to GA via a disulfide bond, which enhances the hydrophobicity of the core-forming segment and optimizes the amphiphilic balance. In contrast, the physical encapsulation of CUR in the CUR/GA-GL micellar system may disrupt the hydrophilic–lipophilic equilibrium, leading to a higher CMC. Low CMC micelles are less prone to dissociation in diluted environments, which is crucial for applications such as drug delivery [56]. The disulfide linkage not only reinforces intermolecular interactions but also provides potential redox-responsive drug release, which merits further investigation.

### 2.6. Cellular Uptake

**Figure 13:**
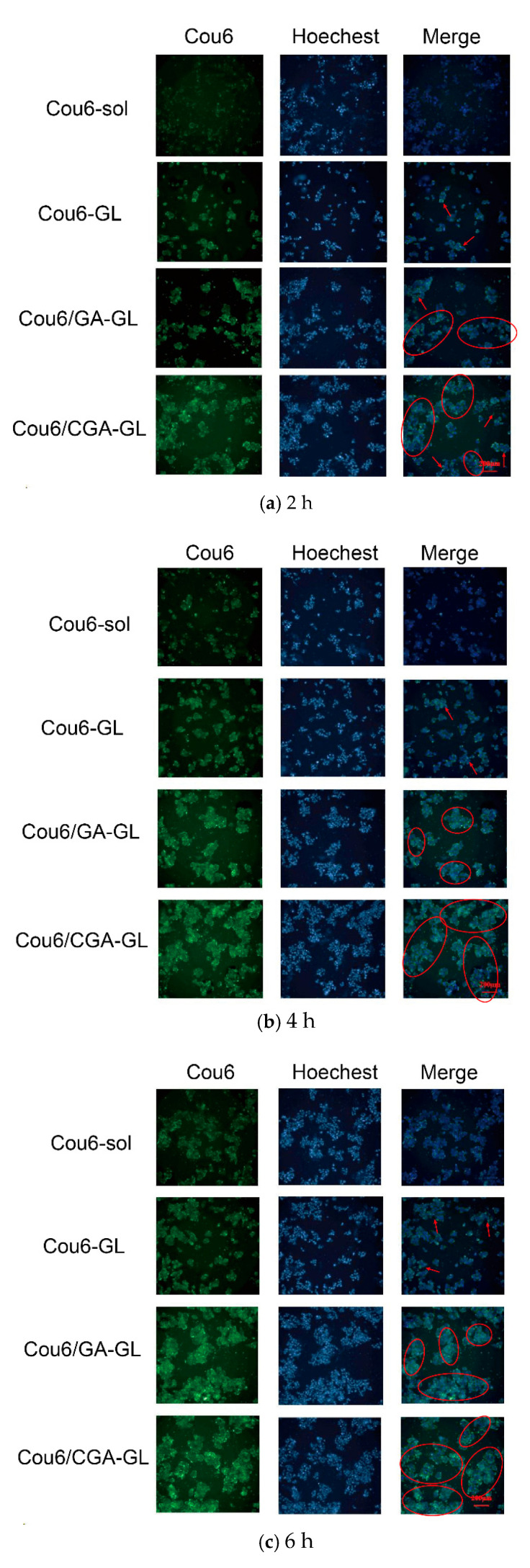
The intracellular fluorescence imaging of Cou6-loaded formulations in HepG2 cells after (**a**) 2 h, (**b**) 4 h, and (**c**) 6 h incubation. (Arrows: small scale cellular uptake; circles: Large scale cellular uptake; scale bar = 200 μm, *n* = 5).

**Figure 14:**
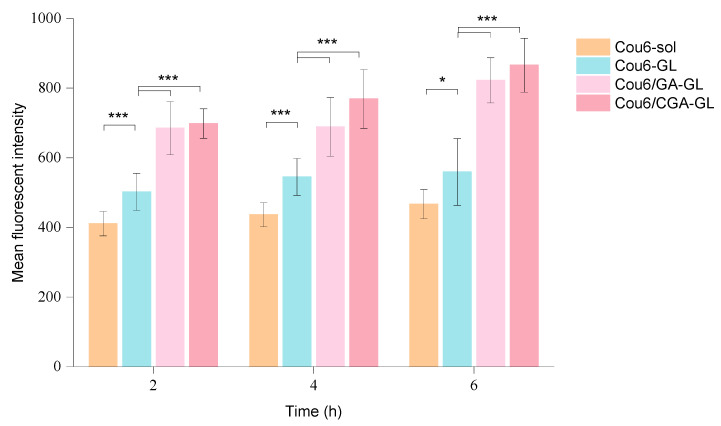
Quantitative analysis of cellular uptake efficiency by flow cytometry. Mean fluorescence intensity (MFI) of HepG2 cells treated with Cou6-solution, Cou6-GL micelles, Cou6/GA-GL micelles, and Cou6/CGA-GL micelles at 2, 4, and 6 h (*n* = 5). (* *p* < 0.05 and *** *p* < 0.001).

The intracellular fluorescence measurement results are presented in Figure 13, Figures S3 and S14, while quantitative fluorescence analysis results are shown in Figure 14. At all time points, the uptake of Cou6 solution by HepG2 cells is significantly lower than that of the three micelle groups. The fluorescence intensity of the Cou6 solution group remains stable over time, whereas the micelle groups exhibit a time-dependent increase in fluorescence intensity, consistent with the enhanced cellular uptake of other nanoparticles [57].

Among the micelle groups, Cou6/CGA-GL shows the highest fluorescence intensity, followed by Cou6/GA-GL and Cou6-GL. Notably, the two GA-containing micelle groups demonstrate significantly higher fluorescence intensity compared to the Cou6-GL group without GA (*p* < 0.001). This difference arises from distinct mechanisms of cellular uptake. Cou6 solution enters cells via passive diffusion, reaching equilibrium within 2 h, with no substantial increase in fluorescence intensity over time. Conversely, the three micelle groups primarily enter cells through endocytosis, resulting in significantly higher fluorescence intensity compared to the solution group and a gradual increase over time [58]. The presence of GA in micelle formulations enhances cellular uptake by enabling active targeting through the specific recognition of GA by tumor cell surface receptors [59]. These findings indicate that Cou6/CGA-GL and Cou6/GA-GL micelles improve cellular uptake and targeting efficacy via receptor-mediated endocytosis.

**Figure 15:**
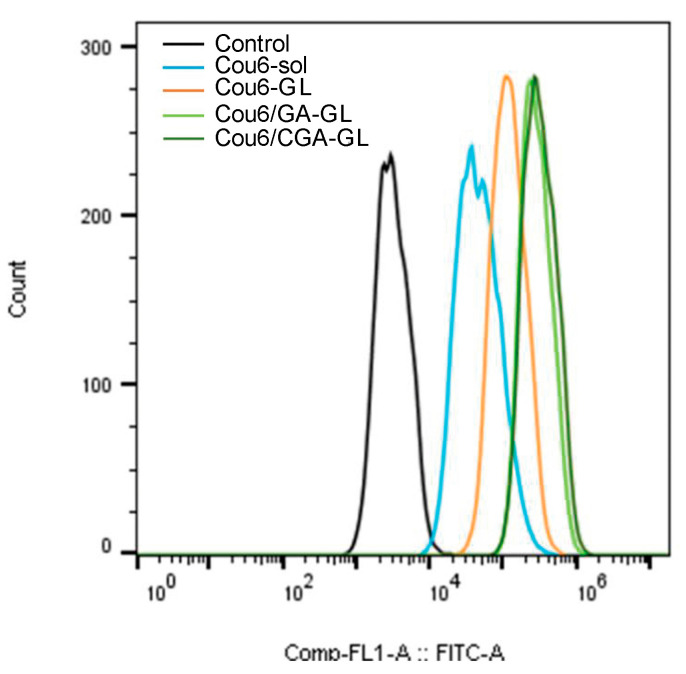
Flow cytometry histograms of cellular uptake efficiency in HepG2 cells.

Flow cytometry further quantifies intracellular fluorescence and corroborates the high-content imaging results. The uptake of all micelle preparations by HepG2 cells is measurably higher than in the Cou6 solution group, with fluorescence intensity increasing over time. Among the micelle groups, Cou6/CGA-GL achieves the highest fluorescence intensity, followed by Cou6/GA-GL and Cou6-GL. Figure 15 illustrates fluorescence intensity after 6 h of incubation for each group. Together, the results from flow cytometry and high-content imaging demonstrate that Cou6/CGA-GL and Cou6/GA-GL micelles significantly enhance intracellular accumulation in tumor cells. These findings confirm that GA-containing micelles promote drug uptake via receptor-mediated endocytosis, enhancing active targeting efficiency.

### 2.7. Cell Cytotoxicity

**Figure 16:**
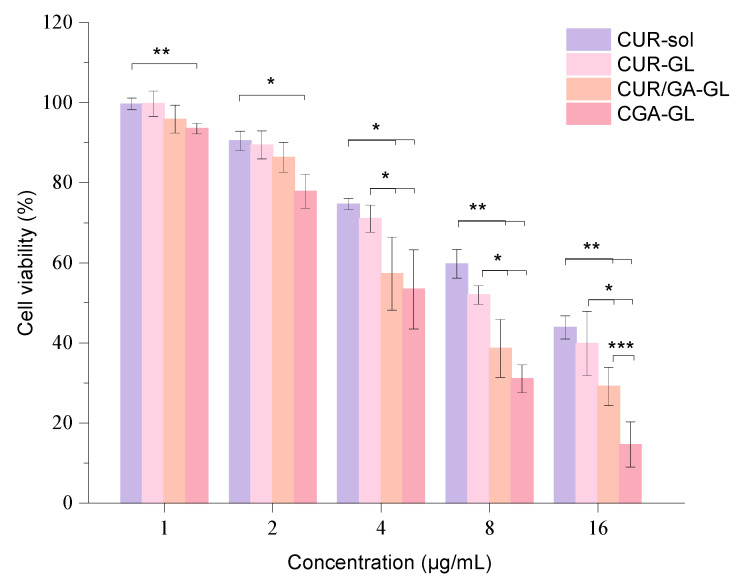
Concentration-dependent cytotoxicity of CUR formulations in HepG2 cells. (*R*^2^ > 0.95, *n* = 5, * *p* < 0.05, ** *p* < 0.01, and *** *p* < 0.001).

The inhibition rate results of CUR sol and CGA-GL on HepG2 cells show clear concentration dependence, with the inhibition rate increasing as drug concentration rises. After logarithmic transformation and linear fitting, the IC50 values for CUR sol and CGA-GL were calculated to be 14.16 μg/mL and 5.11 μg/mL, respectively. These results demonstrate that formulating CGA into composite micelles significantly reduces the IC50 value, enhancing the drug’s inhibitory effect on cell growth. The cell viability results for each preparation group are presented in Figure 16, showing a dose-dependent inhibitory effect on HepG2 cell growth.

At each time point, the three micelle groups exhibit greater inhibitory effects on cell viability compared to the solution group, indicating that incorporating curcumin into micelles enhances its tumor-inhibitory activity. In the CGA-GL group, cell viability falls below 80% when the drug concentration reaches 2 μg/mL, a statistically significant difference from the solution group (*p* < 0.05). As drug concentration increases further, the CGA/GA-GL and CGA-GL groups show progressively lower cell viability, with both groups exhibiting stronger inhibition compared to the solution group (*p* < 0.01). Although the GA-GL group also demonstrates lower cell viability than the solution group, the difference is not statistically significant.

Among the three micelle groups, the CGA/GA-GL and CGA-GL groups exhibit more potent inhibitory effects on cell viability when drug concentrations range from 4 to 16 μg/mL. Cell viability in these two groups is notably lower than that of the CUR sol group, likely due to the ability of GA to promote the cellular endocytosis of the micellar carrier, increasing intracellular drug concentration. Additionally, GA exhibits its own tumor-inhibitory properties, which synergize with curcumin to enhance therapeutic efficacy. This combined effect further strengthens the inhibition of tumor cell growth.

Within the concentration range investigated, the CGA-GL group consistently demonstrates lower cell viability compared to the CGA/GA-GL group. At a drug concentration of 16 μg/mL, the difference between the two groups becomes statistically significant (*p* < 0.001). This distinction may arise from the tumor microenvironment responsiveness of CGA-GL, which facilitates targeted drug release, thereby further enhancing the inhibitory effect on tumor cells.

### 2.8. In Vivo Distribution Study

A series of DIR iodide methanol solutions with varying concentrations were measured using a microplate reader. A linear regression analysis of the DIR solutions yielded a standard curve with the equation *y* = 2878.7*x* − 566.87 (*R*^2^ = 0.9998), indicating excellent linearity of the DIR iodide methanol solution under the analytical conditions in this experiment. The encapsulation efficiency (EE) and actual DIR concentration in different formulations were calculated using the standard curve, and the results are summarized in Table 5.

**Figure 17:**
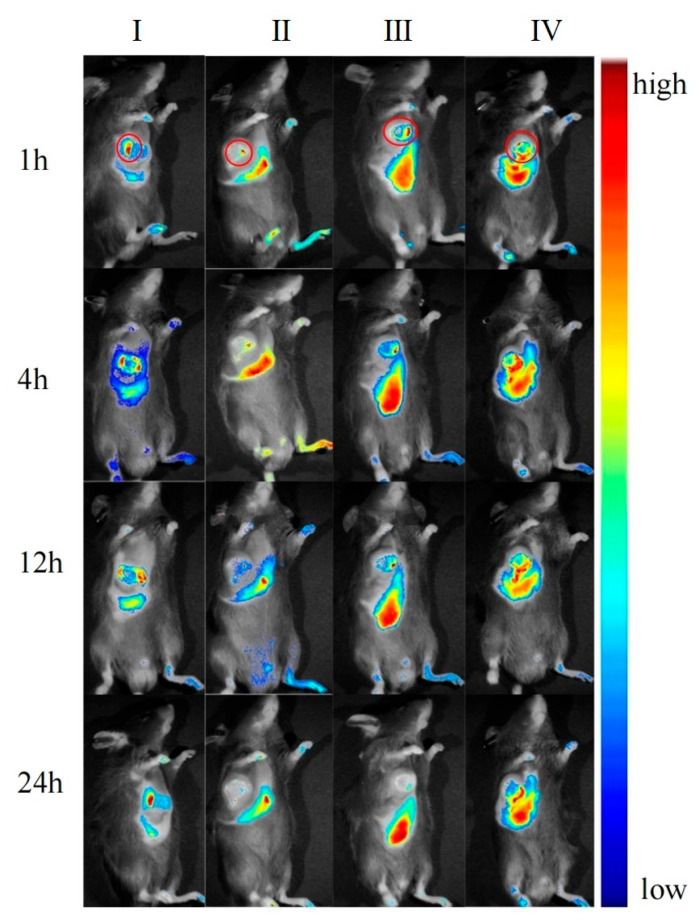
Real-time in vivo fluorescence imaging of DIR-labeled preparations (I: DIR solution group; II: DIR-GL micelles group; III: DIR/GA-GL micelles group; and IV: DIR/CGA-GL micelles group) within 24 h. (Circles: tumor site; *n* = 3).

**Figure 18:**
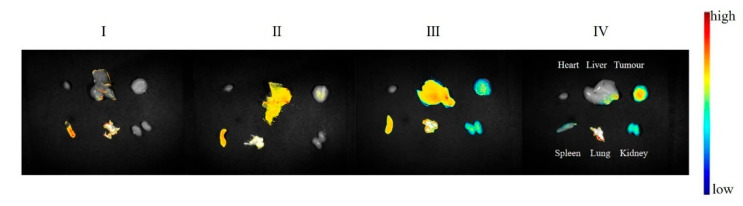
Ex vivo fluorescence imaging of major organs at 24 h post-injection (I: DIR solution group; II: DIR-GL micelles group; III: DIR/GA-GL micelles group; and IV: DIR/CGA-GL micelles group).

**Figure 19:**
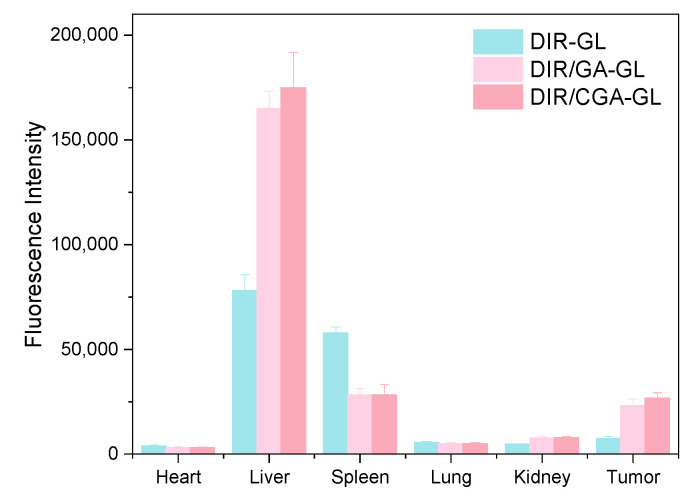
Ex vivo fluorescence intensity of DIR-labeled micelles in major organs and tumors at 24 h post-injection (*n* = 3).

After the preparations were injected into mice via the tail vein, the in vivo imaging results at different time points are shown in Figure 17. The ex vivo tissue fluorescence image and specific data are shown in Figure 18 and Figure 19. The fluorescence signal of the DIR solution group exhibited systemic distribution throughout the mice. Specific signal values were detected in tumor tissues and liver regions; however, these signals did not show an increasing or decreasing trend over time. This indicates that the DIR solution was rapidly metabolized and excreted within 1 h. Additionally, the observed signals in tumor tissues and liver regions may result from the spontaneous absorption of DIR in the subcutaneous blood vessels or organs.

In the DIR-GL group, the fluorescence signal was primarily distributed in the liver and tumor tissues, with fluorescence intensity increasing over the first 0–4 h. The signal gradually decreased over 12–24 h due to metabolism. Notably, the liver retained a strong fluorescence signal even after 12 h, demonstrating the excellent liver-targeting properties of GL.

Similarly, in the DIR/GA-GL group, fluorescence signals were primarily concentrated in the liver and tumor tissues, with fluorescence intensity increasing during the first 0–4 h and gradually decreasing over 12–24 h. The fluorescence intensity in the liver for this group was prominently higher than in the DIR-GL group after 12 h, indicating that the drug delivery system combining GA and GL provides stronger liver-targeting effects compared to the GL-only system.

The fluorescence distribution pattern in the DIR/CGA-GL group was largely consistent with that in the DIR-GL and DIR/GA-GL groups, with a similar trend in fluorescence intensity over time. This suggests that the CGA-GL system, prepared by modifying GA and CUR with a disulfide bond, retains the liver-targeting properties of GA and GL [59]. Furthermore, the CGA-GL system can direct therapeutic drugs to the tumor site and rapidly release CUR in response to the elevated GSH levels in the tumor microenvironment of liver cancer. This characteristic enhances the therapeutic efficacy of the drug, making it an effective liver-targeting drug delivery system.

### 2.9. In Vivo Antitumor Studies

**Figure 20:**
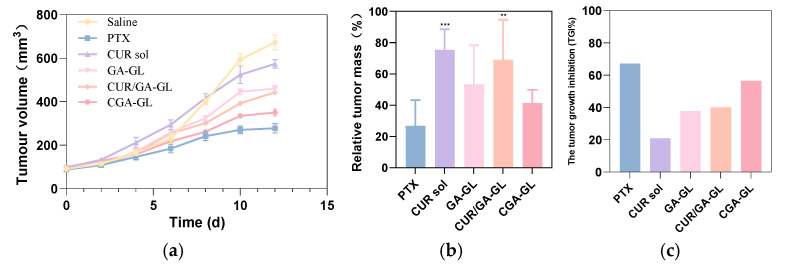
In vivo antitumor efficacy of BALB/c mice bearing H22 tumor cells. (**a**) Tumor volume kinetics over 12 days. (**b**) Relative tumor mass at endpoint (Day 12). (**c**) Tumor growth inhibition rate (TGI%). (*n* = 6, ** *p* < 0.01, and *** *p* < 0.001).

**Figure 21:**
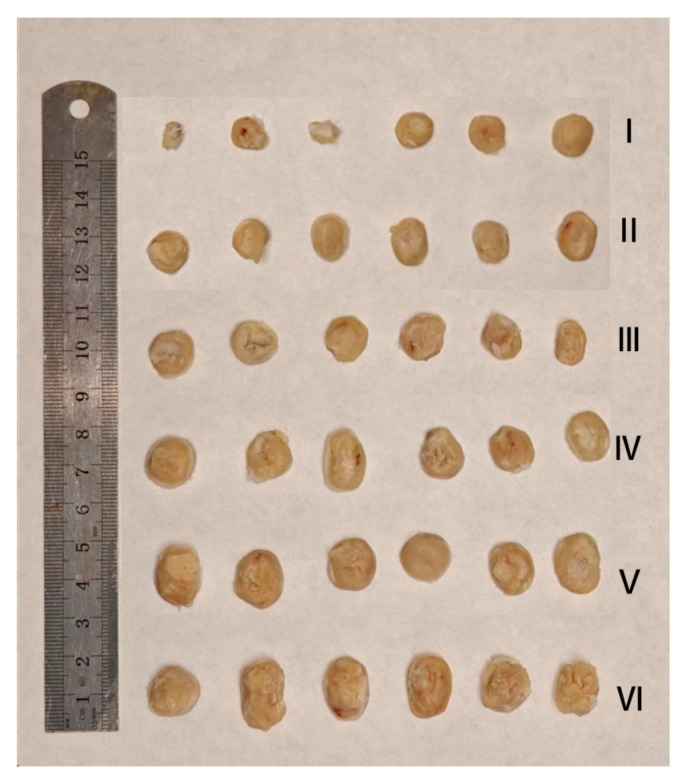
Tumor images and therapeutic efficacy validation after 12-day treatment. (I: PTX group; II: CGA-GL micelles group; III: GA-GL micelles group; IV: CUR/GA-GL micelles group; V: CUR sol; and VI: saline; *n* = 6).

As shown in Figure 20a–c and Figure 21, the tumor weight inhibition in this experiment aligns with the tumor volume change results. The tumor growth inhibition (TGI) rate in the CUR solution group is approximately 20%, and the relative tumor mass in this group is visibly different from the positive drug group. This indicates that the CUR solution exhibits only a weak therapeutic effect, likely due to the low solubility and bioavailability of curcumin.

In the CUR/GA-GL micelles group and the GA-GL blank carrier micelles group, the tumor growth inhibition rate and relative tumor mass show no significant differences, with tumor inhibition rates of approximately 40%. This suggests that GA and GL exhibit certain antitumor and targeting effects, but curcumin does not display a pronounced antitumor effect in these formulations.

By contrast, the CGA-GL micelles group achieves a tumor growth inhibition rate exceeding 50%, significantly higher than that of simply mixing curcumin and GA. This result demonstrates the advantage of synthetically linking curcumin and GA using GSH-responsive disulfide bonds, which enables the targeted release and accumulation of curcumin at tumor sites, thereby enhancing the antitumor effect. Notably, there is no significant difference in tumor growth inhibition rates between the CGA-GL micelles group and the positive drug group, highlighting the potential of CGA-GL micelles as a prospective antitumor drug delivery system.

**Figure 22:**
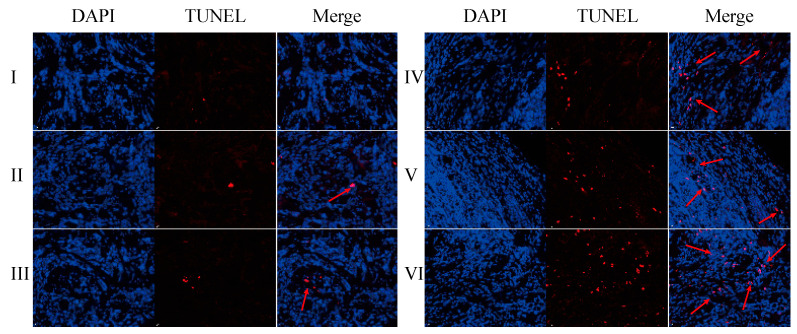
TUNEL assay evaluating H22 hepatoma cell apoptosis in tumor tissues. (I: saline; II: CUR solution group; III: GA-GL micelles group; IV: CUR/GA-GL micelles group; V: CGA-GL micelles group; and VI: PTX solution group. Arrows: red-positive nuclei; Scale bar: 100 μm).

The TUNEL assay results are displayed in Figure 22 and Figures S15–S20. Compared with the normal saline group, the GA-GL micelles, CGA/GA-GL micelles, CGA-GL micelles, and PTX-positive drug groups exhibit an increased number of red-positive nuclei, indicating a significantly enhanced apoptosis of liver cancer (H22) cells in these groups [60]. In contrast, the CUR solution group shows no significant difference from the saline group, failing to induce notable apoptosis of liver cancer cells.

The number of red-positive nuclei in the GA-GL micelles, CGA/GA-GL micelles, CGA-GL micelles, and PTX-positive drug groups is higher than in the CUR solution group. Among the three preparation groups, the number of red-positive nuclei follows the order CGA-GL micelles > CGA/GA-GL micelles > GA-GL micelles. These findings indicate that the GA/GL-based drug delivery system effectively targets and delivers therapeutic drugs to tumor sites. Additionally, modifying the disulfide bond in the CGA-GL system allows for more efficient and precise drug release at tumor sites, significantly enhancing the apoptosis of tumor cells.

### 2.10. Safety Evaluation

**Figure 23:**
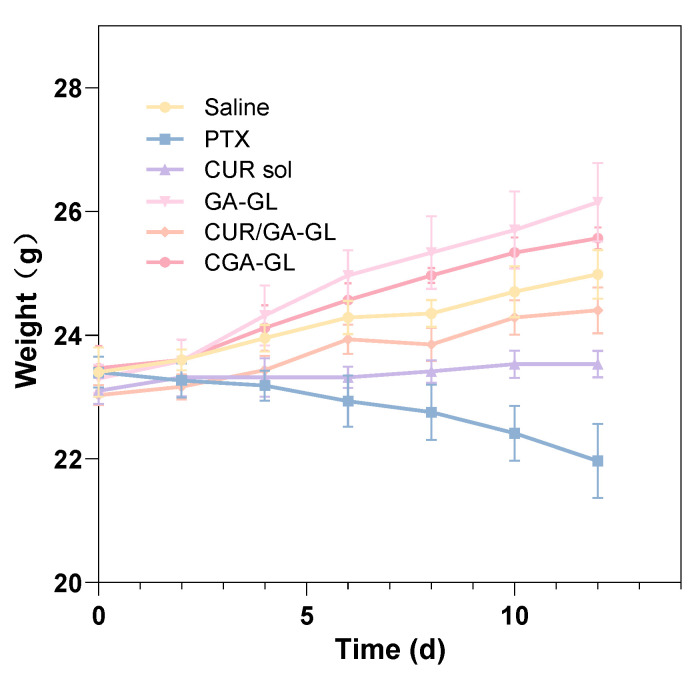
Body weight dynamics of H22 hepatoma-bearing mice during 12-day treatment (*n* = 6).

The weight changes of the mice are presented in Figure 23. At the start of drug administration, all groups displayed comparable body weights. Over the course of the study, mice in the positive drug group experienced significant weight loss with increasing administration frequency, while those in the CUR solution group maintained relatively stable weights. Conversely, the body weight of mice in the CGA-GL micelle group, saline group, CGA/GA-GL micelle group, and GA-GL micelle group exhibited varying degrees of increase. These data indicate that body weight is a useful marker for assessing the health of the mice. Relative to the saline group, the positive drug and CUR solution groups showed weight loss and growth inhibition, respectively, highlighting the systemic toxicity of these treatments. In contrast, none of the three micelle preparation groups adversely impacted the growth of mice.

**Figure 24:**
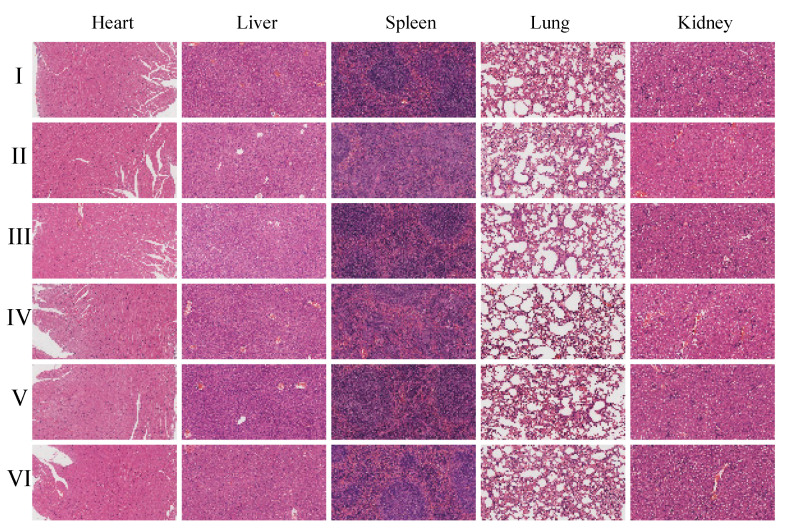
Histopathological analysis of major organs by H&E staining. (I: saline; II: PTX solution; III: CUR solution; IV: GA-GL micelles; V: CUR/GA-GL micelles; and VI: CGA-GL micelles. Scale bar: 100 μm).

Histopathological examination using hematoxylin and eosin (H&E) staining (Figure 24 and Figures S21–S26) further supports these findings, as no pathological changes were observed in the organs of the CGA-GL group. Similarly, no significant differences in histological results were observed among the CGA-GL, CGA/GA-GL, and GA-GL micelle groups compared to the saline group, confirming the absence of systemic toxicity in these micelle formulations.

**Figure 25:**
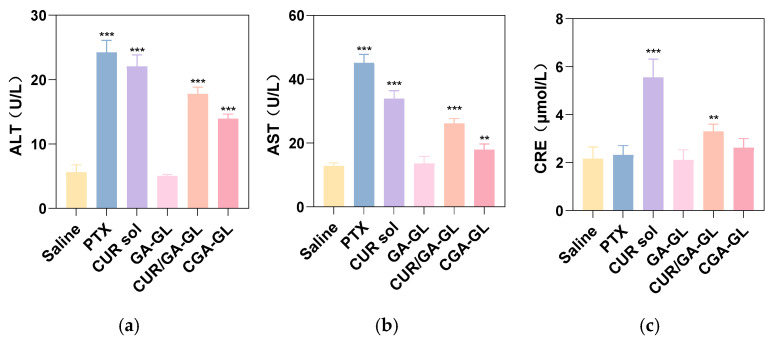
Serum biochemical indices for hepatotoxicity and nephrotoxicity evaluation. (**a**) Aspartate aminotransferase (AST) analysis results. (**b**) Alanine aminotransferase (ALT) analysis results. (**c**) Creatinine (CRE) analysis results. (*n* = 6, ** *p* < 0.01, and *** *p* < 0.001).

Serum AST/ALT levels, illustrated in Figure 25a,b, were significantly elevated in the positive drug, CUR solution, CGA-GL micelle, and CGA/GA-GL micelle groups relative to the saline group. These results suggest that all tested groups, except for the GA-GL micelle group, caused varying degrees of hepatotoxicity. This hepatotoxicity is likely attributable to the intrinsic toxicity of CUR and the positive drug (paclitaxel) upon entering the bloodstream. Among the micelle preparations, the CGA/GA-GL micelle group demonstrated reduced AST/ALT levels compared to the CUR solution group, with the CGA-GL micelle group exhibiting the lowest levels. These findings suggest that the GA-GL system confers hepatoprotective effects. Moreover, the introduction of disulfide bonds facilitates tumor-specific CUR release, enhancing its bioavailability and distribution while minimizing liver toxicity. As depicted in Figure 25c, serum creatinine levels increased to varying degrees in the positive drug, CUR solution, CGA-GL micelle, and CUR/GA-GL micelle groups compared to the saline group. However, the GA-GL micelle group maintains creatinine levels comparable to those of the saline group. Notably, serum creatinine levels in the CUR solution group (*p* < 0.05) and CUR/GA-GL micelle group (*p* < 0.001) were remarkably higher than those in the saline group, whereas no significant difference was observed between the CGA-GL micelle and saline groups. Additionally, creatinine levels decreased sequentially from the CUR solution group to the CUR/GA-GL micelle group and finally to the CGA-GL micelle group. These findings underscore the ability of CGA-GL micelles, constructed by linking CUR and GA via disulfide bonds in the CGA prodrug, to further mitigate CUR-associated nephrotoxicity.

## 3. Discussion

The current study proposes a novel micellar system based on the conjugation of curcumin (CUR) with glycyrrhetinic acid (GA)—a hepatocyte-targeting ligand—via a glutathione (GSH)-sensitive disulfide bond to form a curcumin prodrug (CGA). This prodrug is then self-assembled with glycyrrhizic acid (GL) to create a multi-functional micellar system addressing CUR’s poor aqueous solubility, hepatic targeting, and tumor-specific drug release. Below is a detailed interpretation of this strategy and its advancements compared to existing research:

The conjugation of CUR with GA through a redox-sensitive disulfide bond can enhance hepatic targeting and control drug release. GA, a natural triterpenoid from licorice, not only serves as a ligand for active hepatic targeting but also acts as a structural component of the micellar system. GA specifically binds to GA receptors overexpressed on the hepatocyte membrane, and it has been widely explored in different drug systems [61]. The 1.8-fold enhanced cellular uptake of CGA-GL micelles (vs. free CUR) directly correlates with GA receptor overexpression in HepG2 cells, consistent with prior studies using GA-functionalized liposomes. Numerous studies have utilized the co-modification of GA with poly ethylene glycol (PEG) in drug carriers such as liposomes, micelles, or nanoparticles to enhance drug accumulation in HCC tumors [62]. Liposomes prepared with synthetic DSPE-mPEG2000-GA for delivering CUR exhibit higher cytotoxicity compared to GA ligand-free liposomes, the hepatic targeting ability of GA enhances the cellular uptake of liposomes in HCC cells via GA receptor-mediated endocytosis, increasing intracellular CUR concentration and cytotoxicity [63]. The micellar system constituted by GA coupling PEG-disulfide linkage-poly (lactic-co-glycolic acid) (GA-PEG-SS-PLGA) to encapsulate Tanshinone IIA (TAN IIA) has increased accumulation in the liver, inhibiting tumor growth and increasing the survival rate of mice [64].

However, drug delivery systems incorporating synthetic materials such as PEG or PLGA may exhibit inherent toxicity and potential safety concerns due to risks like immunogenicity and non-biodegradability, while delivery systems entirely composed of natural bioactive components remain scarce [65]. In contrast, this underscores the distinct advantages and limited exploration of natural micelles formed by conjugating CUR and GA, which integrate therapeutic and structural functions within a fully natural framework. The CGA-GL micelles exclusively consist of endogenous bioactive compounds—GL, GA, and CUR—with complete absence of synthetic polymers or surfactants. Functionally, GL alleviates hepatic inflammation and oxidative stress within the liver microenvironment; GA directly triggers tumor apoptosis while suppressing fibrotic progression; and CUR coordinates multi-pathway anticancer mechanisms. Critically, these constituents serve dual roles as both structural elements and therapeutic agents, maintaining micelle structure while delivering therapeutic benefits. Their synergistic therapeutic effects against HCC establish an innovative drug-carrier integration system where formulation components concurrently enable both pharmaceutical delivery and targeted biological action [44,66]. The 58.7% tumor suppression in H22 hepatoma models, coupled with 3.6-fold higher tumor accumulation than non-targeted controls, underscores the translational potential of this system.

Additionally, this structural design of CGA exhibits key bio-responsive features: The disulfide bridge is susceptible to cleavage in a reductive biological environment, particularly under high GSH concentrations [67]. This mechanism is expected to enable targeted drug release and controlled activation. GSH-overexpressing tumor cells like hepatocellular carcinoma can specifically reduce the disulfide bond, releasing free CUR and GA. The GSH-dependent release profile (67.5% vs. <40% without GSH) validates the disulfide bond’s role in minimizing premature leakage while enabling tumor-selective CUR activation, a critical advantage over pH- or enzyme-responsive systems with off-target release risks. The intact conjugate may exhibit reduced systemic toxicity, while the cleaved products (CUR and GA) regain their pharmacological activities at target sites.

In particular, CUR’s hydrophobicity has historically limited its drug loading capacity; the loading efficiency is less than 20% in most formulations. The drug loading of CUR in liposomes modified with rhamnose is 3.5% [68] and the loading efficiency of CUR nanoparticles composed of a saponin coating formed by a pH-driven loading method is 15.3% [69]. Using advanced materials such as metal–organic frameworks (MOFs) can improve the encapsulation efficiency to some extent. The loading efficiency of the Zr-based MOF UiO-66-loaded CUR is 3.45% [70], and the results for CUR loaded on γ-cyclodextrin-metal–organic-frameworks (γ-CD-MOFs) can reach 17.98% [71]. However, MOF-based carriers face several critical limitations that hinder their clinical translation, such as synthesis complexity and toxicity risks [72]. Furthermore, even in studies utilizing advanced materials, the CUR loading capacity achieved in those systems remains lower than the nearly 26% encapsulation efficiency attained by our delivery system.

While the findings of this study are encouraging, several critical limitations remain. First, the in vivo pharmacokinetic behavior of the CGA-GL micelles has not been fully elucidated. Additionally, the current therapeutic validation is limited to the H22 hepatoma xenograft model, which fails to fully recapitulate the high heterogeneity and fibrotic microenvironment of human hepatocellular carcinoma. Future work should validate therapeutic potential in patient-derived xenograft (PDX) models or orthotopic liver cancer models that better mimic clinical pathology.

To address these limitations, future research should prioritize the following directions: exploring the co-delivery of curcumin with chemotherapeutic agents (e.g., sorafenib) via CGA-GL micelles to leverage synergistic effects and overcome multidrug resistance in liver cancer; developing Good Manufacturing Practice (GMP)-compliant scalable production processes to minimize batch-to-batch variability in natural components and facilitate clinical translation; and investigating the immunomodulatory roles of glycyrrhizic acid (GL) and glycyrrhetinic acid (GA) in regulating the hepatic immune microenvironment (e.g., Kupffer cell polarization and T-cell infiltration) to expand the system’s immunotherapy capabilities. These efforts will strengthen the scientific foundation and clinical applicability of this natural micellar system, offering a transformative strategy for hepatocellular carcinoma treatment.

## 4. Materials and Methods

### 4.1. Materials

Curcumin (Cur, >98%), glycyrrhetinic acid (GA, >95%), and glycyrrhizic acid (GL, >90%) were obtained from Shanghai Yuanye Bio-Technology Co., Ltd. (Shanghai, China). 2-(7-Aza-1H-benzotriazole)-N, N, N’, N’-tetramethyluronium hexafluorophosphate (HATU) was acquired from Reinnovo Environmental Technology Co., Ltd. (Nantong, China). N, N-Dimethylformamide (DMF) and 1,4-dioxane and hydrochloric acid (HCL) were obtained from Sigma-Aldrich Trading Co., Ltd. (Shanghai, China). N, N-Diisopropylethylamine (DIEA) was sourced from NJ Peptide Biotechnology Co., Ltd. (Nanjing, China). Methanol (MeOH) and acetonitrile (ACN) were purchased from Thermo Fisher Scientific Co., Ltd. (Waltham, MA, USA). Triethylamine (TEA), di-tert-butyl dicarbonate ((Boc)_2_O), dichloromethane (DCM), diethyl ether (DEGME), ethyl acetate (EAC) and dimethyl sulfoxide (DMSO) were purchased from Meryer Chemical Technology Co., Ltd. (Shanghai, China). Reduced glutathione (GSH) was provided by Aladdin Biochemical Technology Co., Ltd. (Shanghai, China). DIR iodide was purchased from Meilun Biotechnology Co., Ltd. (Dalian, China). Serum alanine aminotransferase (ALT), aspartate aminotransferase (AST), and creatinine (CRE) levels were determined at the Institute of Bioengineering (Nanjing, China). All solvents were HPLC grade.

### 4.2. Synthesis of CUR-GA Polymer (CGA)

**Figure 26:**
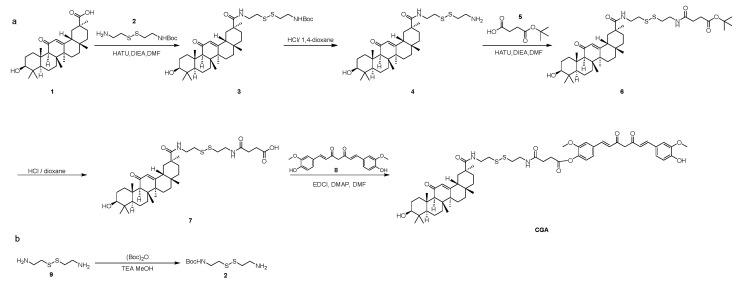
Synthesis route of (**a**) CUR-GA and (**b**) Compound 2.

The CUR-GA polymer (CGA) was synthesized via a GSH-sensitive disulfide bond. Figure 26 shows the synthesis route. The synthesis process was divided into several steps, as follows.

Synthesis of Compound 2: Compound 9 (20.00 g, 131.33 mmol) was dissolved in MeOH (100 mL), and TEA (73.01 mL, 525.31 mmol) and (Boc)_2_O (28.10 mL, 131.33 mmol) were added at 0 °C. The mixture was stirred at 20 °C for 1 h. TLC (10%MeOH in DCM, Rf = 0.2) revealed the formation of a new spot. The solid was obtained in 70 mL of a 1M solution of NaH_2_PO_4_. The mixture was then extracted with DEGME (3 × 90 mL). The aqueous phase was adjusted to pH 9 using a 1M NaOH solution. The mixture was then extracted with EAC (6 × 50 mL). The organic phases were concentrated to obtain Compound 2 (6.60 g, 26.15 mmol, 19.91%) as a white solid.

Synthesis of Compound 3: Compound 1 (GL) (12.00 g, 25.49 mmol) was dissolved in DMF (100 mL), and DIEA (12.64 mL, 76.48 mmol) and HATU (9.69 g, 25.49 mmol) were added. Compound 2 (6.44 g, 25.49 mmol) was introduced, and the reaction was stirred at 25 °C for 1 h. TLC (5% MeOH in DCM, Rf = 0.6) showed a new spot. The mixture was washed with EAC (3 × 100 mL) and 50 mL water. The organic phases were concentrated and purified by silica chromatography (0–3% MeOH in DCM) to obtain Compound 3 (10.00 g, 14.18 mmol, 55.63%) as a white solid.

Synthesis of Compound 4: Compound 3 (10.00 g, 14.18 mmol) was dissolved in DCM (15 mL), and 4 M HCl/dioxane (100 mL) was added at 0 °C. The reaction was stirred at 25 °C for 1.5 h. TLC (10% MeOH in DCM) showed no raw material. The mixture was concentrated to obtain Compound 4 (8.00 g, 13.22 mmol, 93.24%) as a white solid.

Synthesis of Compound 6: Compound 5 (1.78 g, 10.21 mmol), DIEA (9.37 mL, 56.70 mmol), and HATU (5.17 g, 13.61 mmol) were dissolved in DMF (80 mL), and Compound 4 (6.86 g, 11.34 mmol) was added. The reaction was stirred at 25 °C for 1 h. TLC (10% MeOH in DCM, Rf = 0.6) showed a new spot. The mixture was washed with EAC (3 × 100 mL) and 50 mL water. The organic phases were concentrated and purified by silica chromatography (0–3% methanol in dichloromethane) to obtain Compound 6 (5.00 g, 6.57 mmol, 57.93%) as a brown solid.

Synthesis of Compound 7: To a solution of Compound 6 (5.00 g, 6.57 mmol) in DCM (15 mL), HCl/1,4-dioxane (80 mL) was added at 0 °C. The reaction was stirred at 25 °C for 1.5 h. TLC (10% MeOH in DCM) showed no raw material. The mixture was concentrated and purified by pre-HPLC (5–65% ACN in (0.1%TFA) water) to obtain Compound 7 (2.50 g, 3.55 mmol, 53.98%) as a brown solid.

Synthesis of CUR-GA Polymer (CGA): Compound 7 (250 mg, 0.355 mmol), DMAP (9 mg, 0.071 mmol), and EDCI (102 mg, 0.532 mmol) were dissolved in DMF (3 mL), and Compound 8 (CUR) (0.196 mg, 0.532 mmol) was added at 25 °C. The reaction was stirred at 25 °C for 1.5 h. TLC (10% MeOH in DCM, Rf = 0.6) showed a new spot. The mixture was washed with EAC (3 × 30 mL) and 10 mL water. The mixture was concentrated and purified by pre-HPLC (5–75% ACN in (0.1%TFA) water) to obtain CUR-GA polymer (CGA) (70 mg, 0.07 mmol, 23.38%) as a yellow solid.

### 4.3. Fourier Transform Infrared (FTIR) Spectroscopy

FTIR spectra were recorded using a Nicolet iS10 FTIR (Thermo Nicolet Corporation, Waltham, MA, USA) spectrometer equipped with a DTGS detector. To investigate the structure, CGA, CUR, GA, and the physical mixture CUA and GA, samples were prepared respectively by grinding drugs with KBr (1:150 *w*/*w*) and pressing into pellets. Spectral data were collected in the range of 4000–400 cm⁻^1^ at a resolution of 4 cm⁻^1^ with 32 scans per sample. Background scans were performed prior to each measurement. Raw spectra were processed using OPUS 7.0 software, including baseline correction and smoothing.

### 4.4. ^1^H Nuclear Magnetic Resonance (1H-NMR) Analysis

^1^H-NMR spectra of CGA were recorded on a Bruker AVANCE III 600 MHz spectrometer (Bruker Corporation, Billerica, MA, USA) equipped with a 5 mm TCI cryoprobe. Approximately 20 mg of CGA powder was dissolved in 0.6 mL deuterated dimethyl sulfoxide (DMSO-d _6_) in an NMR tube. The mixture was vortexed for 2 min to ensure complete dissolution. Spectra were acquired at 298 K with a spectral width of 12 ppm, a relaxation delay (D1) of 2 s, and 16 scans per sample. The residual solvent peak (DMSO-d_6_ at δ 2.50 ppm) was used as an internal reference. Raw data were processed using TopSpin 4.0 software (Bruker) with exponential line broadening (0.3 Hz) and baseline correction. The characteristic proton signals of CGA were compared with reference spectra from literature to confirm its structural integrity.

### 4.5. Parameters for HPLC-MS Analysis

Compound purity was analyzed by High Performance Liquid Chromatography (HPLC) using an Agilent 1260 system (1260 Infinity II HPLC, Agilent Technologies, Inc., Palo Alto, CA, USA) equipped with a Shim-pack GIST C18 column (4.6 × 250 mm, 5 μm, P/N. 227–30017-08, S/N. 18L09593). The chromatographic separation was carried out using a mobile phase comprising 0.03% trifluoroacetic acid (TFA) in water (A) and 0.03% TFA in acetonitrile (B) under isocratic elution conditions (30% A and 70% B) for 30 min. Detection was performed at 254 nm with a flow rate of 1 mL/min, while the column temperature was maintained at 25 °C. Each sample (10 μL injection volume) was followed by a 2 min post-run re-equilibration to ensure column stability. The validity of the HPLC method was demonstrated by conducting tests on linearity, precision, stability, specificity, the limit of detection, and the limit of quantitation. To confirm the absence of interference from micelle components (e.g., GL) during drug quantification, blank micelles (GA-GL micelles) were analyzed under identical HPLC conditions. No peaks corresponding to CGA or CUR were observed in the blank micelle chromatogram, confirming the specificity of the method for drug detection.

For structural confirmation, Liquid Chromatograph-Mass Spectrometer (LC-MS) was performed on Agilent Technologies 6460 Triple Quad LC/MS (Agilent Technologies, Inc., Palo Alto, CA, USA) with a Waters Sun Fire C18 column (50 × 4.6 mm, 5 μm). The analysis was performed in TIC (positive ion mode) with the following conditions: column temperature maintained at 40 °C, a gradient elution program starting at 5% B (0.1% TFA in ACN) for 0.2 min, ramping up to 95% B over 1.40 min, holding at 95% B for 0.9 min, and returning to 5% B in 0.01 min for re-equilibration, accompanied by mobile phase A consisting of 0.1% TFA in H _2_O and an injection volume of 5 µL. The scan mode was full scan (*m*/*z* 100–1500), with capillary voltage: 3.5 kV, nebulizer gas pressure: 35 psi; drying gas flow: 10 L/min, and gas temperature: 350 °C.

### 4.6. Preparation of Micelle Nanoparticles

Drug-loaded micelles (CGA-GL micelles and CUR/GA-GL micelles) were prepared using the solvent evaporation method, as described in the literature [73]. CGA (or CUR and GA) was dissolved in MeOH to form the organic phase, which was then subjected to ultrasonic treatment under dark conditions. GL was dissolved in ultrapure water to form the aqueous phase, and ultrasonic mixing was applied. The organic phase was added dropwise to the aqueous phase under magnetic stirring (400 rpm, 25 °C) for 1 h, allowing the complete evaporation of MeOH, which resulted in the formation of CGA-GL (or CUR/GA-GL) micelles. Blank micelles (without drug) were synthesized following the same procedure as drug-loaded micelles, omitting the addition of CGA or CUR; these were used exclusively to validate the absence of drug-related signals in HPLC analysis.

### 4.7. Characterization of Micelles

The micelles were suspended in deionized water for particle size, polydispersity index (PDI), and zeta potential (ζ), determined using a Malvern Zeta Sizer Nano Series (Malvern Instruments Ltd., Malvern, Worcestershire, UK) at 25 °C. Triplicate measurements were performed, with results expressed as mean ± SD. Micelle morphology was observed via transmission electron microscopy (TEM) (Tecnai G2 F30, FEI Company, Hillsboro, OR, USA).

### 4.8. In Vitro Drug Loading

The drug loading capacity (DLC) and encapsulation efficiency (EE) of CGA and CUR in the micelles (CGA-GL micelles and CUR/GA-GL micelles) were determined using the microcolumn centrifugation method. Blank micelles (GA-GL micelles) were not subjected to DLC/EE calculations, as they contained no drug. Sephadex G-50 was packed into a 2.5 mL syringe to form a 2 mL gel column. The drug-loaded micelles were eluted with water at 1500 rpm for 5 min. The amount of free CUR/CGA in the eluent was quantified by HPLC. DLC and EE were calculated using the following Equations (3) and (4):

(3) $$EE=\frac{\;\mathrm{the}\;\mathrm{total}\;\mathrm{amount}\;\mathrm{of}\;\mathrm{drug}\;-\;\mathrm{free}\;\mathrm{amount}\;\mathrm{of}\;\mathrm{drug}\;}{\;\mathrm{the}\;\mathrm{total}\;\mathrm{amount}\;\mathrm{of}\;\mathrm{drug}\;}\times 100\%$$

(4) $$DL=\frac{\;\mathrm{the}\;\mathrm{total}\;\mathrm{amount}\;\mathrm{of}\;\mathrm{drug}\;-\;\mathrm{free}\;\mathrm{amount}\;\mathrm{of}\;\mathrm{drug}\;}{\;\mathrm{the}\;\mathrm{total}\;\mathrm{amount}\;\mathrm{of}\;\mathrm{micelles}\;}\times 100\%$$

### 4.9. In Vitro Drug Release

Drug release profiles of CUR solution, CGA-GL micelles, and CUR/GA-GL micelles were evaluated using the dialysis method (*n* = 3) [74]. Dialysis bags (MW cutoff, 8–10 kDa) containing the drug solutions were immersed in PBS (pH 7.4 or pH 5.0, containing 1% Tween 80) with 0, 5, or 10 mM GSH at 37 ± 0.5 °C, stirred at 100 rpm [75]. At specified time intervals (0.5–24 h), 1 mL of release medium was withdrawn and replaced with fresh PBS. Drug concentrations in the medium were quantified by HPLC. The cumulative release rate was calculated using the following Equation (5):

(5) $$\mathrm{Cumulative}\;\mathrm{release}\;\mathrm{rate}=\frac{C_{n}\times V1+V2\times \sum_{i=1}^{n-1}C_{i}}{M}\times 100\%$$

Cn represents the drug concentration in the release medium at the nth sampling time point; V1 represents the total volume of the released medium; V2 represents the extracted medium; and M represents the quality of CGA or CUR in the dialysis bag.

### 4.10. Determination of Critical Micelle Concentration (CMC)

The CMC values of CGA-GL, GA-GL, and CUR/GA-GL micelles were determined using a conductivity meter (DDS-307A, INESA Scientific Instrument Co., China) equipped with a platinum conductivity cell (cell constant = 0.978 cm⁻^1^) [53]. Aqueous dispersions of each micelle system were prepared in deionized water at initial concentrations (calculated by GL) approaching 1200 μg/mL. Serial dilutions were performed to obtain concentration gradients spanning 0.1–1200 μg/mL; all solutions were equilibrated at 25 °C for 30 min prior to measurement. The CMC was identified as the inflection point in the κ vs. C plot, determined by intersecting linear regressions of the pre- and post-micellization regions using Origin 2021. Results are expressed as mean ± SD (*n* = 3 independent batches).

### 4.11. Animals and Culture

Human hepatocellular carcinoma cells HepG2 cells were kindly provided by the Cell Bank, Chinese Academy of Sciences (Shanghai, China), and cultured in DMEM supplemented with 10% FBS, HEPES (25 mM), NaHCO_3_ (3.7 g/L), penicillin (100 U/mL), and streptomycin (100 g/mL). H22 cells were obtained from the Institute of Basic Medical Sciences of the Chinese Academy of Medical Sciences (Beijing, China). H22 cells were grown in a HyClone RPMI 1640 medium containing 10% FBS, 100 U/mL penicillin, and 100 μg/mL streptomycin. All the cells were incubated in humidified incubator containing 5% CO_2_ and 95% air in an incubator at 37 °C. Cells were maintained in the logarithmic growth phase by routine passaging every 3 days. BALB/c mice (male, 4–6 weeks, 15 ± 1 g) were purchased from SPF Biotechnology Co., Ltd. (Beijing, China). The animal experiments and feeding were carried out under SPF conditions, and the contents of animal experiments were in line with animal ethical standards.

The animal experimental protocols were approved by the Ethics Committee of Tianjin University of Traditional Chinese Medicine (Document number: TCM-LAEC2023142).

### 4.12. In Vitro Cell Uptake Assay

The High Content Screening system is used to quantitatively monitor the absorption of drug-loaded micelles inside cells. Inoculate HepG2 cells in the logarithmic growth phase into a 96-well blackboard, with approximately 8 × 103 cells per well, and incubate in an incubator for 24 h. After incubation, aspirate the culture medium and set up groups, add 100 μL of cell culture medium containing Cou6 sol, Cou6-GL, Cou6/GA-GL, and Cou6/CGA-GL to each well (the concentration of Cou6 is 0.05 μ g/mL), and set the culture time to 2 h, 4 h, and 6 h in an incubator at 37 °C. Add 50 μL (10 μg/mL) of Hoechst 33342 (Thermo Fisher Scientific Inc., Shanghai, China) solution to each well to stain the cell nucleus, incubate in a 37 °C incubator for 30 min, then aspirate the solution from the well and terminate uptake. Wash the cells twice with PBS to remove the fluorescent float and observe their drug uptake during the above periods. Place the processed 96 well plates into the high-content imaging system and set the excitation wavelength of the nuclear dye Hoechst 33342 at 346 nm, the emission wavelength at 460 nm, the excitation wavelength of Cou6 at 360 nm, and the emission wavelength at 477 nm. Calculate the average fluorescence intensity of the images in each well using Columbus (PerkinElmer) to qualitatively and quantitatively analyze the uptake of cells. The entire experiment needs to be conducted under dark conditions.

Flow cytometry (FCM) analysis evaluates cells’ absorption of drug-loaded micelles in vitro. HepG2 cells in the logarithmic growth phase were seeded at 1 × 105/well density into a 12-well plate and cultured in an incubator for 24 h. Remove the original culture medium and add 2 mL of Cou6 sol, Cou6-GL, Cou6/GA-GL, and Cou6/CGA-GL to each well (the concentration of Cou6 is 0.05 μg/mL). Incubate HepG2 cells with added drugs in a 37 °C incubator for 2, 4, and 6 h, then aspirate the culture medium. Wash the cells twice with PBS, digested with 0.25% trypsin EDTA, centrifuge at 800 rpm for 5 min, and wash the cell pellet twice with PBS. Add 500 μL of PBS to the washed cells, vortex for 30 s to resuspend them, and measure the fluorescence content using a Beckman Coulter EPICS XL flow cytometer.

### 4.13. Cytotoxicity Assay

HepG2 cells were seeded into 96-well plates at a density of 8 × 10^3^ cells/well (100 μL/well) and incubated for 24 h at 37 °C under 5% CO _2_. After removing the culture medium, the cells were treated with four formulations: CUR sol, CUR-GL, CUR/GA-GL, and CGA-GL, at concentrations ranging from 1 to 16 μg/mL (1, 2, 4, 8, and 16 μg/mL). The control group received complete DMEM medium. Following 24 h of incubation, 1 mg/mL MTT solution (prepared in DMEM) was added to each well, and the cells were further incubated for 4 h. The formazan crystals formed were dissolved in 150 μL DMSO after low-speed shaking at 37 °C for 10 min. The absorbance of each well was measured at 490 nm using a microplate reader (TECAN, Männedorf, Switzerland). Cell viability was calculated as (OD treatment/OD control) × 100%.

### 4.14. In Vivo Imaging and Bio-Distribution Analysis

The micelles’ biological distribution and tumor accumulation after tail vein injection were investigated using an in vivo imaging system (IN-VIVO MASTER, Best-biosystems Technology Co., Ltd., Beijing, China). To develop a hepatocellular carcinoma-bearing, ectopic transplanted tumor, an H22 cell suspension (2 × 10^7^/mL, PBS, 0.1 mL) was injected subcutaneously into the right flank of male BALB/c mice [76], and the tumor volume was monitored and measured every two days until reaching approximately 200 mm^3^ (a: tumor width, b: tumor length, and tumor volume V = ab^2^/2) [77]. After two weeks, the tumor volume approximately reached 200 mm^3^, and mice were randomly divided into four groups (*n* = 3), each labeled separately. The four groups were divided explicitly into a DIR solution group, DIR-GL micelles group, DIR/CGA-GL micelles group, and DIR/GA-GL micelles group. The DIR fluorescent dye was administered at a loading concentration of 0.5 mg/kg [78]. Based on individual body weight, each group of HCC model mice received a 0.2 mL injection of either DIR solution or DIR-loaded formulations via the tail vein [79]. At 1, 4, 12, and 24 h post-injection, mice were anesthetized with isoflurane, and fluorescence images were acquired [80]. The mice were placed in an in vivo imaging system under detection conditions (excitation: 710 nm; emission: 800 nm). After 24 h of injecting the preparation, all four groups of HCC model mice were euthanized. Major organs (heart, spleen, lung, liver, kidney) and tumor tissues were surgically dissected, followed by vascular perfusion with physiological saline to remove residual blood. Subsequently, the processed samples were subjected to ex vivo fluorescence imaging using the imaging system to compare the biodistribution profiles of different formulations across organs and tumor sites.

### 4.15. In Vivo Antitumor Evaluation

Hepatoma-bearing mice with H22 cell-derived tumors were used, and the tumor inoculation method followed the protocol described in Section 4.10. After 3 days of inoculating cells, tumor formation was observed in most of the mice, indicating the appropriate molding process. The tumor volume was monitored and measured every two days; after one week of inoculating, the size of the tumor had grown to approximately 100 mm^3^ [81], and treatment administration was initiated, with the first day of dosing designated as Day 0 (Baseline measurements were recorded prior to Day 0). The experiment was conducted using the method of administering drugs every two days, that is, measuring tumor volume size and administering drugs on Days 0, 2, 4, 6, 8, 10, and 12 [82]. Mice successfully loaded with tumors were divided into six groups (*n* = 6), and the specific groups were as follows: paclitaxel solution (positive drug, PTX), CGA-GL micelles, CUR solution, CUR/GA-GL micelles, GA-GL micelles, and saline group. Each group of mice was labeled with mouse ear tags, and the dosage was calculated based on body weight. Each group of mice was injected with 0.2 mL of different micelle preparations or solutions through the tail vein [79]. In contrast, the control group was injected with the same volume of physiological saline. Among them, the dose of CUR in each group is 10 mg/kg, the dosage of GA in the CUR/GA-GL group and GA-GL group was calculated based on the actual dosage of GA in the CGA-GL group [83].

Each mouse’s body weight and tumor volume were weighed and recorded during administration. The weight and tumor volume time curves were plotted after six administrations to analyze the physiological status of the mice and evaluate the efficacy and toxicity of the test formulation.

After the last administration, the mouse blood and centrifuge were collected to separate the serum, and then the mice were euthanized. ELISA kits detect AST, ALT, and CRE and investigate each group’s toxicity to the liver and kidneys. Weigh the tumor tissues of each mouse and calculate the relative tumor mass and tumor growth inhibition (TGI). They were calculated according to the following Equations (6) and (7):

(6) $$\mathrm{relative}\;\mathrm{tumor}\;\mathrm{mass}=\frac{\mathrm{the}\;\mathrm{tumor}\;\mathrm{weight}\;\mathrm{of}\;\mathrm{each}\;\mathrm{experimental}\;\mathrm{group}}{\mathrm{the}\;\mathrm{average}\;\mathrm{tumor}\;\mathrm{weight}\;\mathrm{of}\;\mathrm{the}\;\mathrm{control}\;\mathrm{group}}\times 100\%$$

(7) $$\mathrm{TGI}=(1-\frac{T-T_{0}}{C-C_{0}})\times 100\%$$

*T* and *T*_0_ represent the average tumor volume of the treatment group on the last day of administration and before administration, respectively. *C* and *C*_0_ represent the average tumor volume of the control group on the last day of administration and before administration, respectively.

Further paraffin-embedded sections were made after soaking and fixing the mouse tumor tissue and significant organs in 4% tissue/cell paraformaldehyde fixative. The apoptosis of tumor tissues was evaluated using the TUNEL detection method. At the same time, a histological analysis of major organs was performed using hematoxylin and eosin (H&E) staining. 

### 4.16. Statistical Analyses

The Shapiro–Wilk test indicated no significant deviation from normality (*p* > 0.05); parametric post-hoc tests were applied. GraphPad Prism 9.0 software was used to analyze the data. All results were presented as mean ± standard deviation (SD). Statistical significance was analyzed using a significance *t*-test or a one-way analysis of variance (ANOVA) (* *p* < 0.05, ** *p* < 0.01, and *** *p* < 0.001).

## 5. Conclusions

This study demonstrates that a fully natural, GA/GL-based micellar system (CGA-GL micelles) effectively overcomes three key barriers limiting curcumin’s clinical utility: poor solubility, off-target biodistribution, and uncontrolled release. Specifically, it achieves a curcumin concentration of 1.04 mg/mL, exhibits 3.6-fold higher tumor accumulation compared to controls, and shows 67.5% GSH-triggered drug release, significantly higher than the baseline of less than 40%. By integrating GA’s intrinsic targeting with GL’s self-assembly capability and a tumor-responsive disulfide bond, the system enables synergistic drug delivery and carrier-mediated targeting. Although preliminary in vitro and in vivo results are promising, clinical translation requires scaling production under GMP standards and evaluating immunomodulatory effects. This natural prodrug design provides a biocompatible and scalable platform for liver-targeted therapies, bridging the gap between synthetic nanocarriers and clinically translatable natural systems.

## Figures and Tables

**Table 1:** Methodology validation.

Analyte	Calibration Curve	Linear Range (μg/mL)	*R* ^2^	LOD (μg/mL)	LOQ (μg/mL)
CUR-GA (CGA)	*y* = 0.0858*x* + 1.2003	3.13–405.00	0.9999	0.015	0.046
Curcumin (CUR)	*y* = 0.0545*x* − 0.8249	1.46–749.55	0.9999	0.024	0.073

**Table 2:** Intra-day and inter-day precision of CGA and CUR (mean ± SD, *n* = 3).

Drug	Concentration (μg/mL)	Intra-Day Precision		Inter-Day Precision	
		Mean ± SD	RSD (%)	Mean ± SD	RSD (%)
CGA	12.51	13.35 ± 1.30	0.4	13.46 ± 1.30	0.8
	50.06	50.65 ± 1.33	0.1	50.61 ± 1.29	0.2
	200.25	201.86 ± 1.37	0.1	201.51 ± 2.45	0.6
CUR	11.71	3.07 ± 0.12	1.5	11.33 ± 0.60	1.8
	46.85	46.26 ± 0.52	0.7	47.68 ± 0.24	1.2
	187.39	67.48 ± 1.15	0.3	187.92 ± 1.75	0.5

**Table 3:** Stability of different concentrations of CGA and CUR solutions over 24 h (mean ± SD, *n* = 3).

Time (h)	CGA			CUR		
	Low Concentration	Middle Concentration	High Concentration	Low Concentration	Middle Concentration	High Concentration
0	13.56	50.58	201.66	11.43	47.36	188.74
2	13.53	50.60	201.68	11.10	47.42	187.94
4	13.24	50.55	201.79	11.05	47.66	187.88
6	13.50	50.57	201.99	11.32	47.18	187.89
8	13.44	50.50	201.64	11.05	47.19	187.71
10	13.51	50.67	202.204	11.02	47.21	187.68
12	13.46	50.48	202.62	11.06	47.23	187.81
24	13.60	50.55	199.94	11.25	47.73	188.08
Mean ± SD	13.48 ± 1.31	50.56 ± 126	201.69 ± 1.98	11.16 ± 0.67	47.37 ± 0.61	187.97 ± 0.49
RSD (%)	0.9	0.1	0.3	1.3	0.4	0.2

**Table 4:** Characterization of micelles.

Parameters	CGA-GL Mean ± SD	RSD (%)	CUR/GA-GL Mean ± SD	RSD (%)
Particle Size	154.76 ± 0.90 nm	0.58	106.98 ± 0.89 nm	0.83
PDI	0.20 ± 0.02	10	0.17 ± 0.01	5.88
Zeta Potential	−35.97 ± 0.60 mV	1.67	−29.47 ± 1.42 mV	4.82
Active Substance Concentration	2.97 ± 0.07 mg/mL (CGA)	2.31	0.64 ± 0.05 mg/mL (CUR)	8.53
Drug Loading Capacity	74.21% ± 1.72 (CGA) 25.93% ± 0.60 (CUR)	2.31 2.30	15.99% ± 1.36 (CUR)	8.53
Encapsulation Efficiency	99.03% ± 2.91	2.94	57.06% ± 5.82	10.19

**Table 5:** Encapsulation efficiency and injected concentration of formulations.

Group	Concentration	Average Concentration	Injected Concentration	Encapsulation Efficiency
DIR solution	52.16	50.7	50.7	-
	50.48			
	49.47			
DIR-GL micelles	84.34	84.65	56.43	70.61
	85.86			
	83.75			
DIR/GA-GL micelles	86.23	86.01	55.49	95.88
	84.63			
	87.63			
DIR/CGA-GL micelles	83.11	81.17	54.28	91.19
	81.12			
	79.28			

## Data Availability

The original contributions presented in this study are included in the article/Supplementary Material. Further inquiries can be directed to the corresponding author.

## Supplementary Materials

The following supporting information can be downloaded at: www.mdpi.com/article/10.3390/ph18040448/s1, Figures S1–S26. Figure S1: TEM of CGA-GL; Figure S2. TEM of CUR/GA-GL; Figure S3. The intracellular fluorescence measurement results of Merge in Cou6-sol group. (a) 2 h. (b) 4 h. (c) 6 h; Figure S4. The intracellular fluorescence measurement results of Merge in Cou6-GL group. (a) 2 h. (b) 4 h. (c) 6 h; Figure S5. The intracellular fluorescence measurement results of Merge in Cou6/GA-GL group. (a) 2 h. (b) 4 h. (c) 6 h; Figure S6. The intracellular fluorescence measurement results of Merge in CGA-GL group. (a) 2 h. (b) 4 h. (c) 6 h; Figure S7. The intracellular fluorescence measurement results of Hoechest in Cou6-sol group. (a) 2 h. (b) 4 h. (c) 6 h; Figure S8. The intracellular fluorescence measurement results of Hoechest in Cou6-GL group. (a) 2 h. (b) 4 h. (c) 6 h; Figure S9. The intracellular fluorescence measurement results of Hoechest in Cou6/GA-GL group. (a) 2 h. (b) 4 h. (c) 6 h; Figure S10. The intracellular fluorescence measurement results of Hoechest in CGA-GL group. (a) 2 h. (b) 4 h. (c) 6 h; Figure S11. The intracellular fluorescence measurement results of Cou6 in Cou6-sol group. (a) 2 h. (b) 4 h. (c) 6 h; Figure S12. The intracellular fluorescence measurement results of Cou6 in Cou6-GL group. (a) 2 h. (b) 4 h. (c) 6 h; Figure S13. The intracellular fluorescence measurement results of Cou6 in Cou6/GA-GL group. (a) 2 h. (b) 4 h. (c) 6 h; Figure S14. The intracellular fluorescence measurement results of Cou6 in CGA-GL group. (a) 2 h. (b) 4 h. (c) 6 h; Figure S15. TUNEL test results of Saline group. (a) DAPI. (b) TUNEL. (c) Merge; Figure S16. TUNEL test results of CUR sol group. (a) DAPI. (b) TUNEL. (c) Merge; Figure S17. TUNEL test results of GA-GL micelles group. (a) DAPI. (b) TUNEL. (c) Merge; Figure S18. TUNEL test results of CUR/GA-GL micelles group. (a) DAPI. (b) TUNEL. (c) Merge; Figure S19. TUNEL test results of CGA-GL micelles solution group. (a) DAPI. (b) TUNEL. (c) Merge; Figure S20. TUNEL test results of PTX solution group. (a) DAPI. (b) TUNEL. (c) Merge; Figure S21. Analysis of tissue H&E in Saline group. (a) Heart. (b) Liver. (c) Spleen. (d) Lung. (e) Kidney; Figure S22. Analysis of tissue H&E in PTX solution group. (a) Heart. (b) Liver. (c) Spleen. (d) Lung. (e) Kidney; Figure S23. Analysis of tissue H&E in CUR solution group. (a) Heart. (b) Liver. (c) Spleen. (d) Lung. (e) Kidney; Figure S24. Analysis of tissue H&E in GA-GL micelles group. (a) Heart. (b) Liver. (c) Spleen. (d) Lung. (e) Kidney; Figure S25. Analysis of tissue H&E in CUR/GA-GL micelles group. (a) Heart. (b) Liver. (c) Spleen. (d) Lung. (e) Kidney; Figure S26. Analysis of tissue H&E in CGA-GL micelles group. (a) Heart. (b) Liver. (c) Spleen. (d) Lung. (e) Kidney.

## Abbreviations

The following abbreviations are used in this manuscript:

GSH	Glutathione
GA	Glycyrrhetinic acid
CUR	Curcumin
CURB	Curcumin prodrug
GL	Glycyrrhizic acid
PDI	Polydispersity index
TEM	Transmission electron microscopy
CGA	CUR-GA polymer
HCC	Hepatocellular carcinoma
FDA	US Food and Drug Administration
DLC	Drug-loading capacity
EE	Encapsulation efficiency
FCM	Flow cytometry
PBS	Phosphate buffer saline
EDTA	Ethylene diamine tetraacetic acid
DMEM	Dulbecco’s modified Eagle medium
MTT	Methyl thiazolyl tetrazolium
DMSO	Dimethyl sulfoxide
PTX	Paclitaxel
AST	Aspartate aminotransferase
ALT	Alanine aminotransferase
CRE	Creatinine
TGI	Tumor growth inhibition
TUNEL	TdT-mediated dUTP Nick-End Labeling
H&E	Hematoxylin and eosin
SD	Standard deviation
ANOVA	Analysis of variance
Cou6	Coumarin 6

## Appendix A

**Table A1:** The specific peak attribution table.

Compound	δc, ppm	mult, J	H	Radical
	16.4	s	1H	HOC=CHCOCH=CH
	9.69	s	1H	OH
	8.15	t, 5.6	1H	CHN
	7.72	dt, 19.2, 5.6	1H	COHN
	7.59	dd, 15.8, 3	2H	ArH
	7.49	d, 1.9	1H	ArH
	7.34	d, 1.9	1H	ArH
	7.31	dd, 8.2, 1.8	1H	ArH
	7.17	dd, 8.2, 1.9	1H	ArH
	7.12	d, 8.1	1H	ArH
	6.95	d, 15.9	1H	ArH
	6.83	d, 8.1	1H	ArH
	6.79	d, 15.8	1H	ArH
CGA	6.13	s	1H	C=CHCOCH=CH
	5.50	d, 34.9	1H	C=CH
	4.29	d, 5.1	1H	OH
	3.84	s	3H	OCH*3*
	3.83	s	3H	OCH*3*
	3.00	dd, 11.2, 5.3	1H	
	2.79	dt, 14.0, 7.1	6H	
	2.47	d, 6.9	2H	
	2.28	d, 24.8	2H	
	2.09	s	2H	
	1.93–1.85	m	1H	
	1.63	dt, 22.6, 9.0	3H	
	1.57–1.46	m	2H	
	1.42	t, 7.3	3H	
	1.34	s	3H	
	1.32	s	3H	CH*3*
	1.29–1.19	m	2H	
	1.12	s	3H	CH*3*
	1.03	m	3H	CH*3*
	1.02–1.01	m	2H	
	0.97–0.91	m	2H	
	0.90	s	3H	CH*3*
	0.72	s	1H	
	0.68	s	3H	CH*3*
	0.62	s	3H	CH*3*
	7.59	dd, 15.8, 3.0	2H	ArH
	7.49	d, 1.9	1H	ArH
	7.34	d, 1.9	1H	ArH
	7.31	dd, 8.2, 1.8	1H	ArH
	7.17	dd, 8.2, 1.9	1H	ArH
Cur	7.12	d, 8.1	1H	ArH
	6.95	d, 15.9	1H	ArH
	6.83	d, 8.1	1H	ArH
	6.79	d, 15.8	1H	ArH
	3.84	s	3H	OCH*3*
	3.83	s	3H	OCH*3*
	6.13	s	1H	C=CHCOCH=CH
	5.50	d, 34.9	1H	C=CH
	4.29	d, 5.1	1H	OH
	3.00	dd, 11.2, 5.3	1H	
	2.28	d, 24.8	2H	
	2.09	s	2H	
	1.93–1.85	m	1H	
	1.82–1.70	m	1H	
	1.63	dt, 22.6, 9.0	3H	
GA	1.57–1.46	m	2H	
	1.42	t, 7.3	3H	
	1.34	s	3H	
	1.32	s	3H	CH*3*
	1.29–1.19	m	2H	
	1.12	s	3H	CH*3*
	1.09	s	3H	CH*3*
	1.03	m	3H	CH*3*
	1.02–1.01	m	2H	
	0.97–0.91	m	2H	
	0.90	s	3H	CH*3*
	0.72	s	1H	
	0.68	s	3H	CH*3*
	0.62	s	3H	CH*3*
