# Supplementary material for: Preparation and Evaluation of Hepatoma-Targeting Glycyrrhetinic Acid Composite Micelles Loaded with Curcumin
Source: Pharmaceuticals (Basel). 2025 Mar 23;18(4):448. doi: 10.3390/ph18040448 (PMC12030034; doi:10.3390/ph18040448)
Supplement: Supplementary file 1 [file pharmaceuticals-18-00448-s001.zip › pharmaceuticals-3525505-supplementary.pdf]

supplementary materials

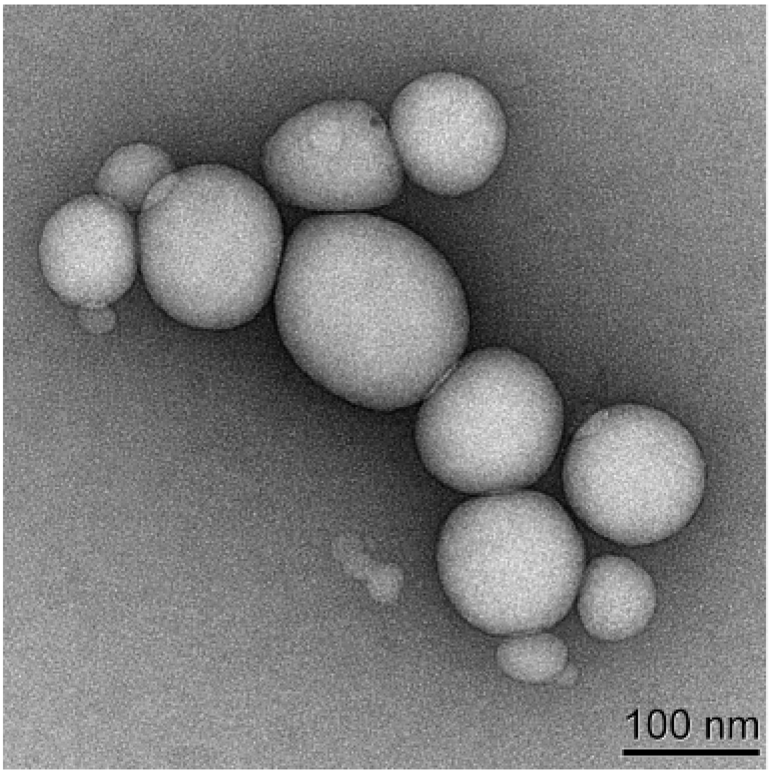

Figure S1. TEM of CGA-GL

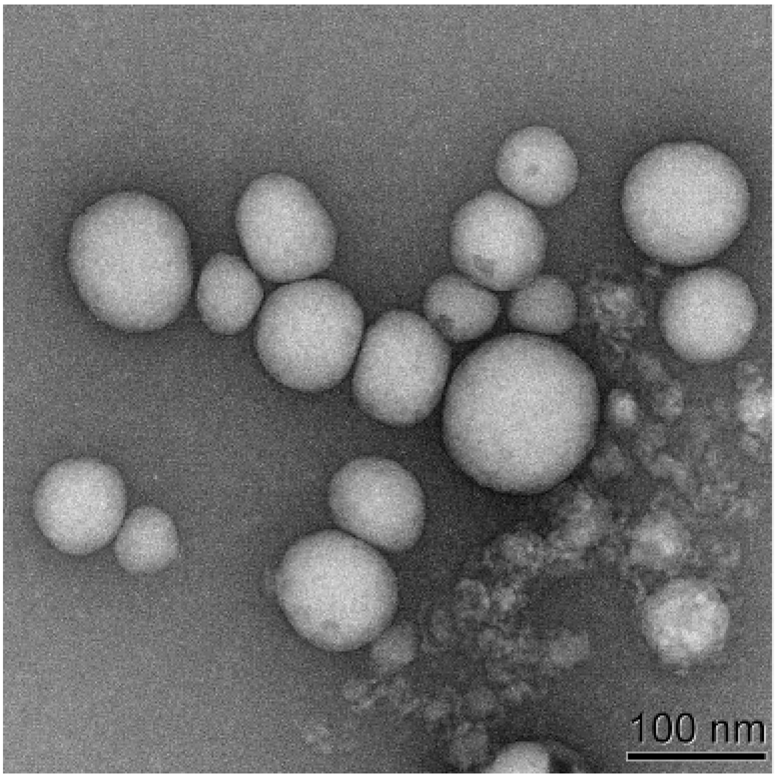

Figure S2. TEM of CUR/GA-GL

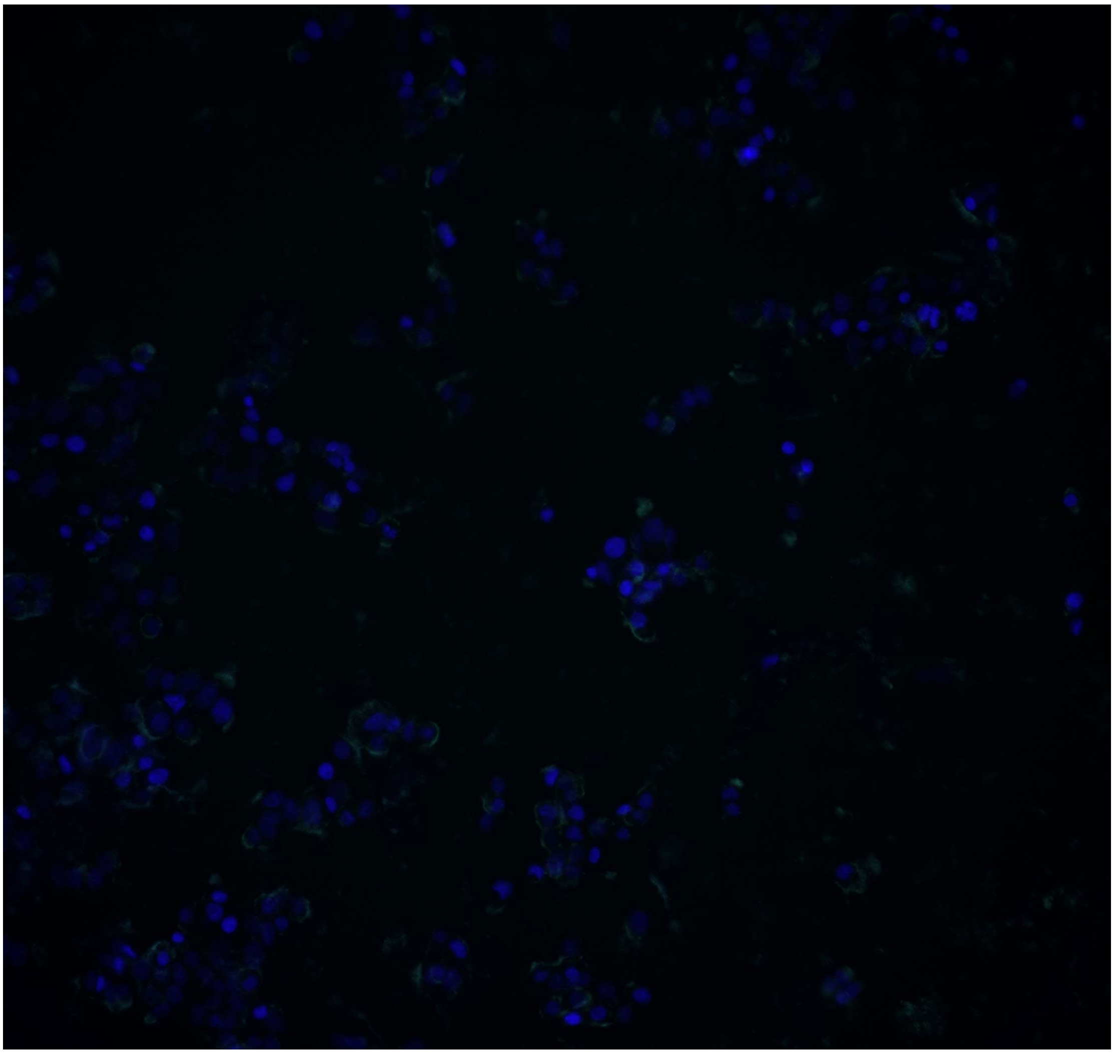

(a)

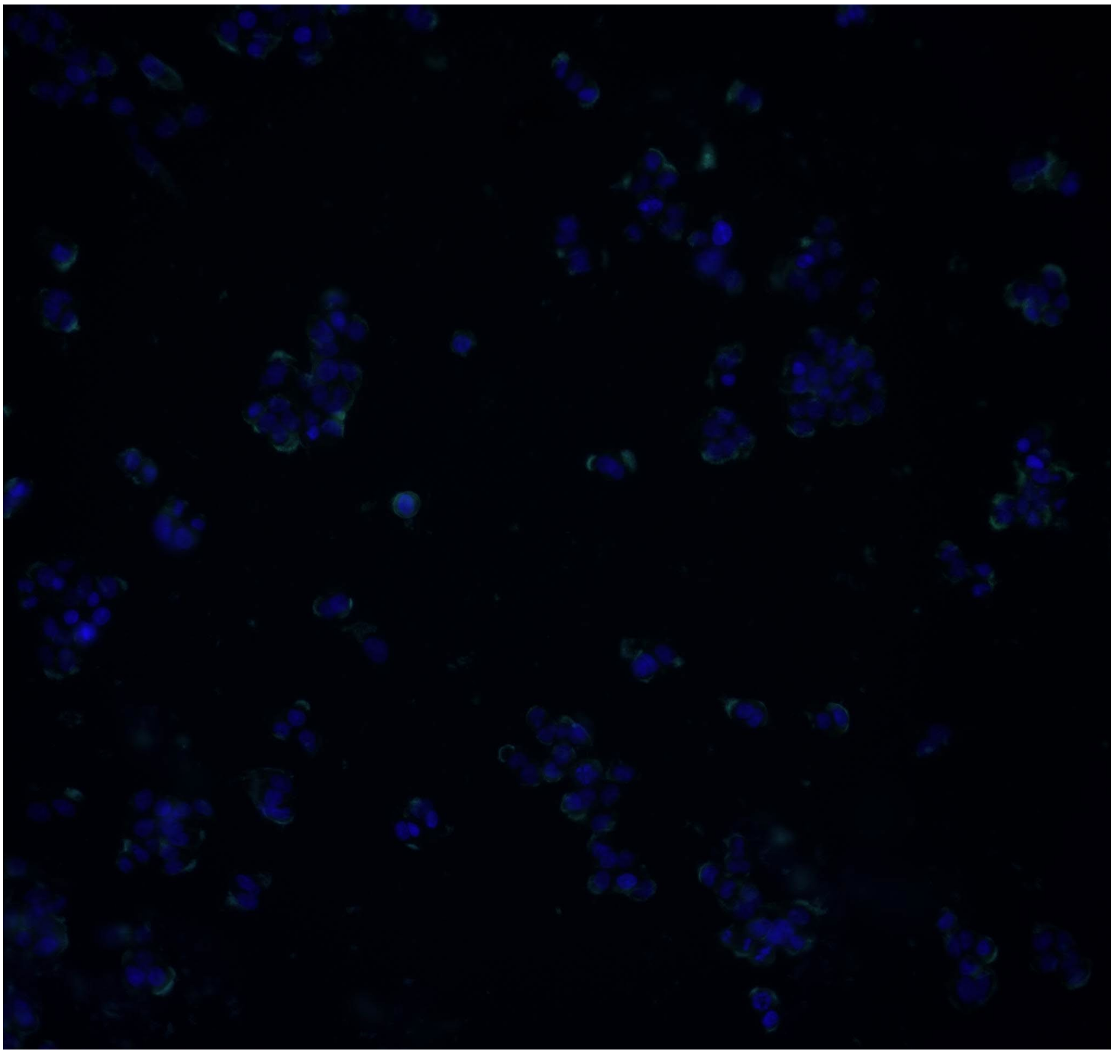

(b)

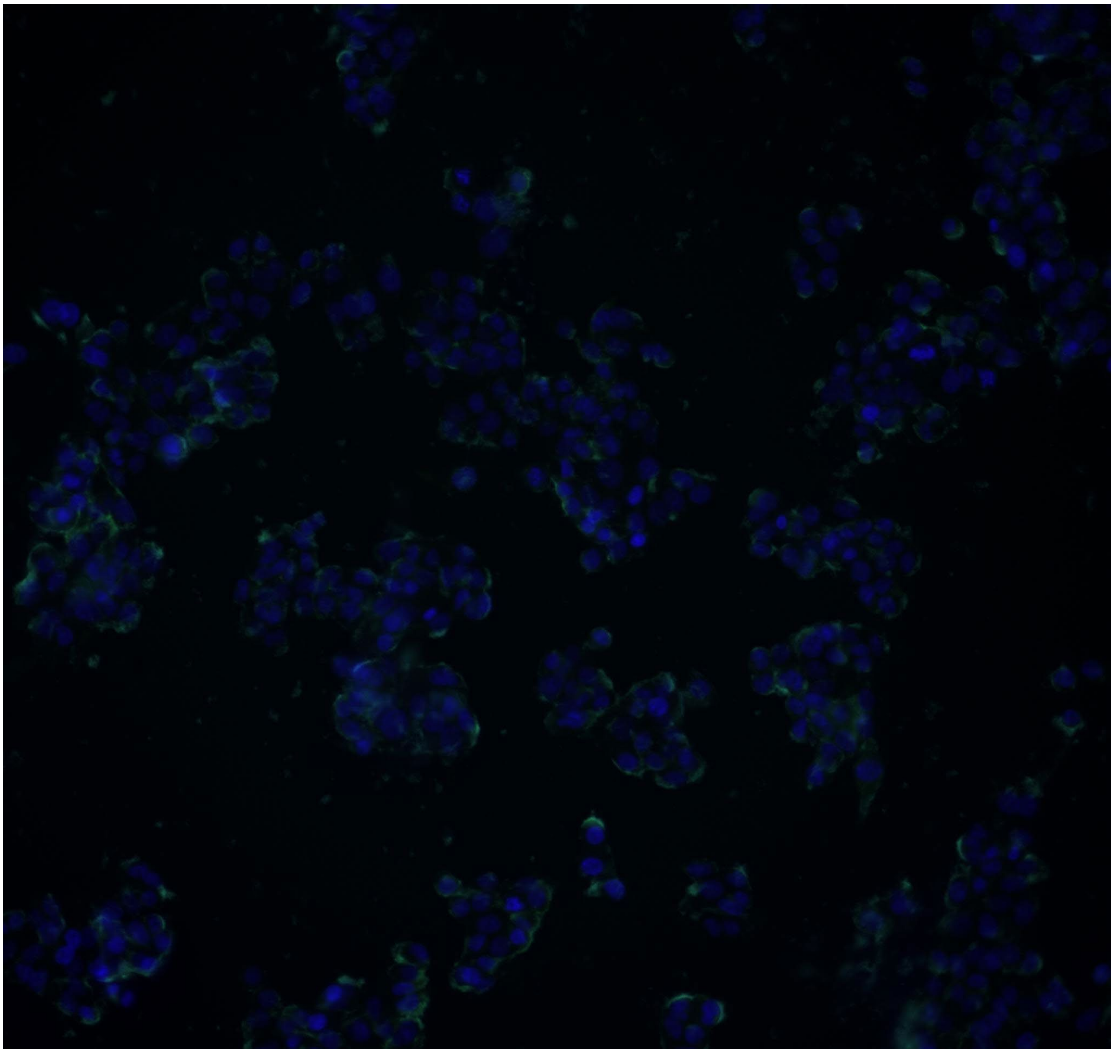

(c)

Figure S3. The intracellular fluorescence measurement results of Merge in Cou6-sol group. (a)2 h. (b)4 h. (c)6 h.

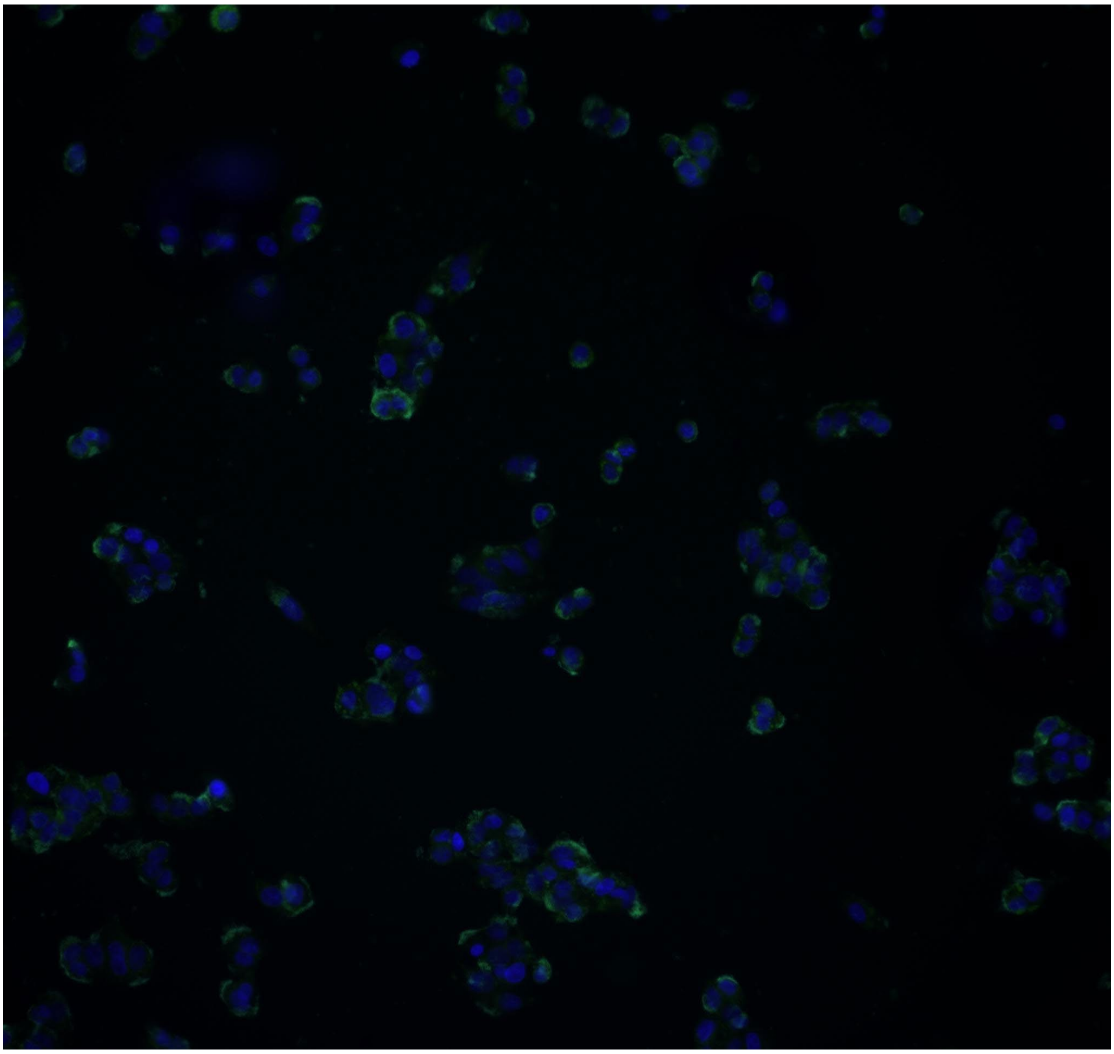

(a)

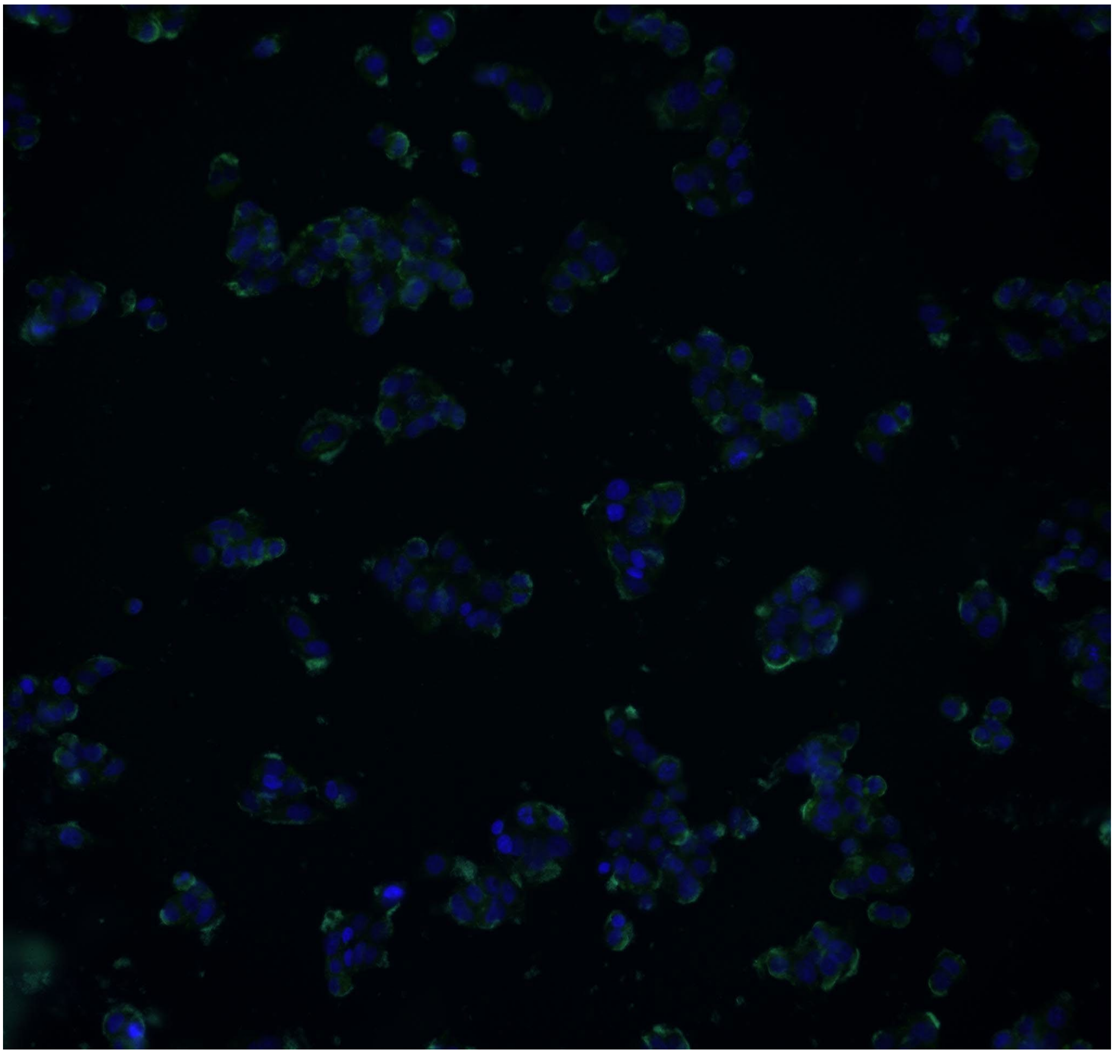

(b)

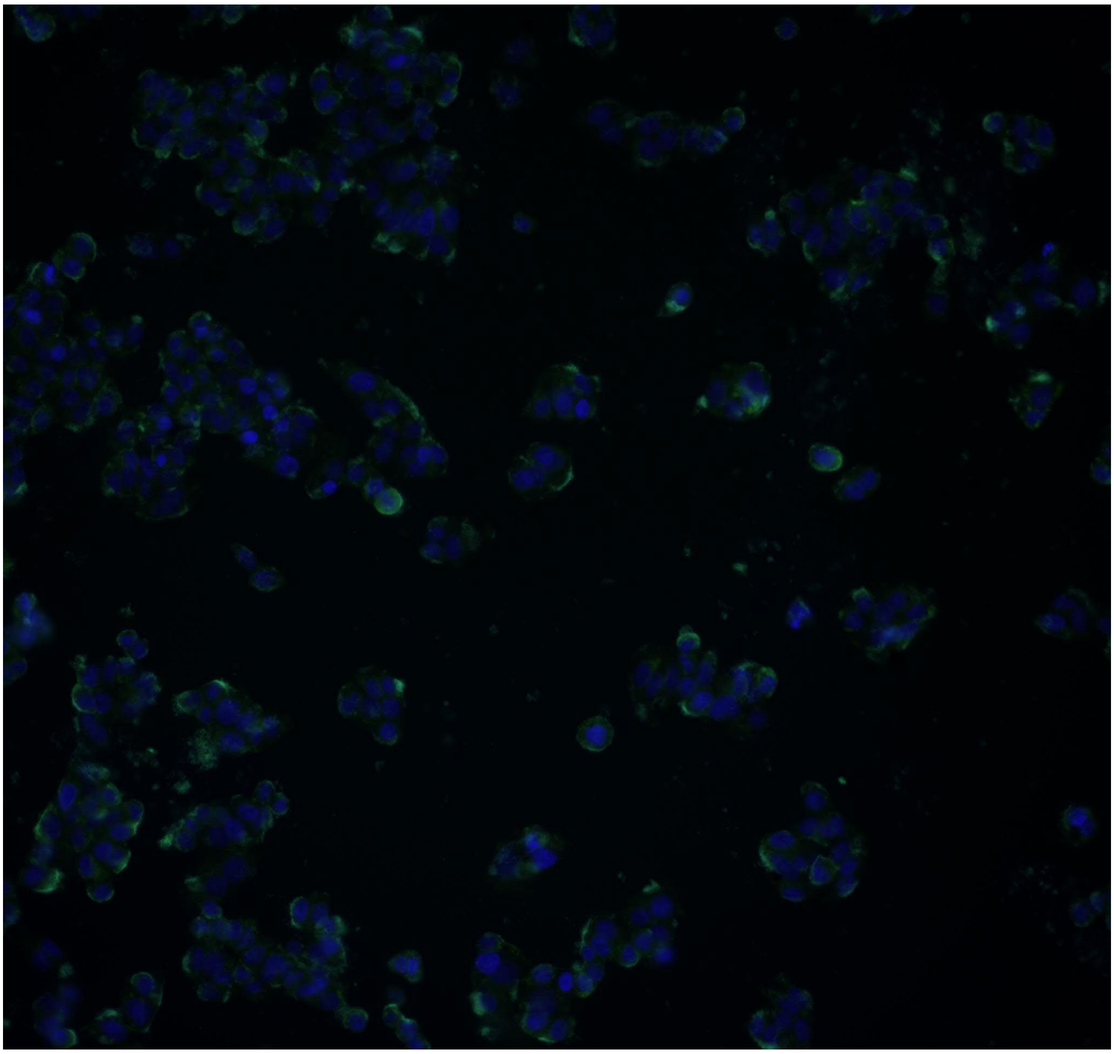

(c)

Figure S4. The intracellular fluorescence measurement results of Merge in Cou6-GL group. (a)2 h. (b)4 h. (c)6 h.

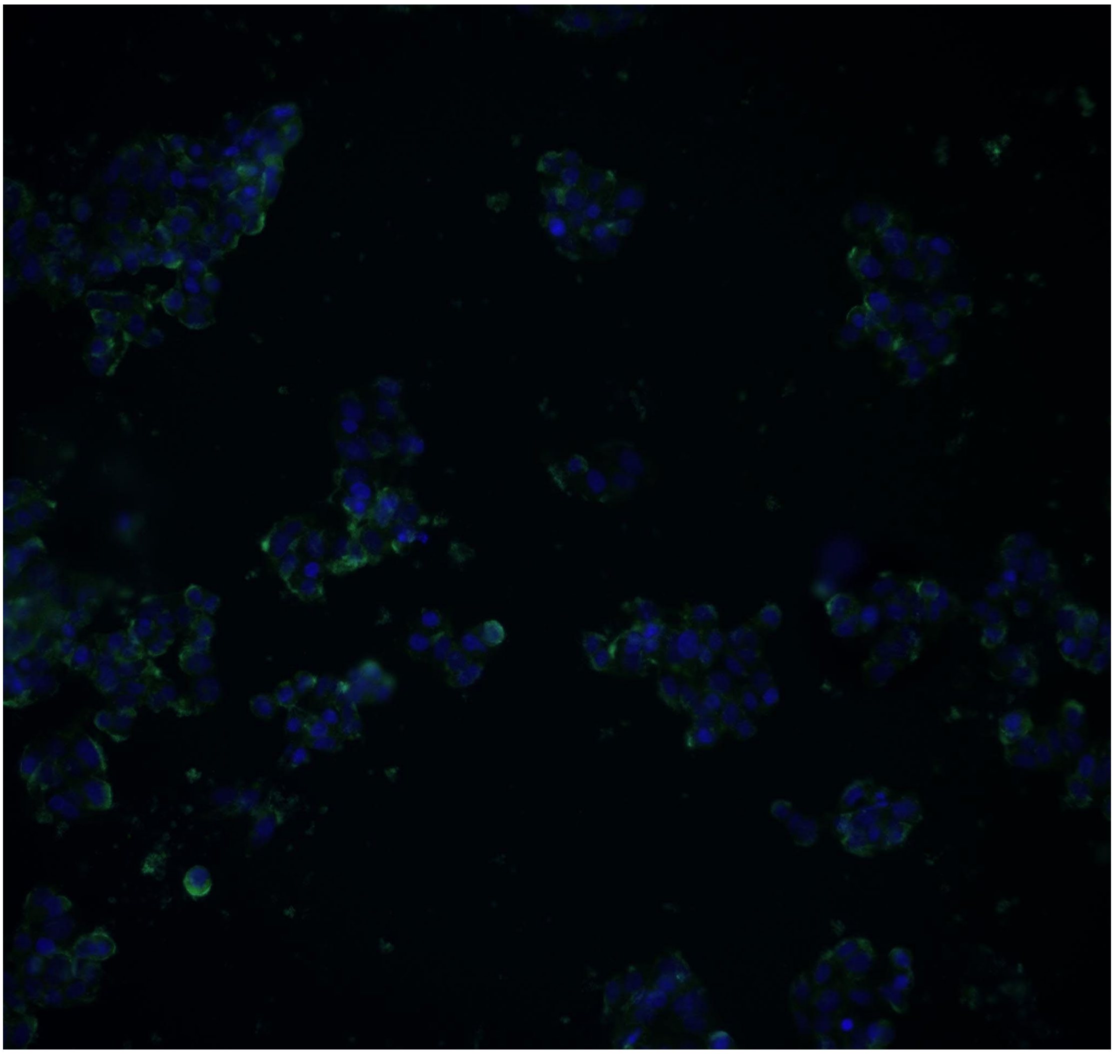

(a)

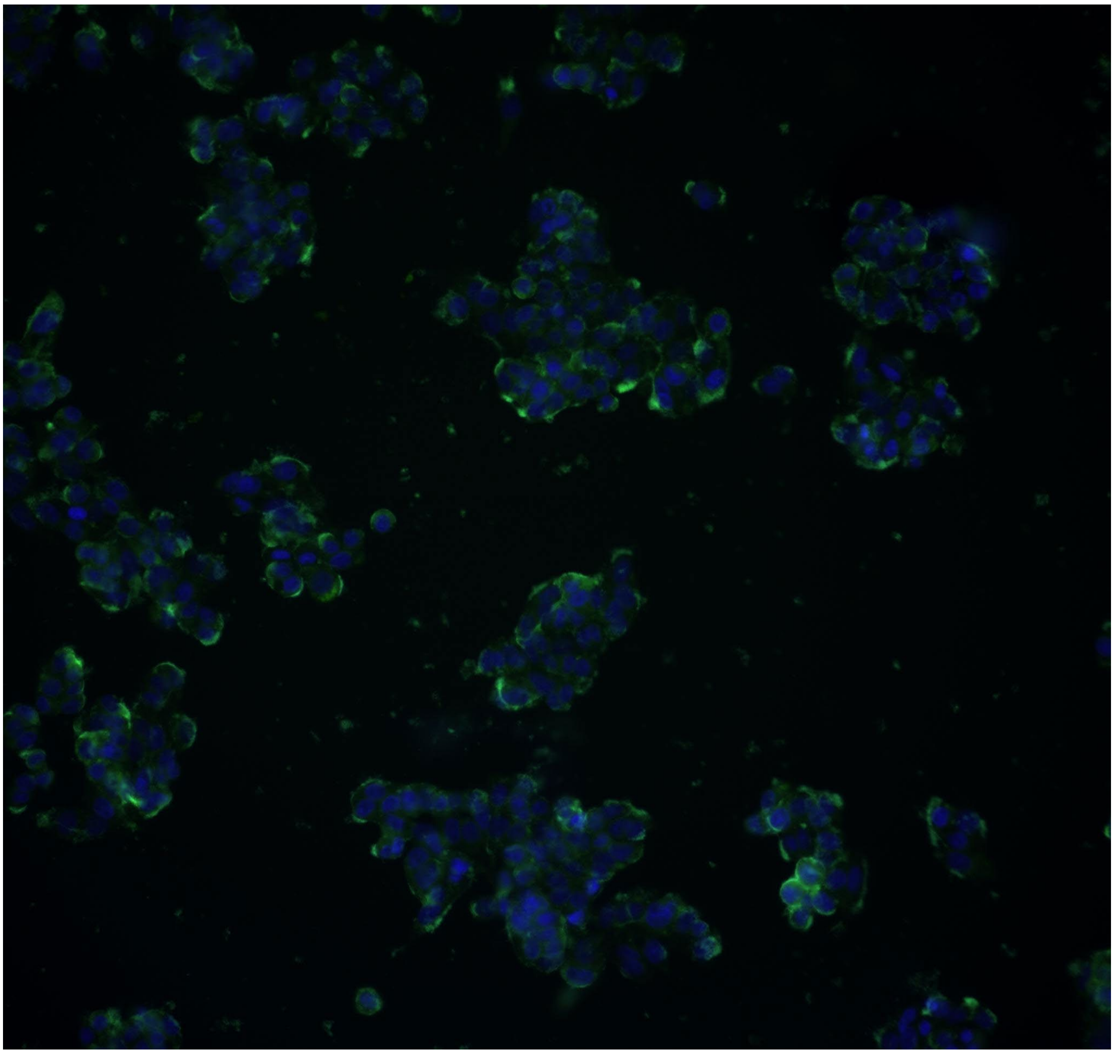

(b)

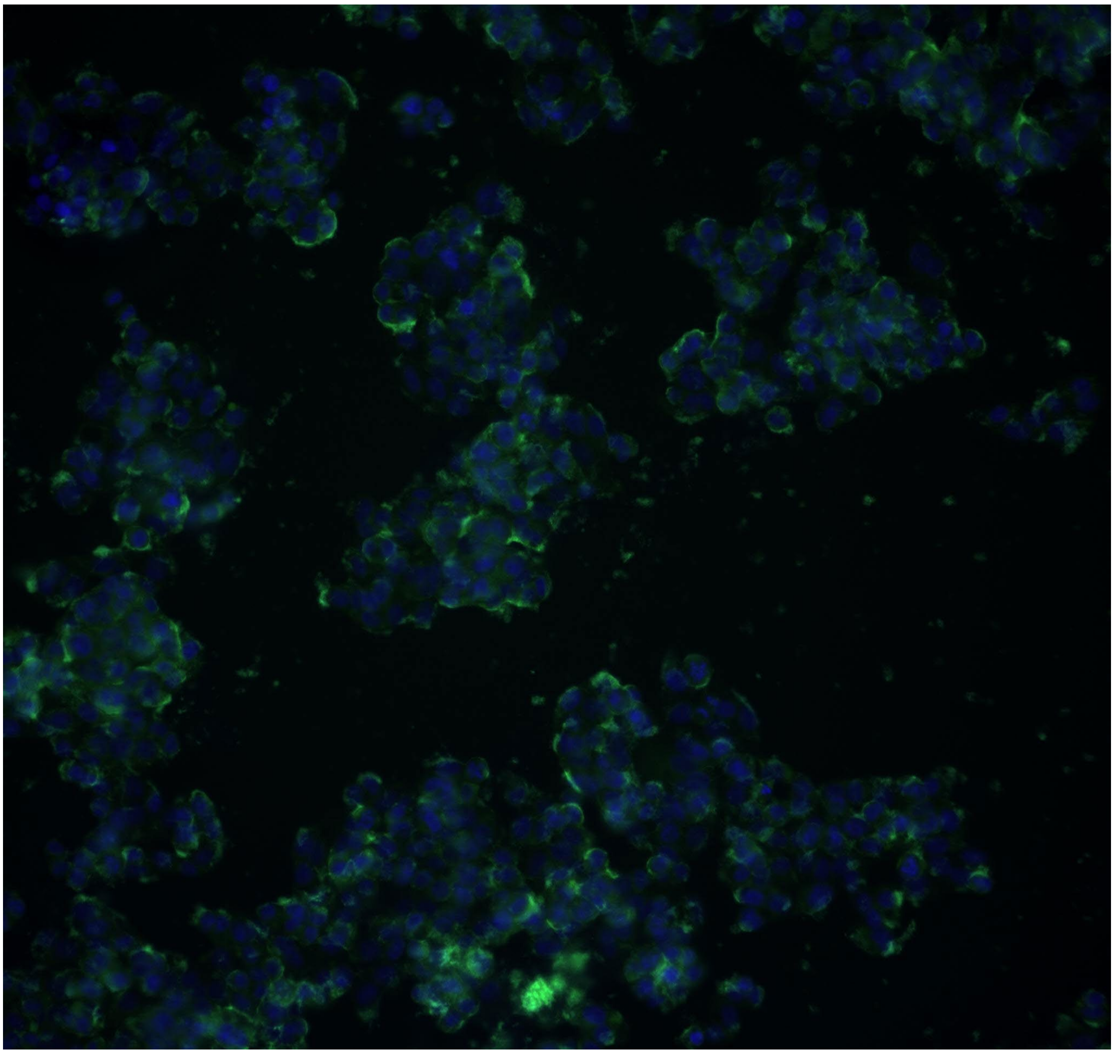

(c)

Figure S5. The intracellular fluorescence measurement results of Merge in Cou6/GA - GL group. (a)2 h. (b)4 h. (c)6 h.

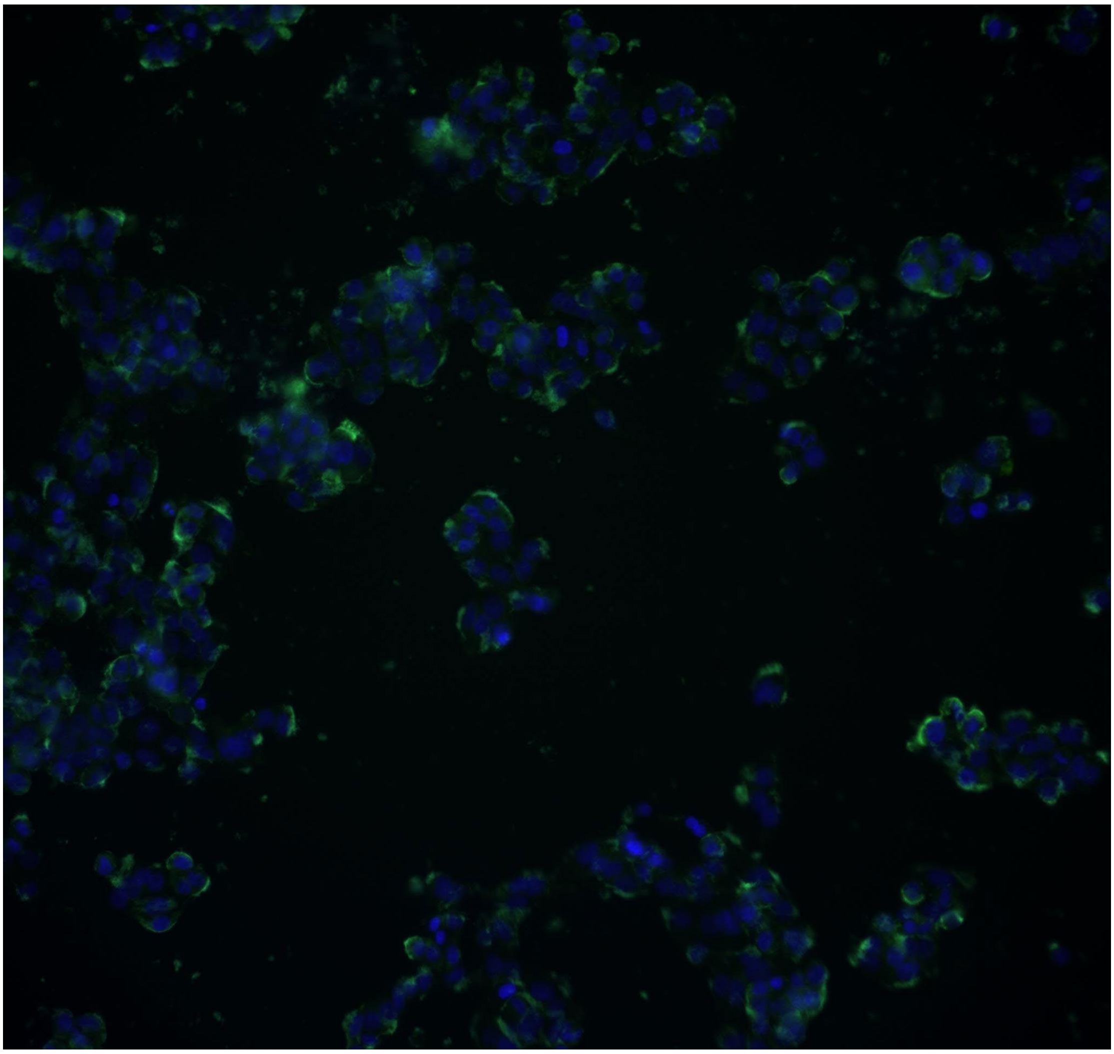

(a)

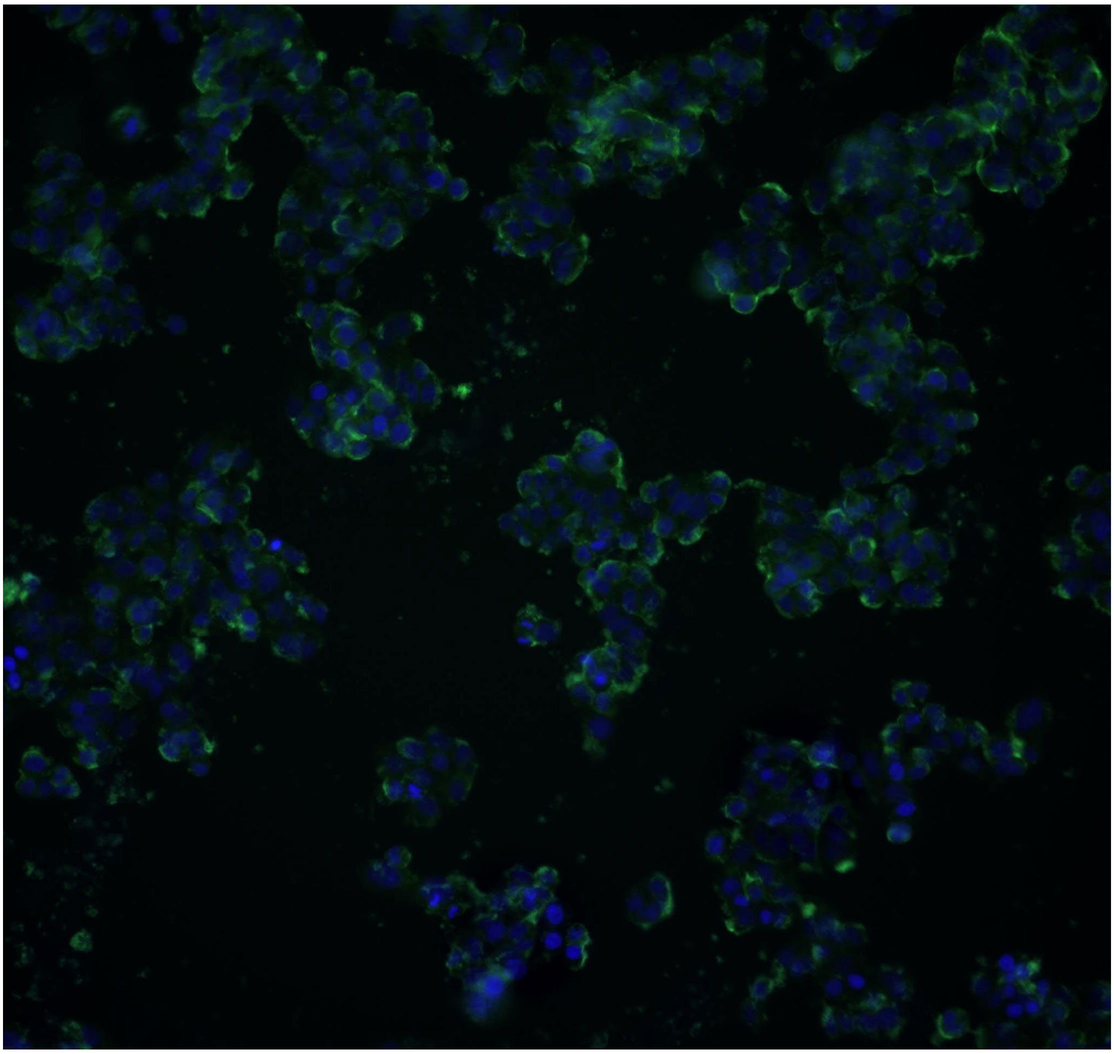

(b)

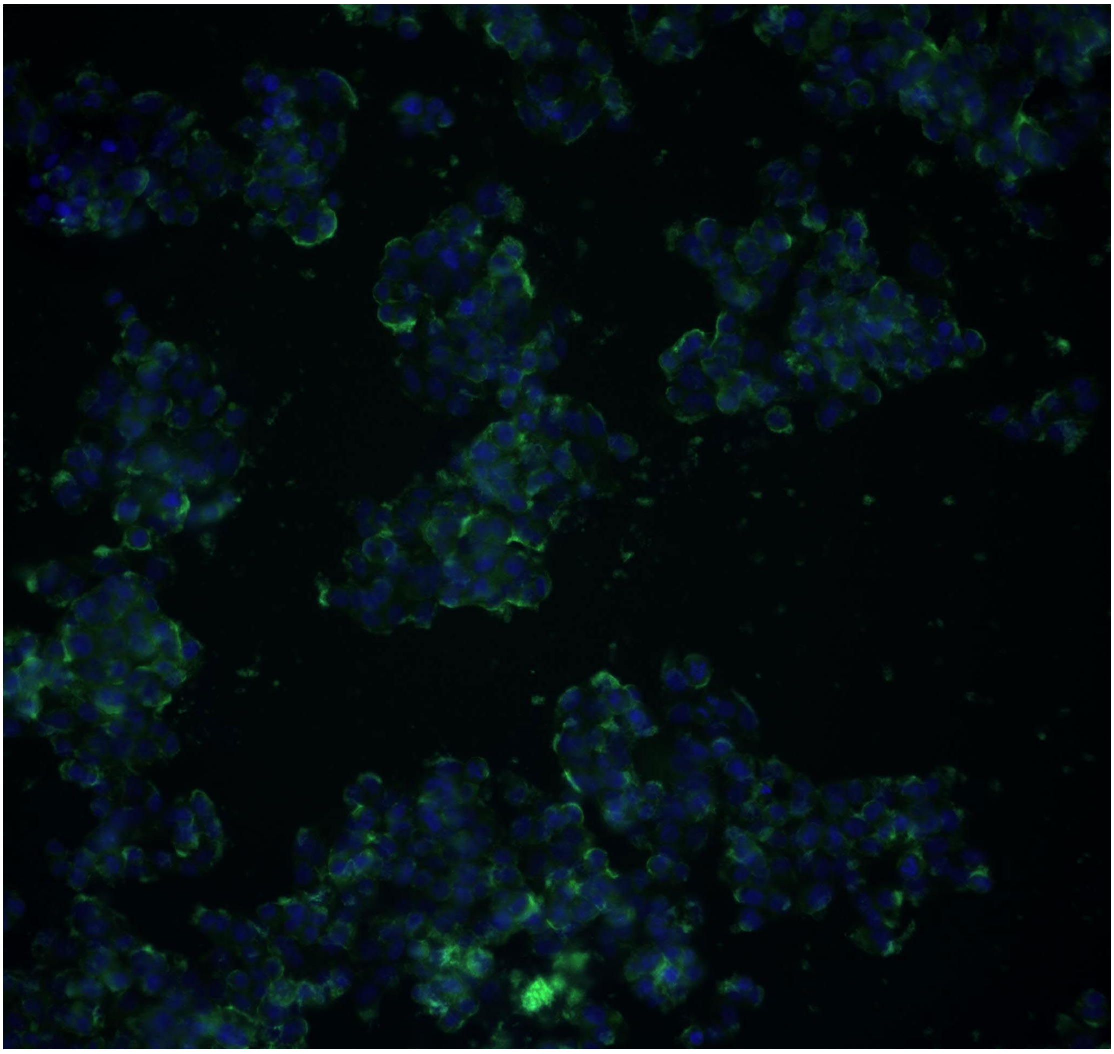

(c)

Figure S6. The intracellular fluorescence measurement results of Merge in CGA - GL group.
(a)2 h. (b)4 h. (c)6 h.

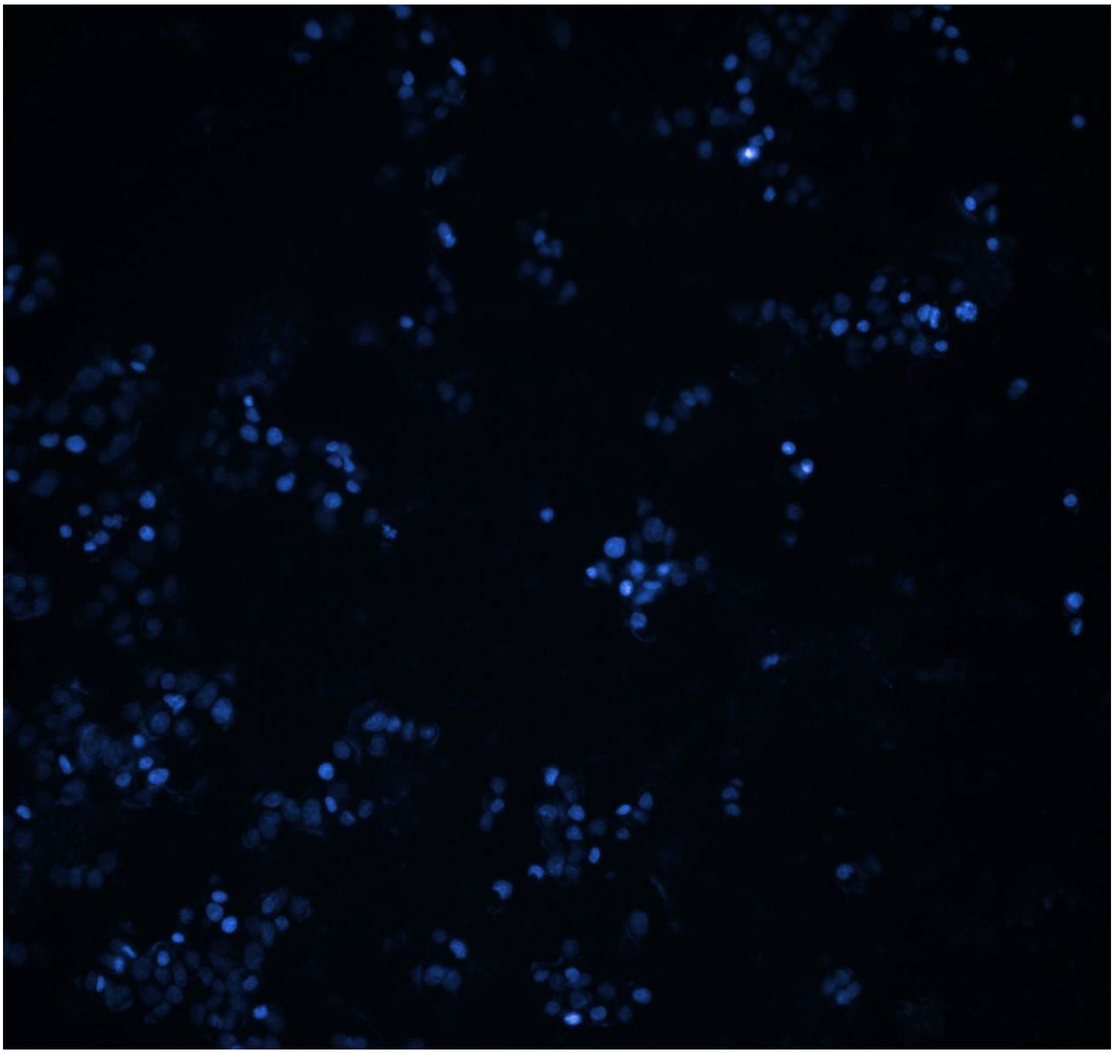

(a)

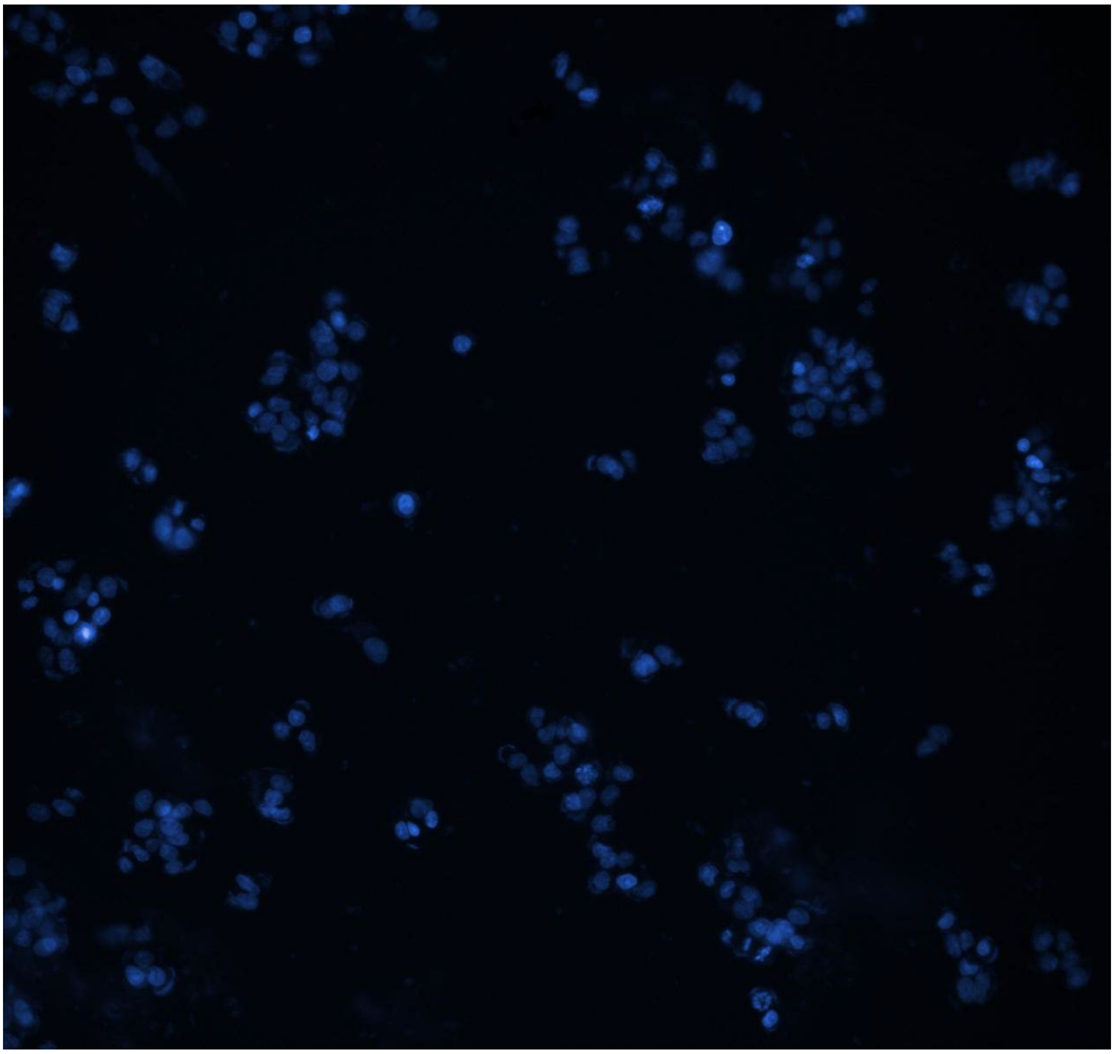

(b)

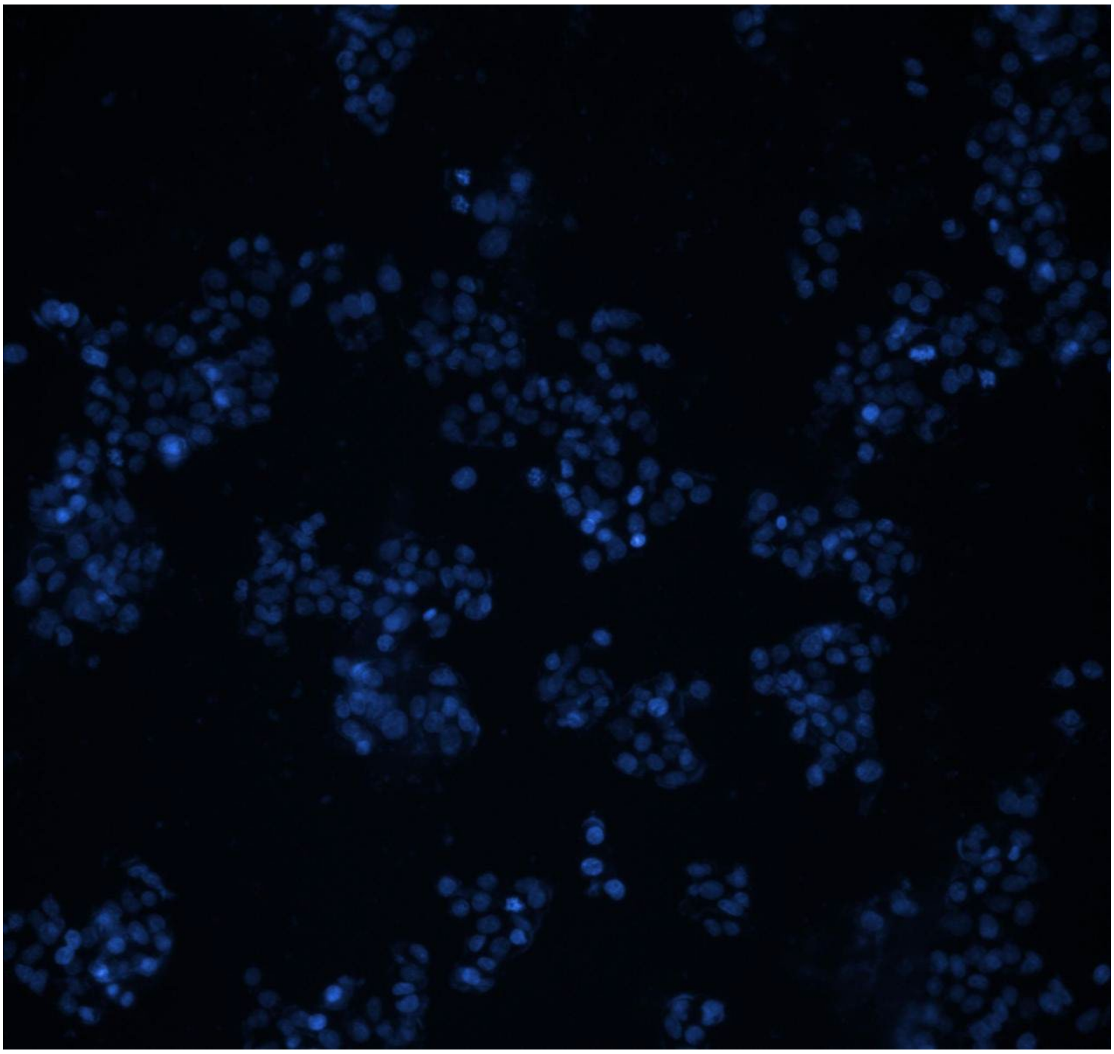

(c)

Figure S7. The intracellular fluorescence measurement results of Hoechest in Cou6-sol group. (a)2 h. (b)4 h. (c)6 h.

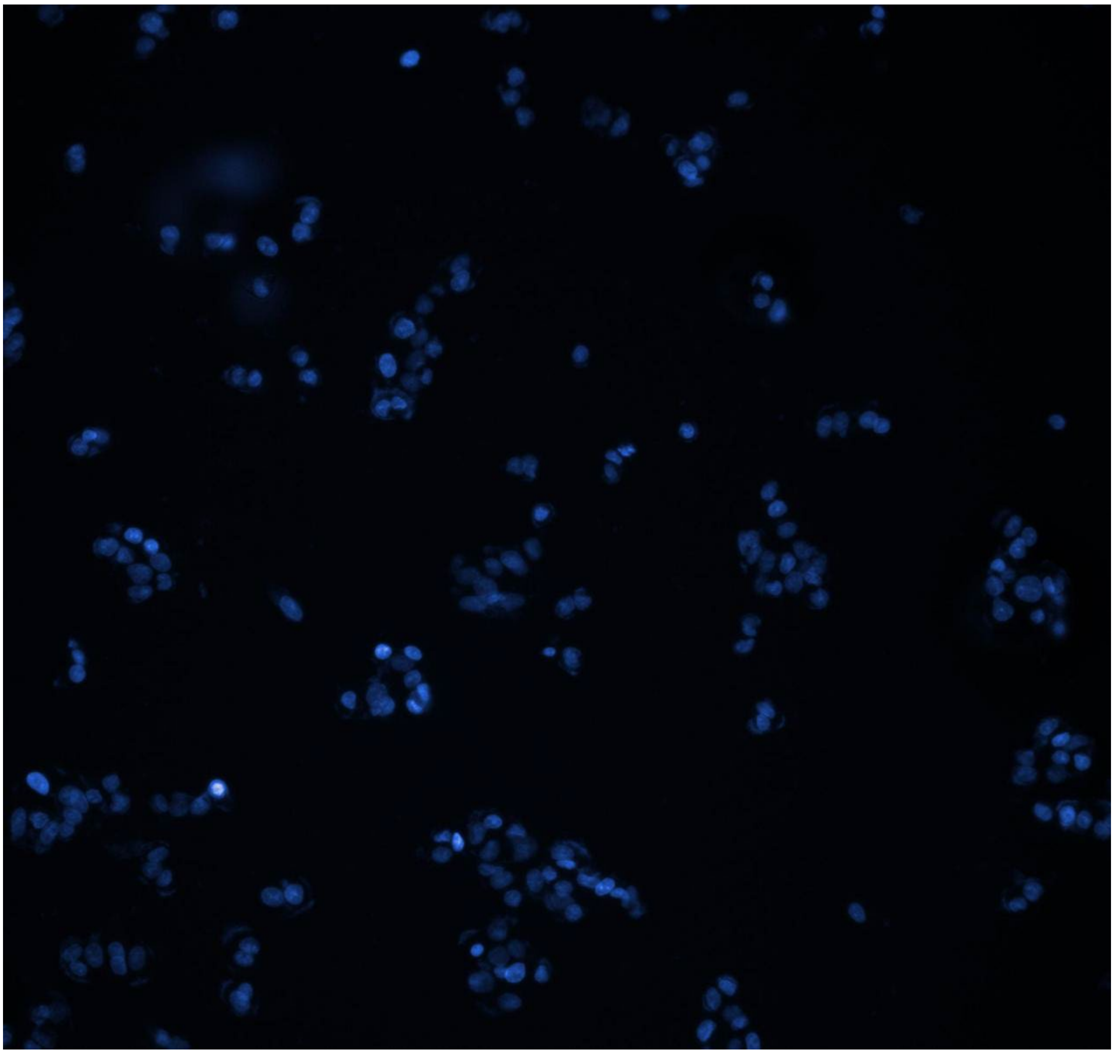

(a)

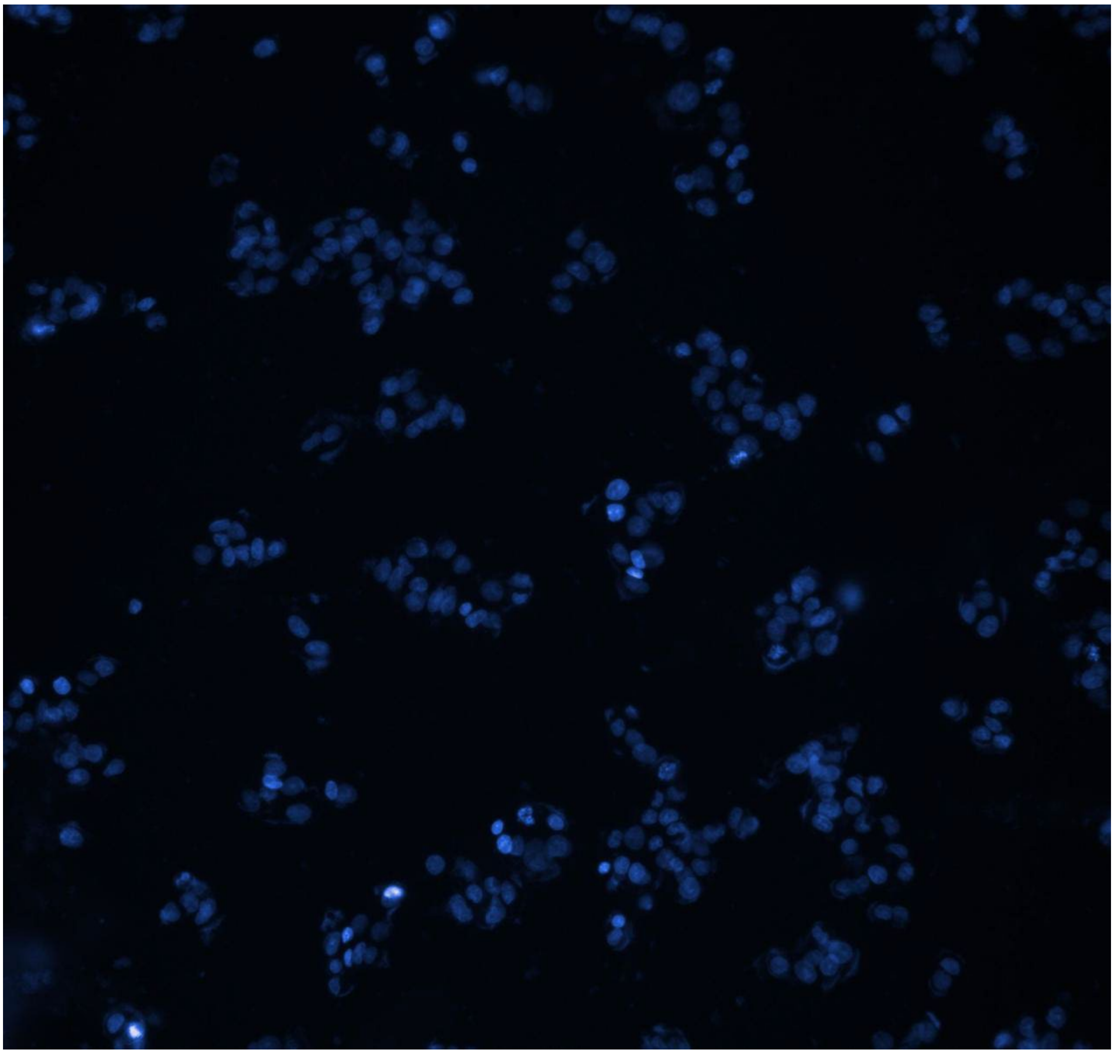

(b)

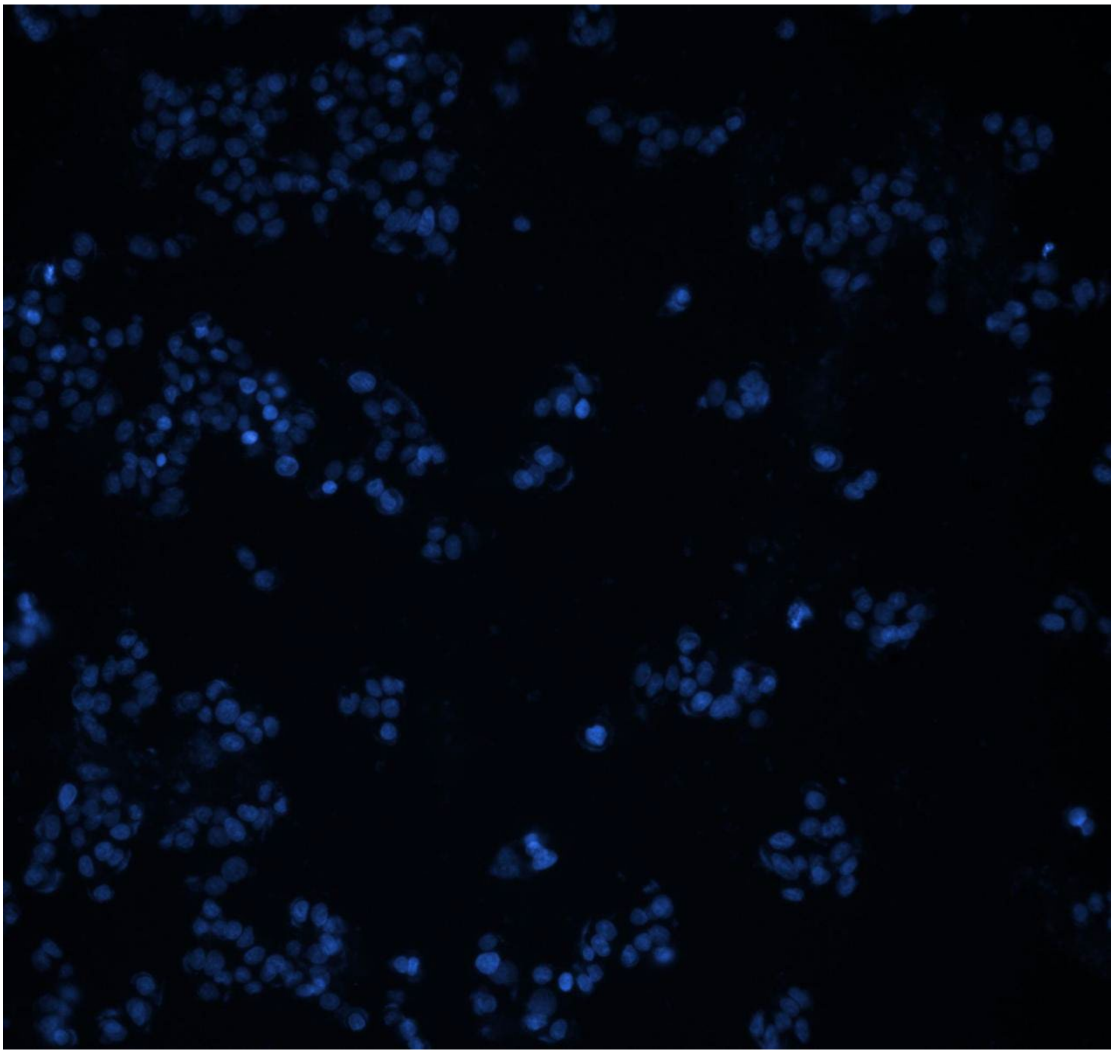

(c)

Figure S8. The intracellular fluorescence measurement results of Hoechest in Cou6-GL group. (a)2 h. (b)4 h. (c)6 h.

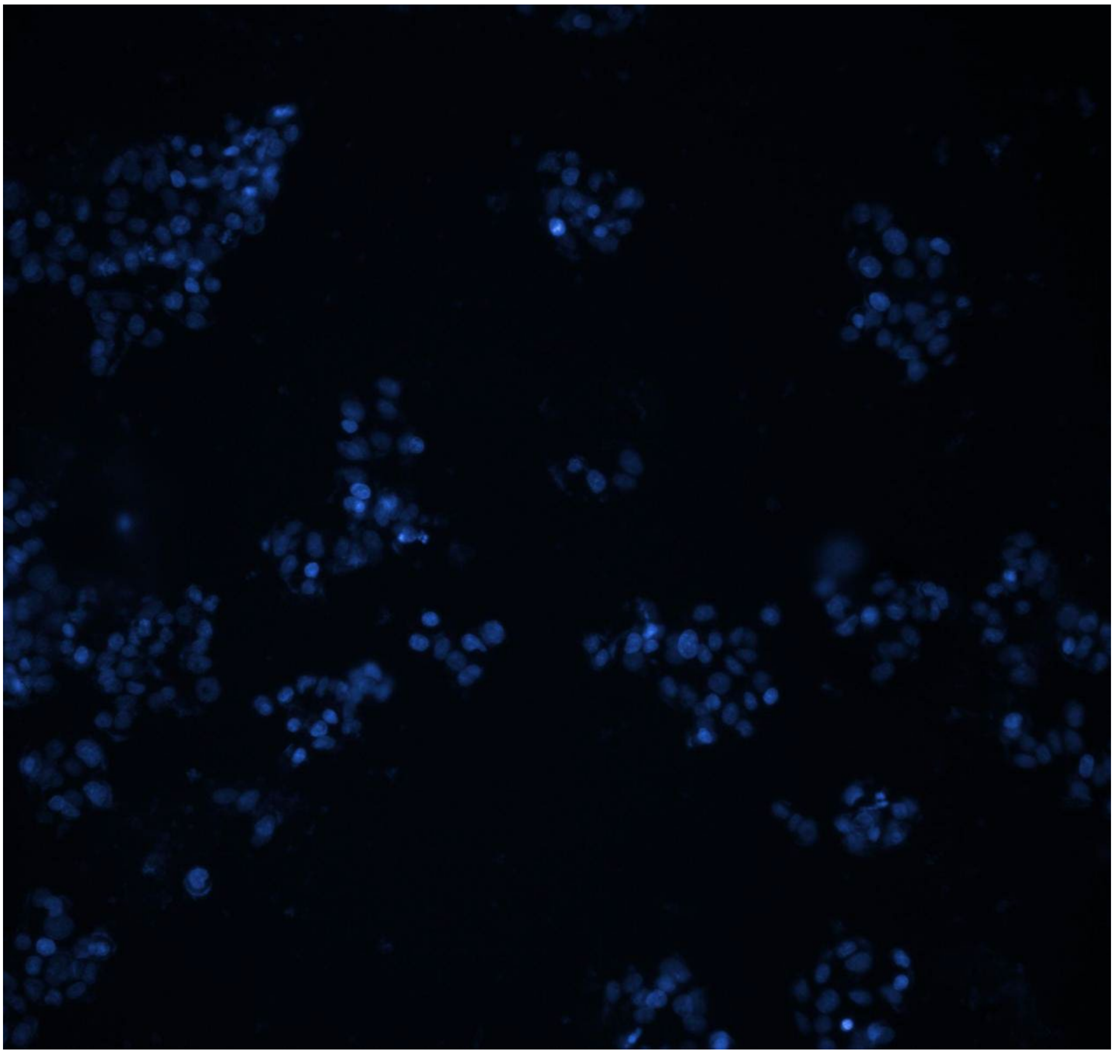

(a)

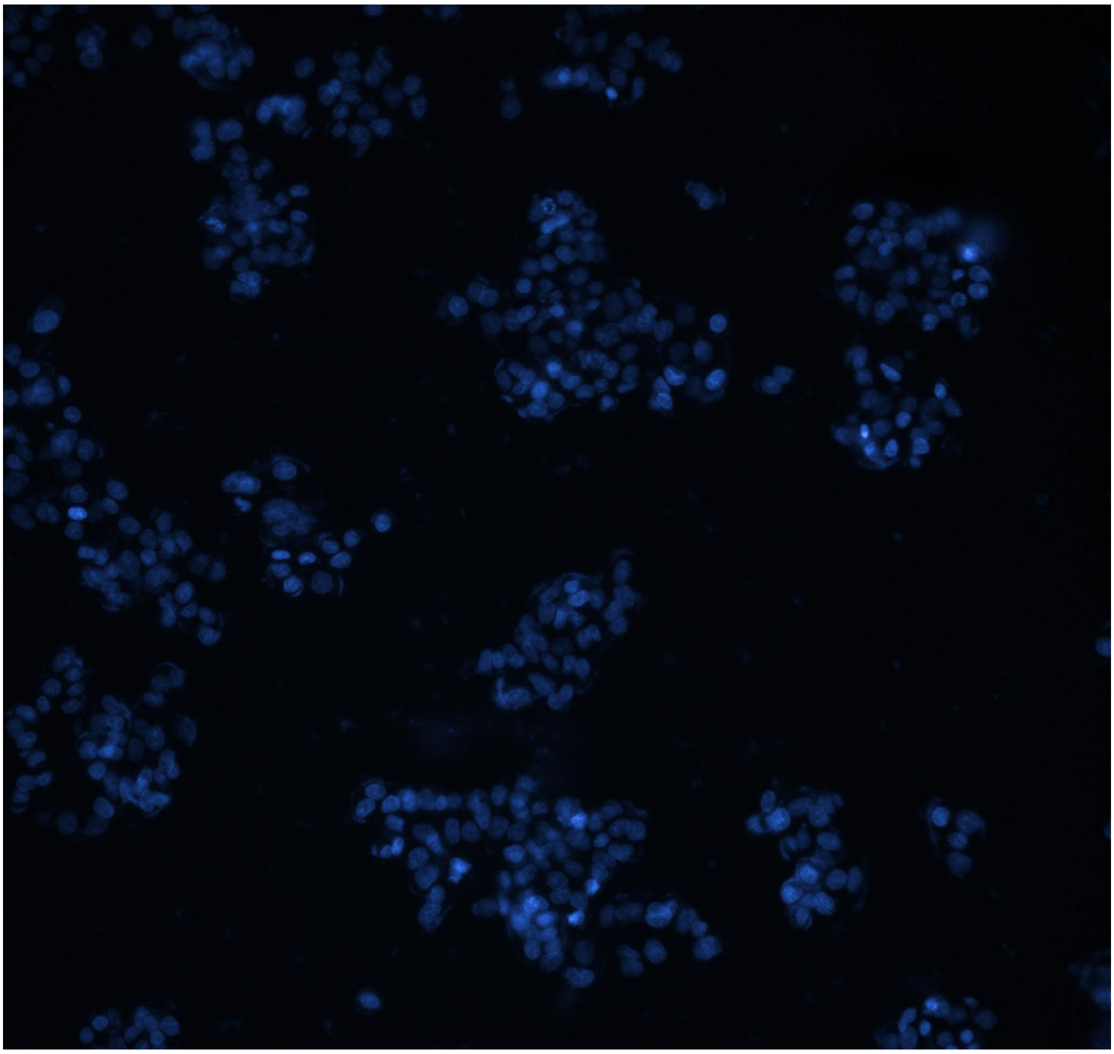

(b)

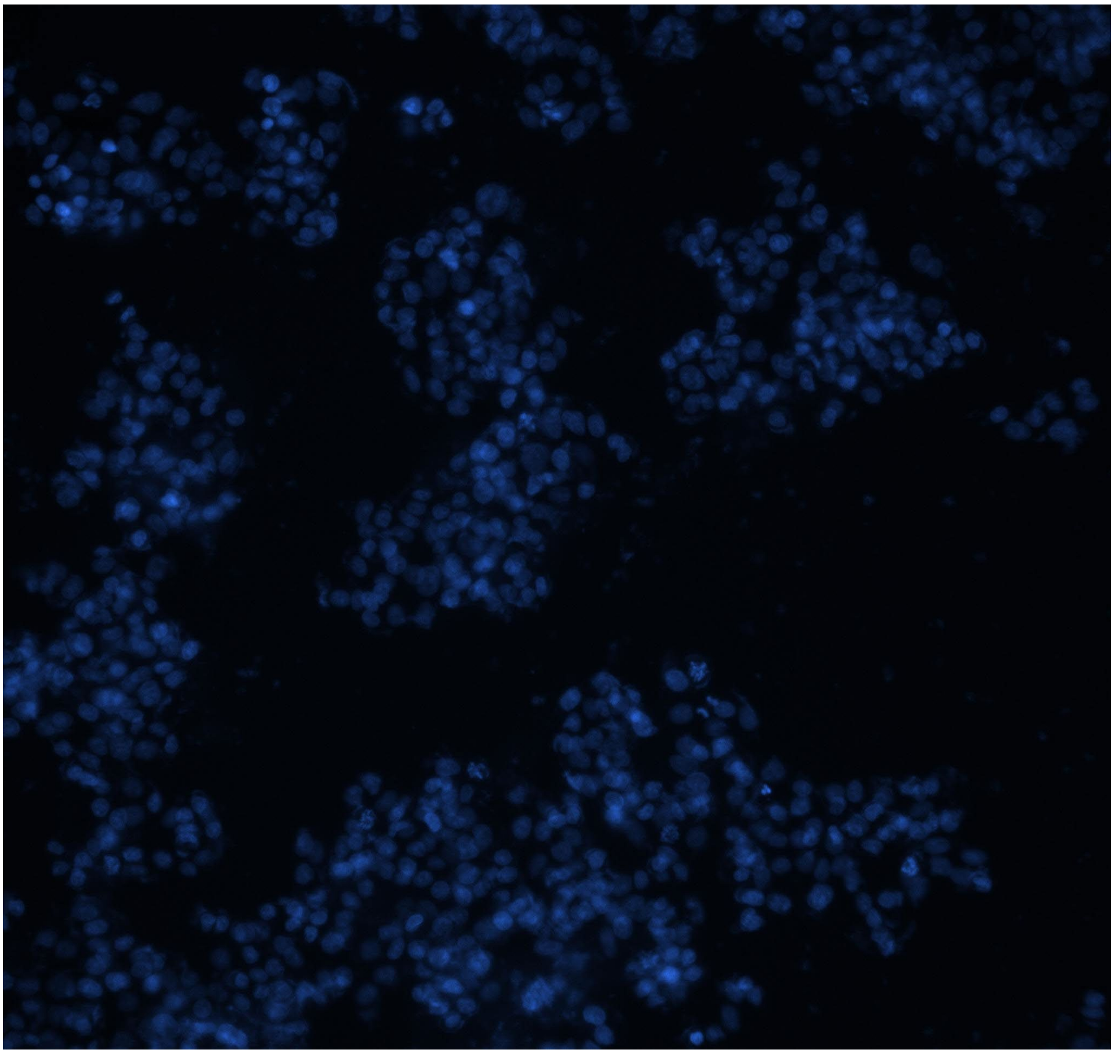

(c)

Figure S9. The intracellular fluorescence measurement results of Hoechest in Cou6/GA - GL group. (a)2 h. (b)4 h. (c)6 h.

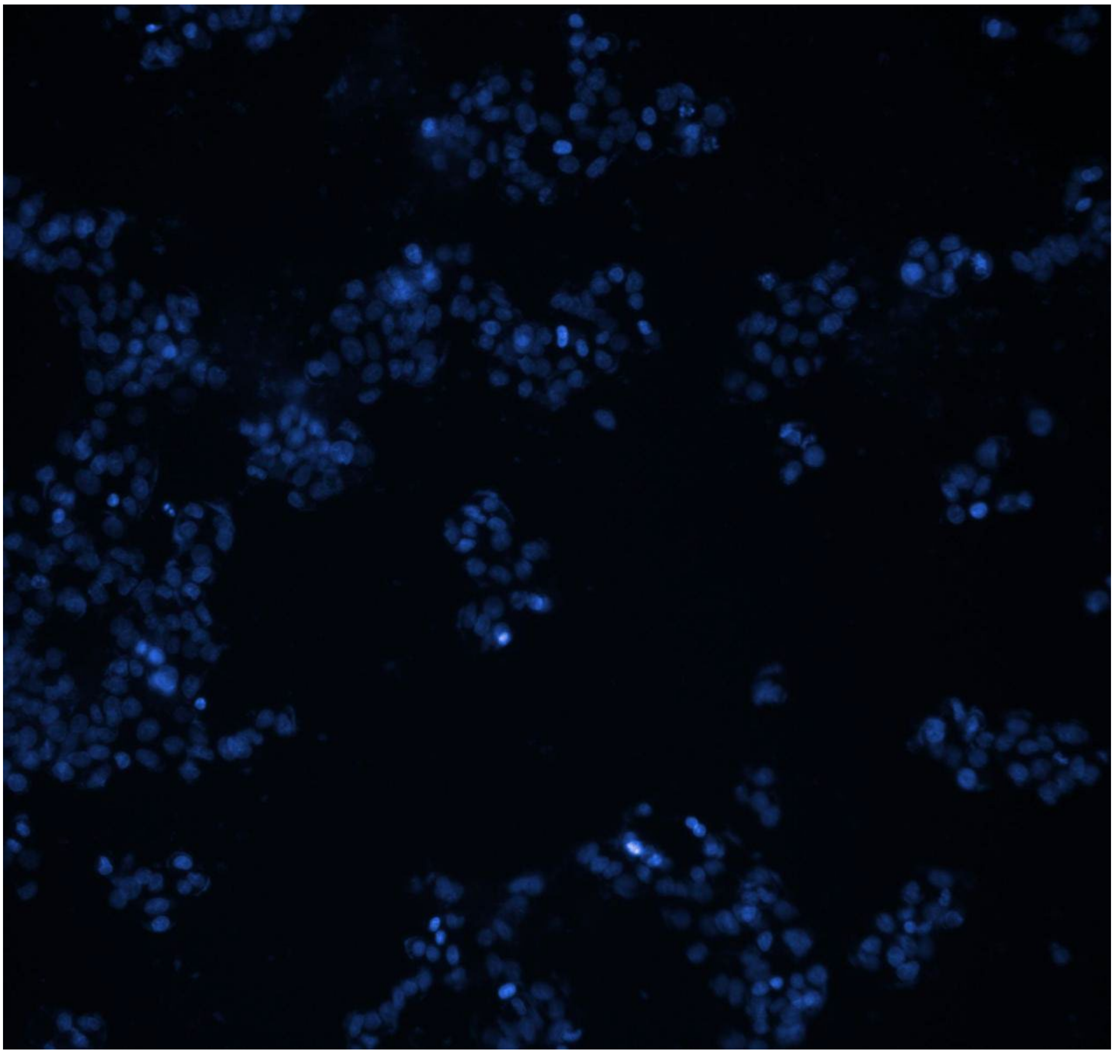

(a)

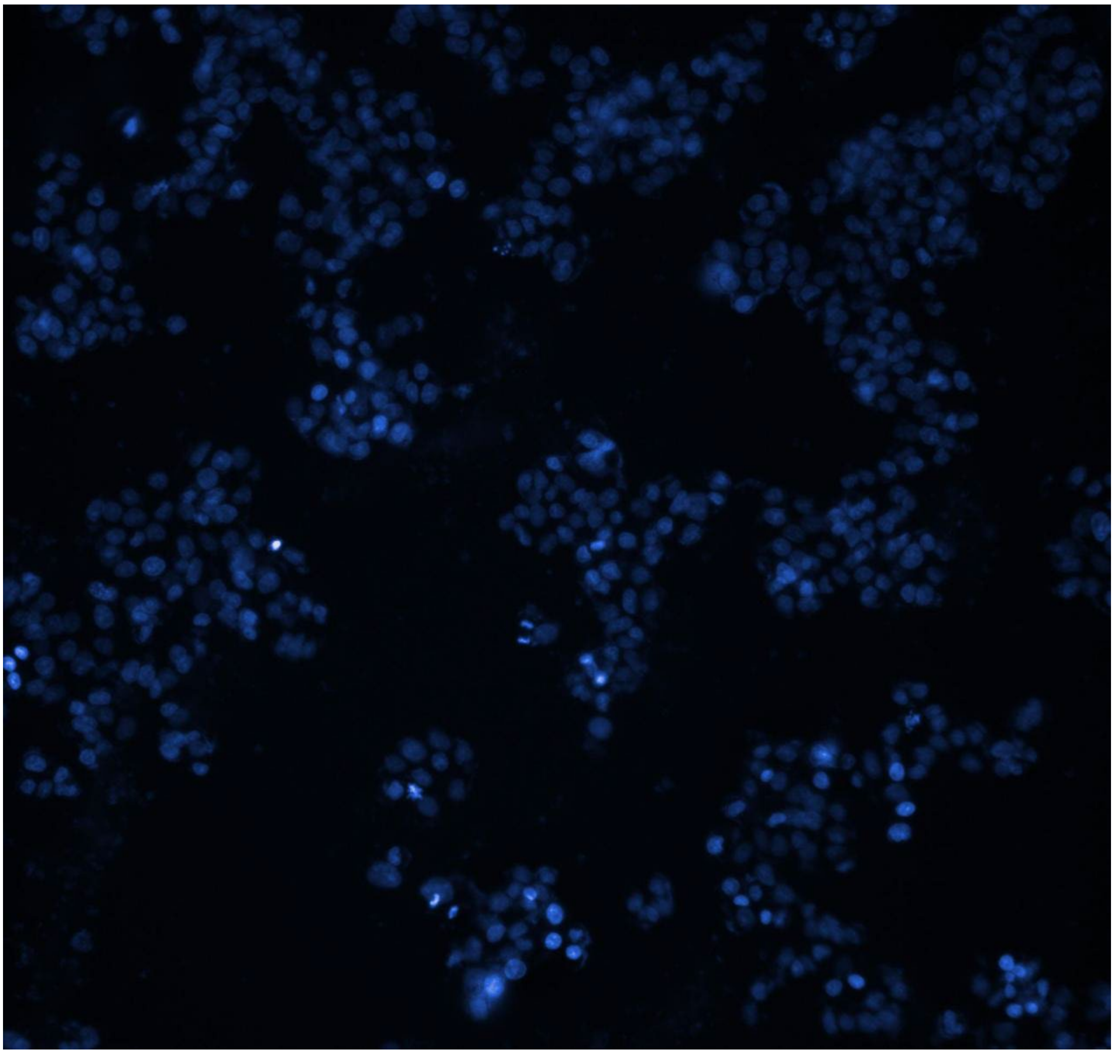

(b)

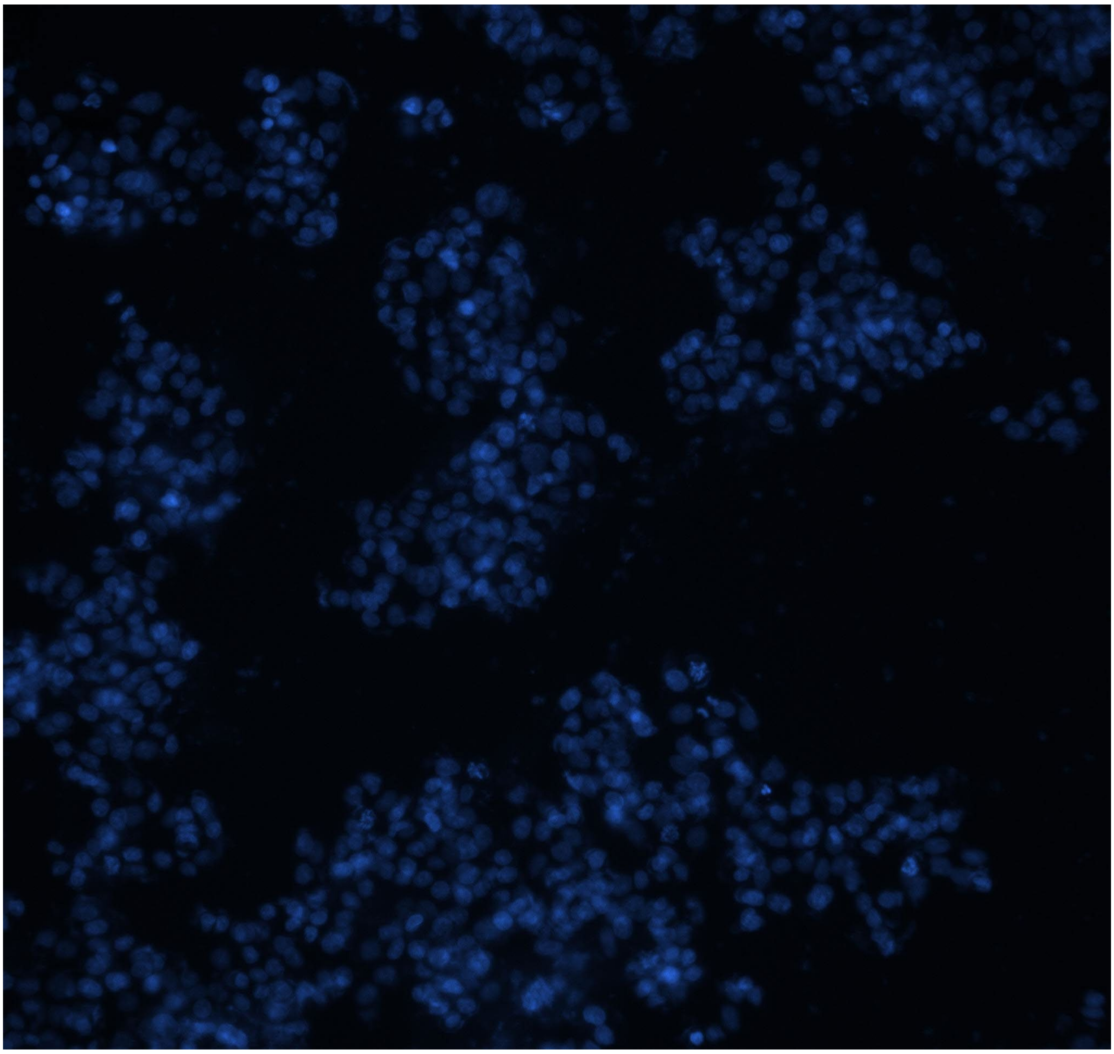

(c)

Figure S10. The intracellular fluorescence measurement results of Hoechest in CGA - GL group. (a)2 h. (b)4 h. (c)6 h.

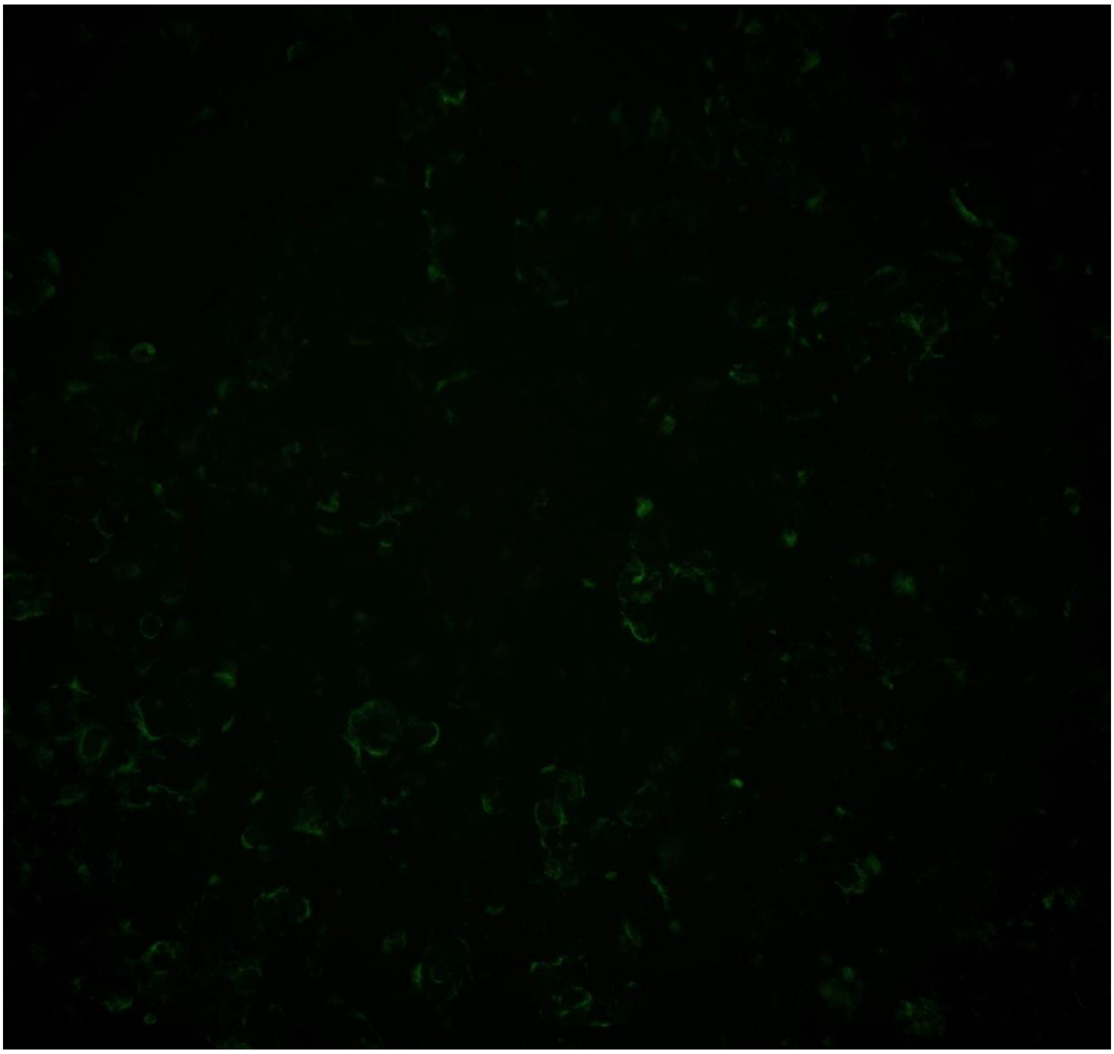

(a)

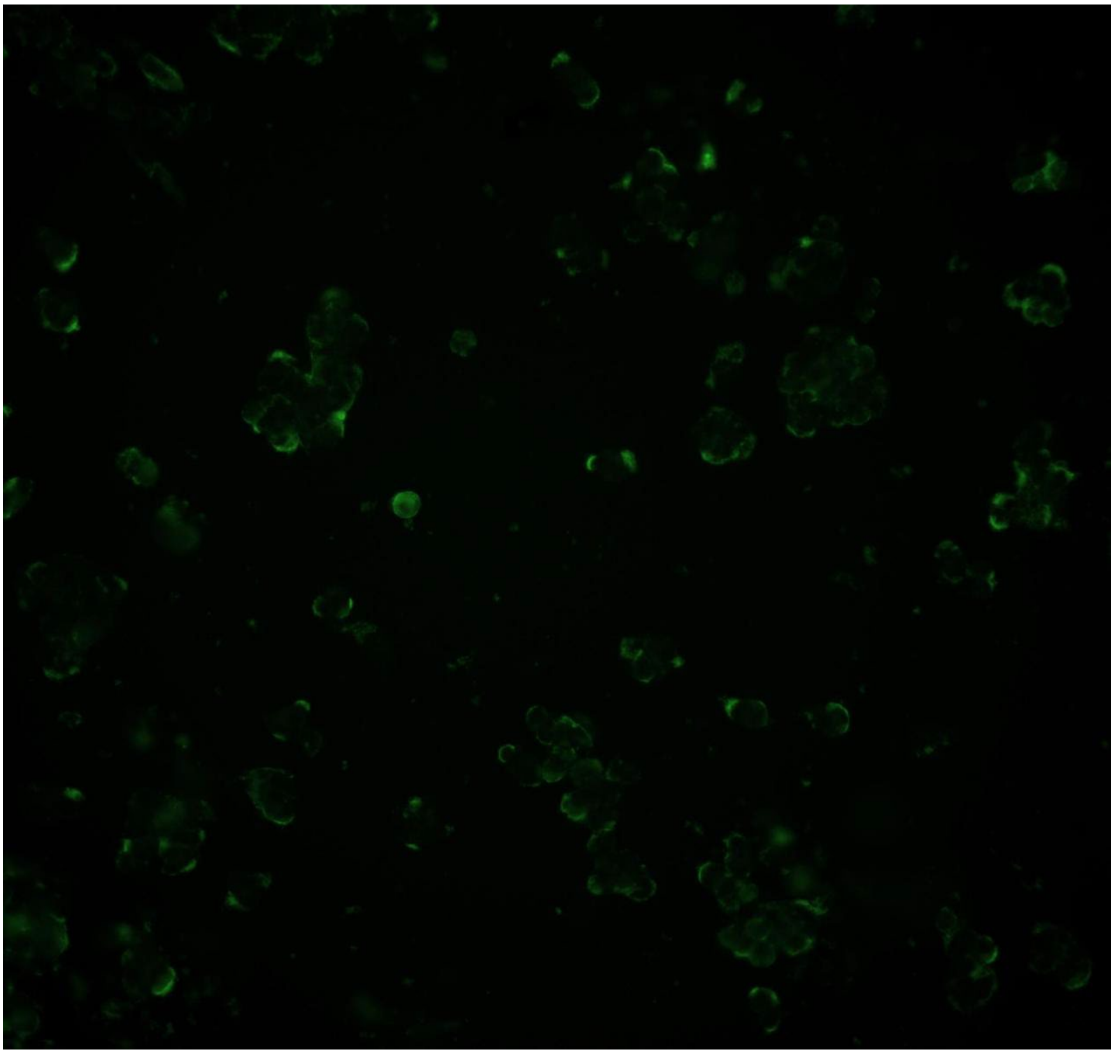

(b)

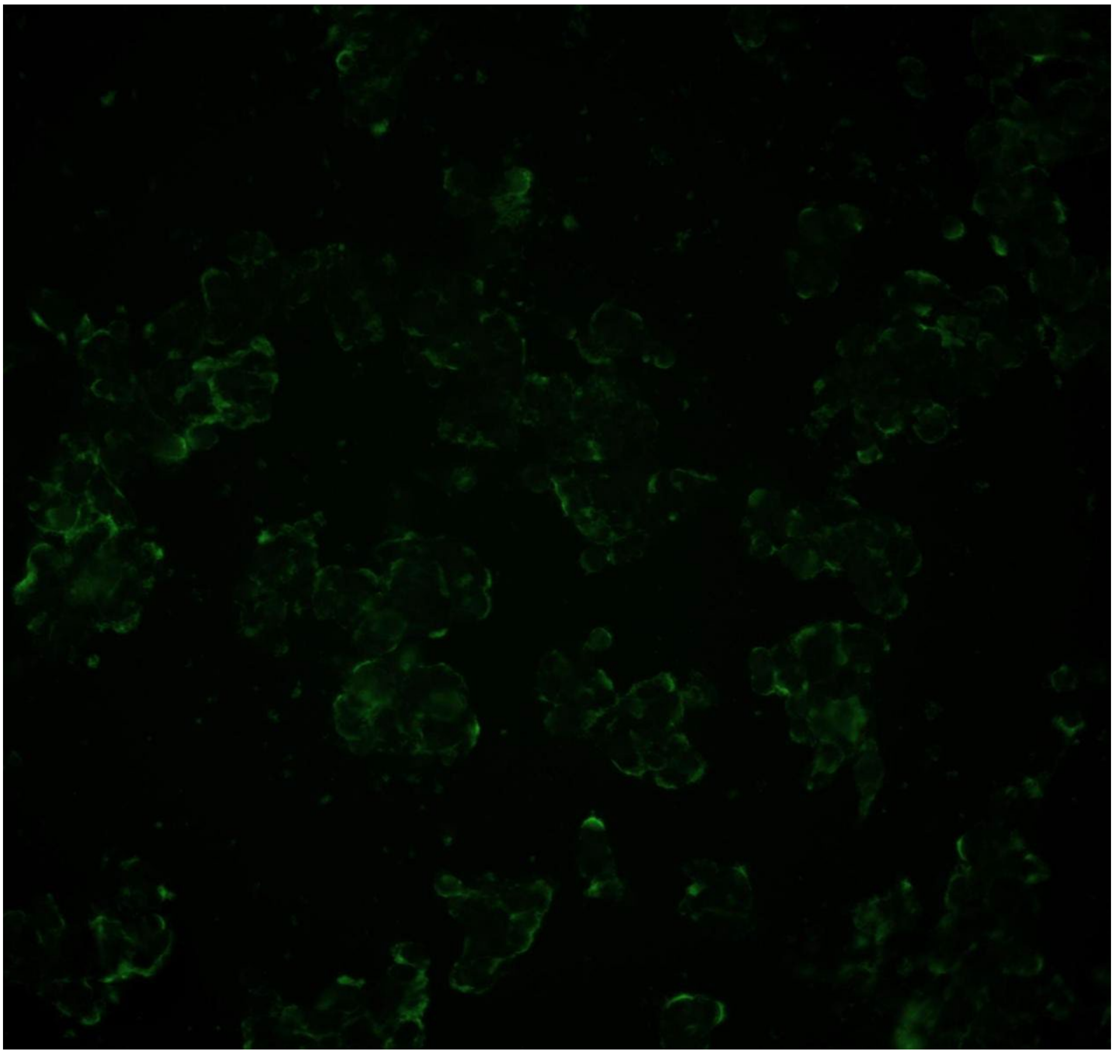

(c)

Figure S11. The intracellular fluorescence measurement results of Cou6 in Cou6-sol group. (a)2 h. (b)4 h. (c)6 h.

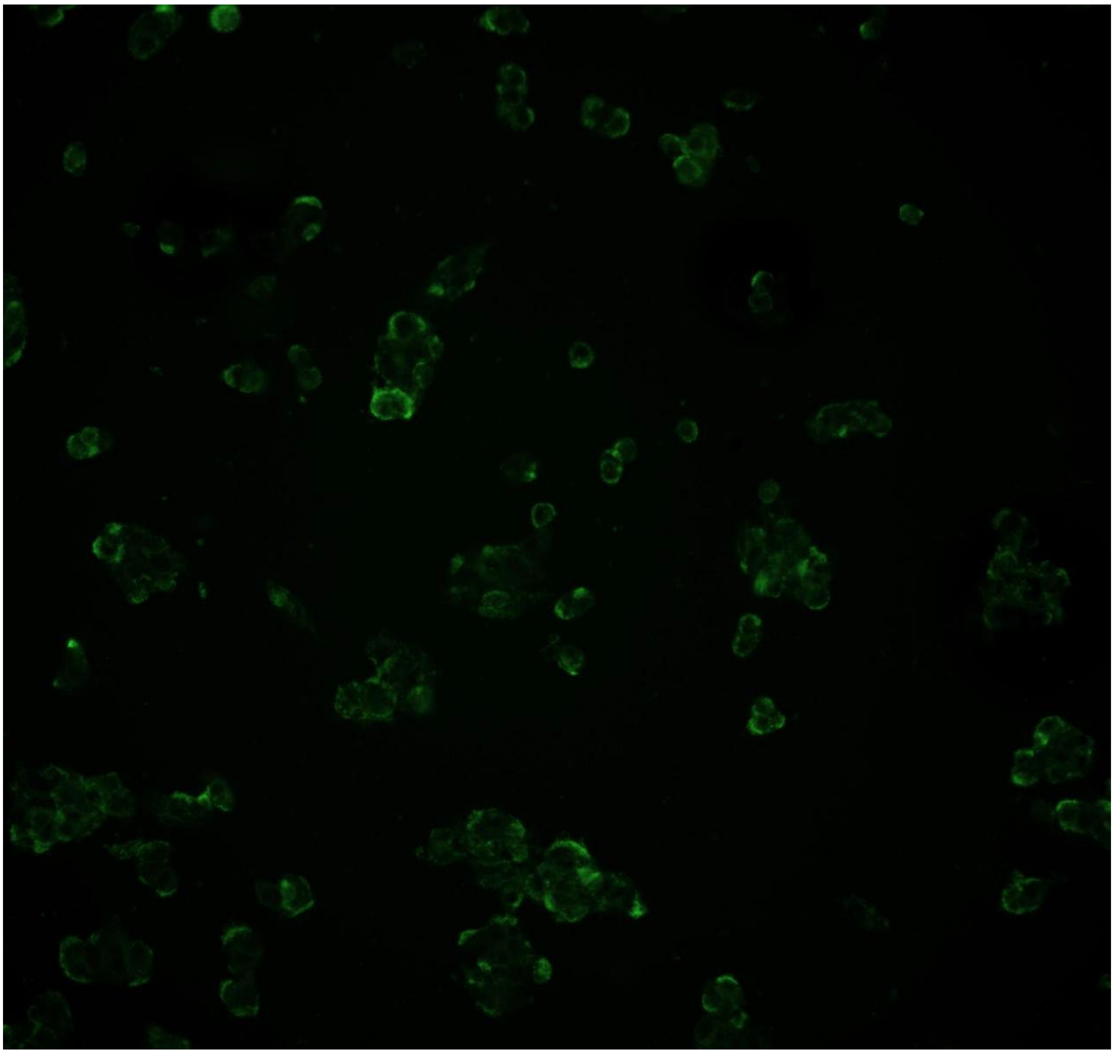

(a)

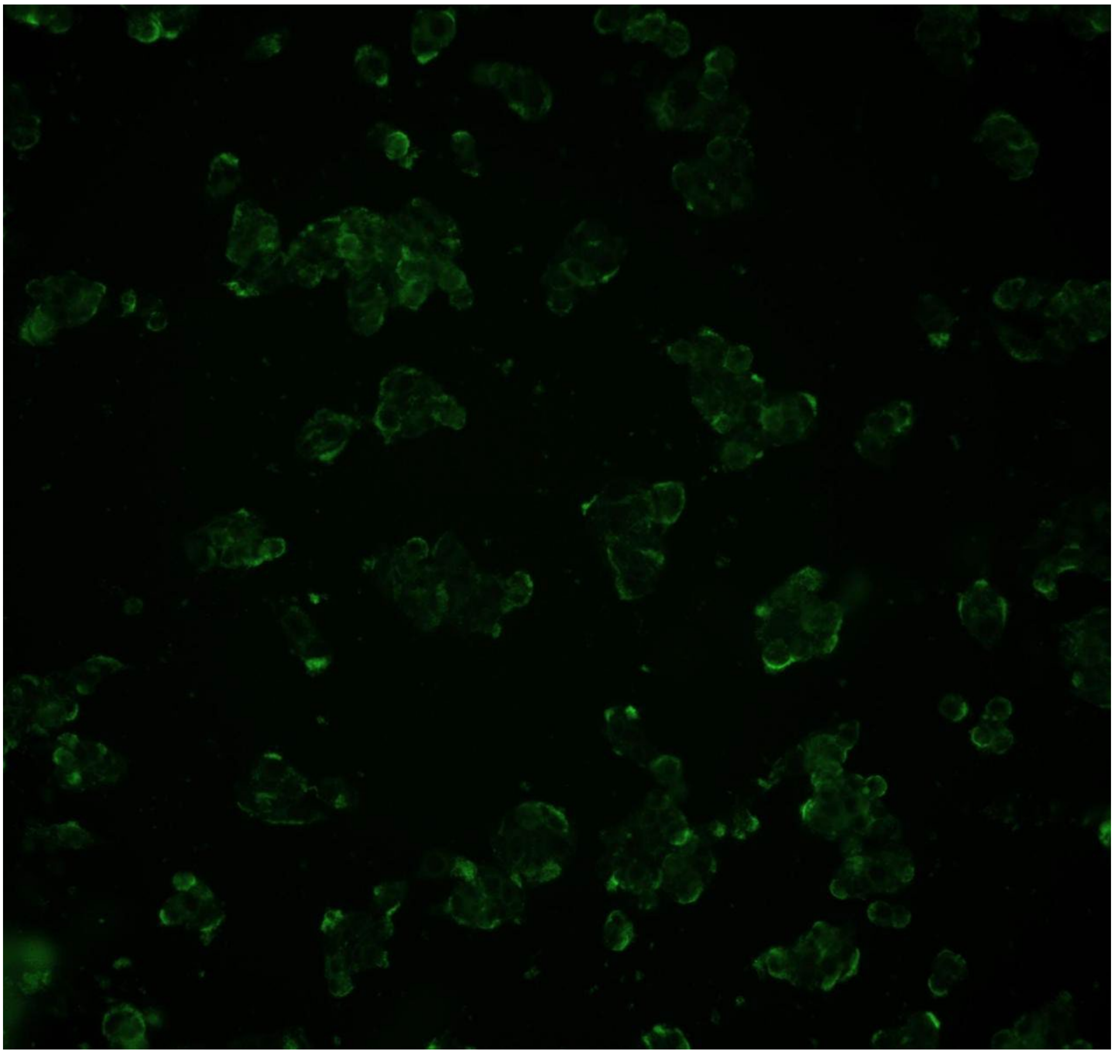

(b)

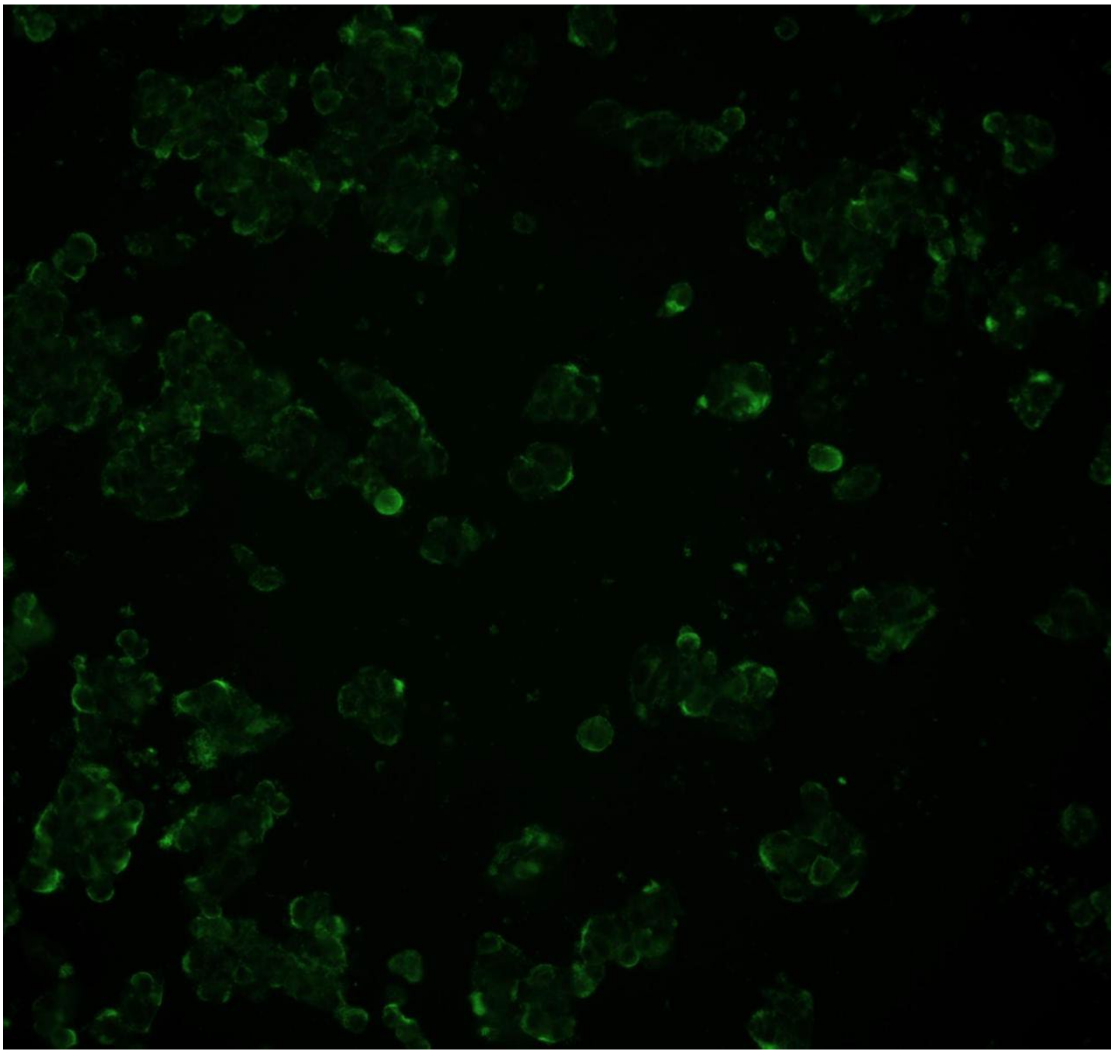

(c)

Figure S12. The intracellular fluorescence measurement results of Cou6 in Cou6-GL group. (a)2 h. (b)4 h. (c)6 h.

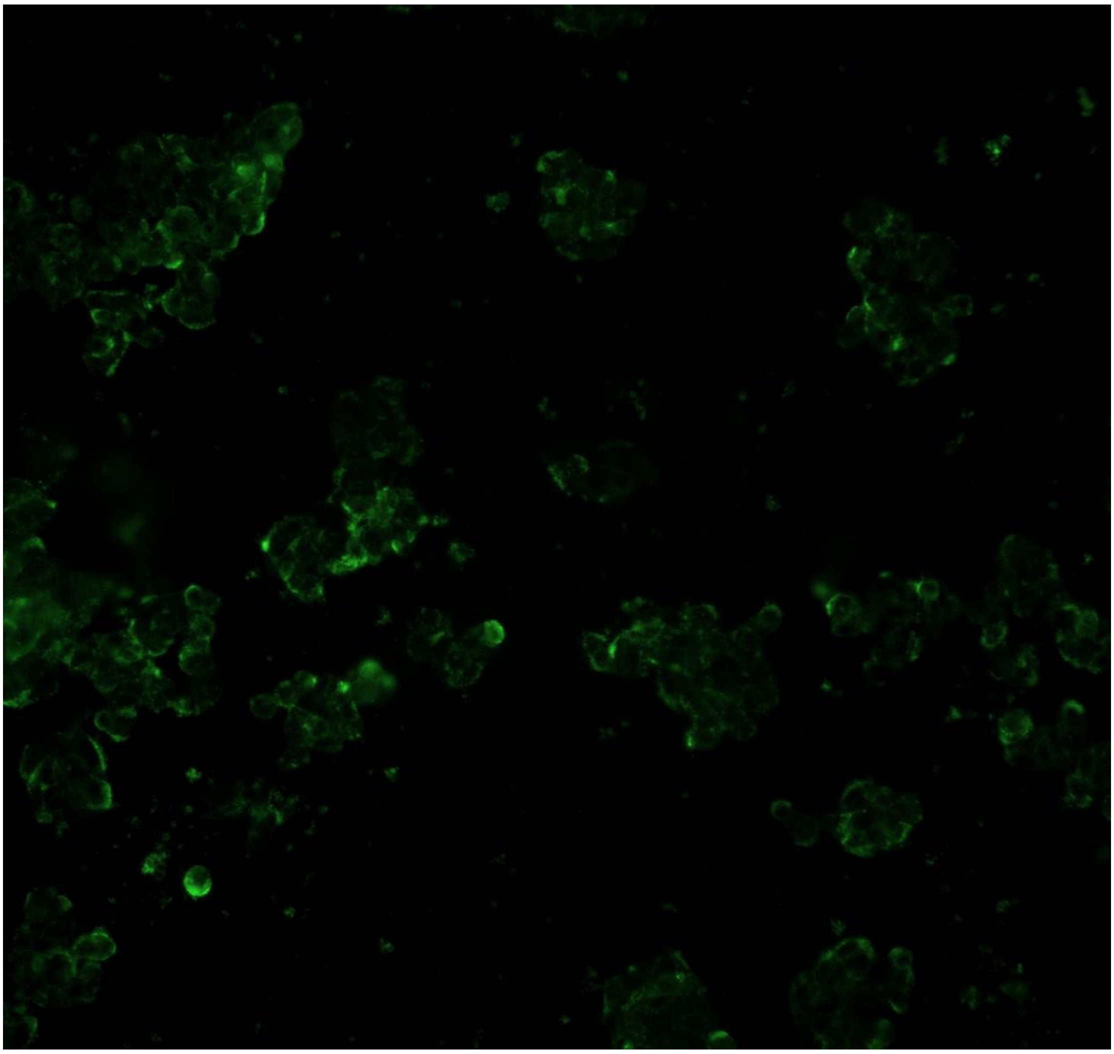

(a)

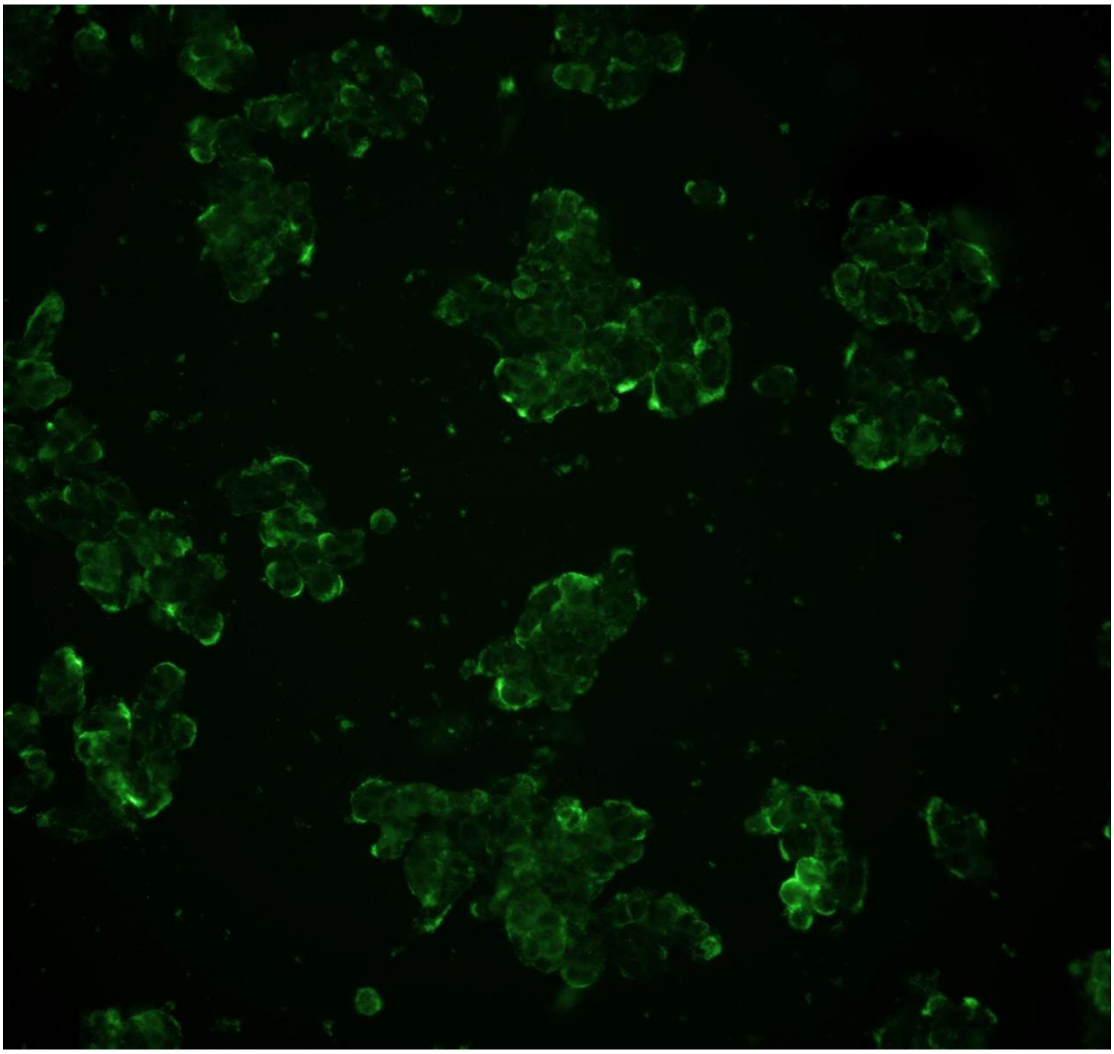

(b)

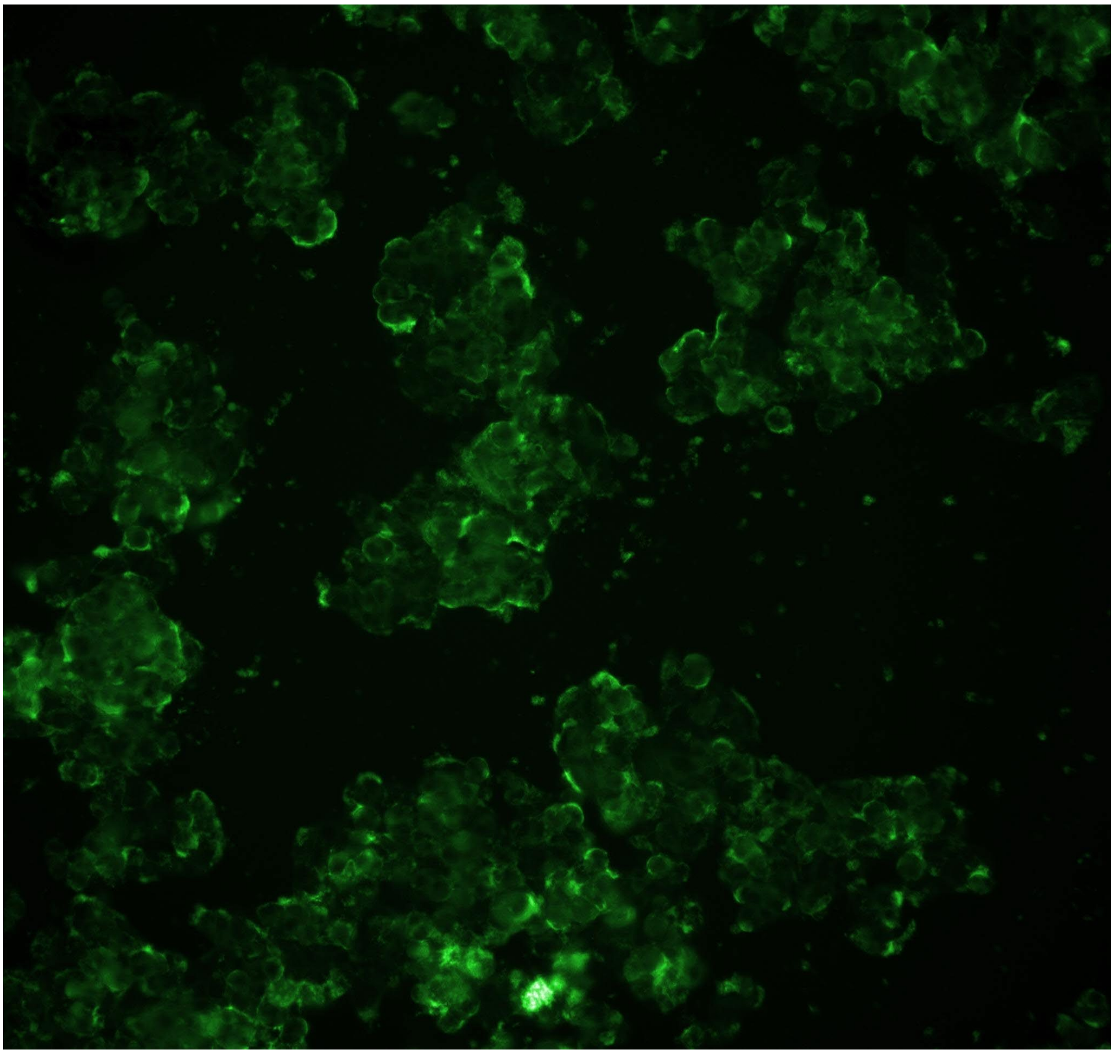

(c)

Figure S13. The intracellular fluorescence measurement results of Cou6 in Cou6/GA - GL group. (a)2 h. (b)4 h. (c)6 h.

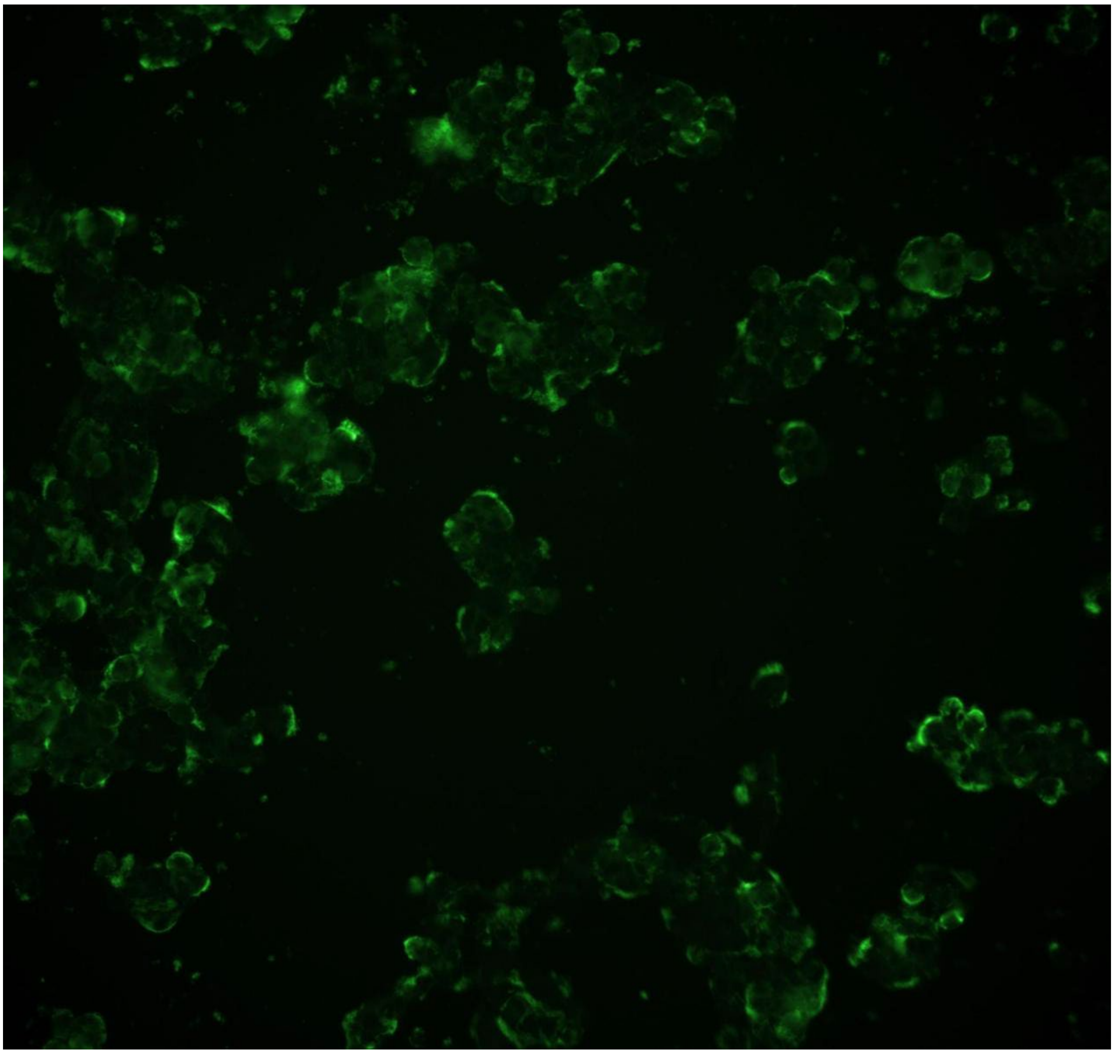

(a)

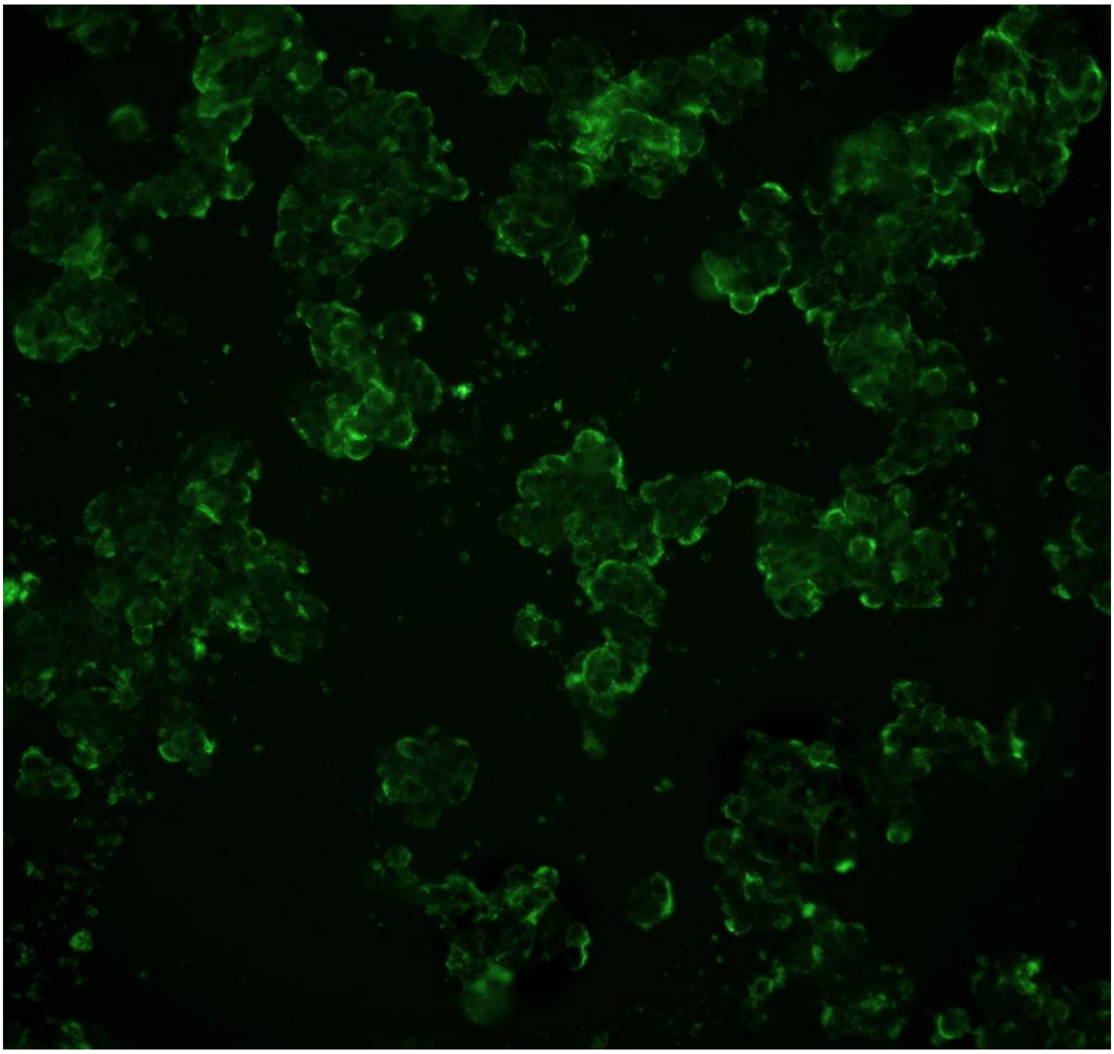

(b)

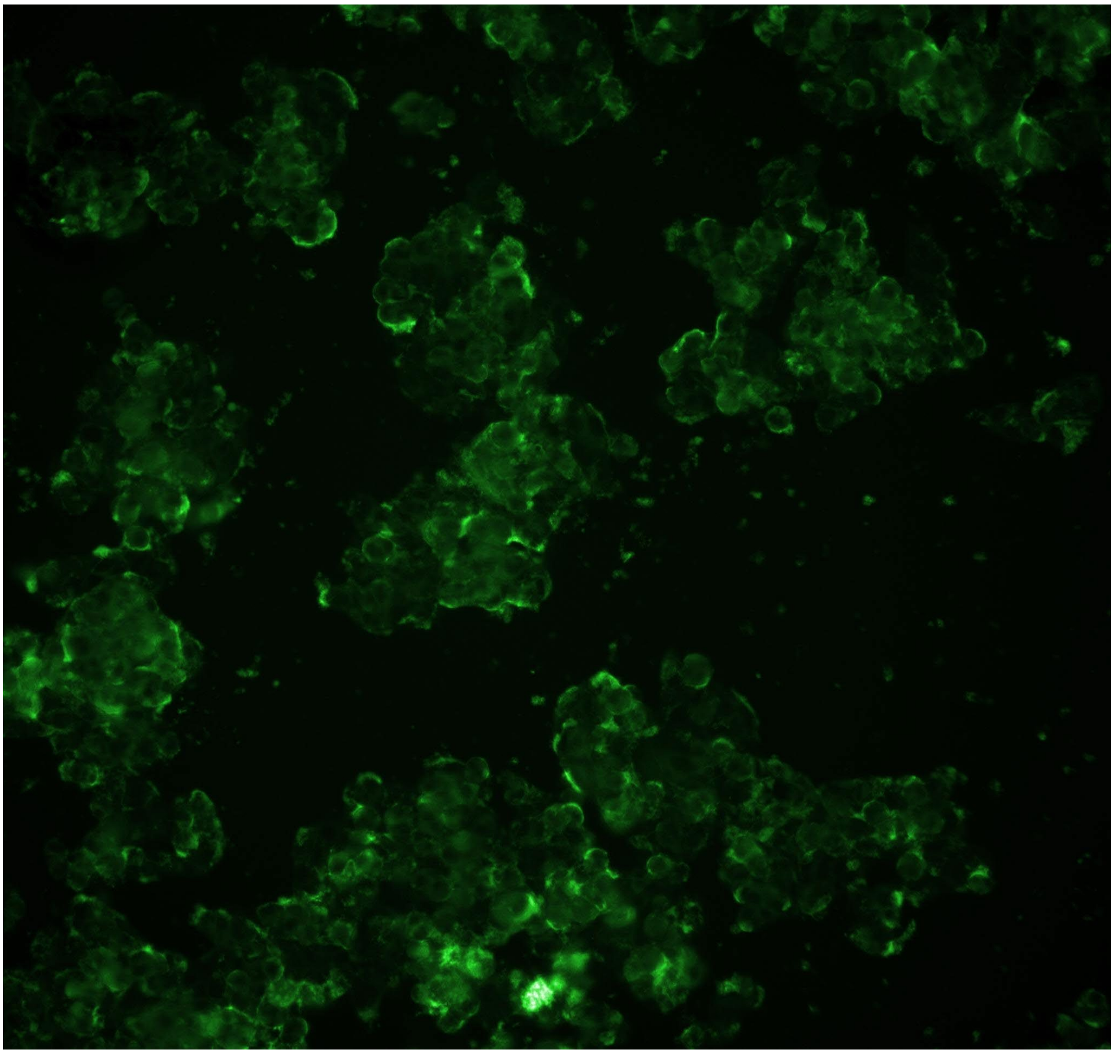

(c)

Figure S14. The intracellular fluorescence measurement results of Cou6 in CGA - GL group. (a)2 h. (b)4 h. (c)6 h.

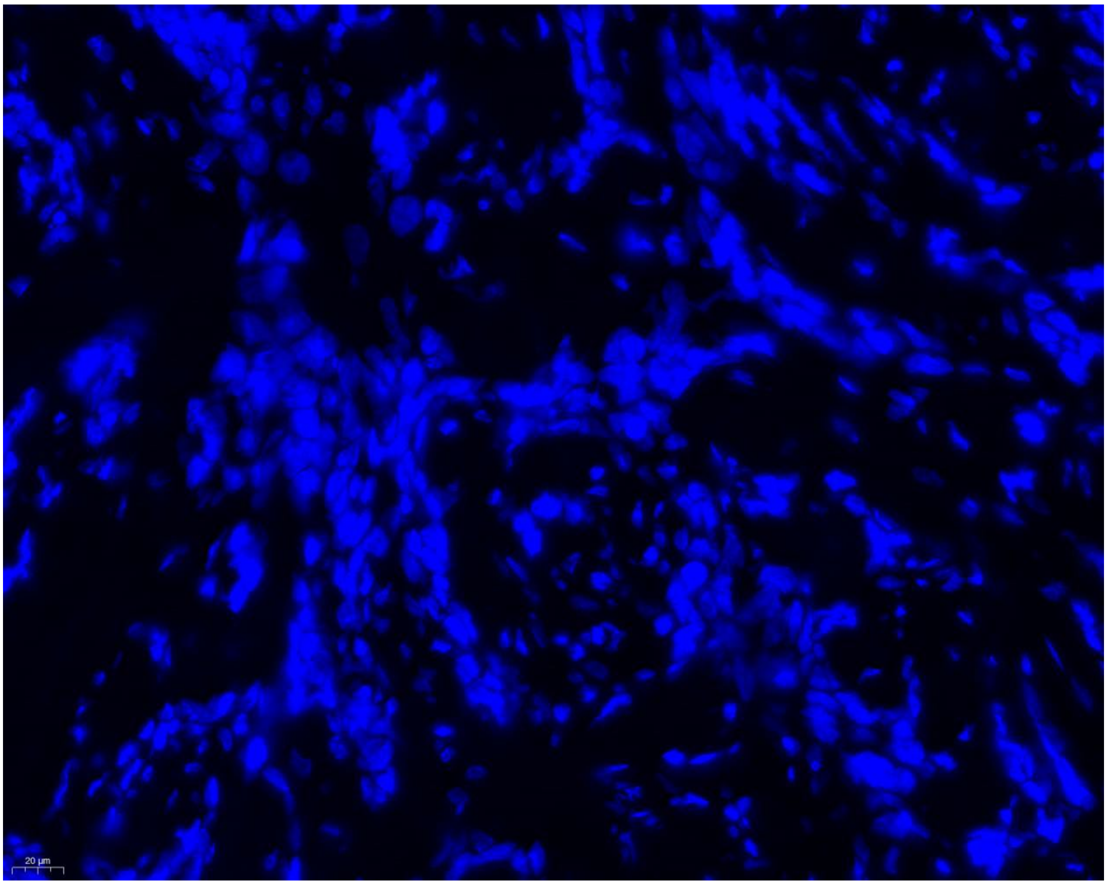

(a)

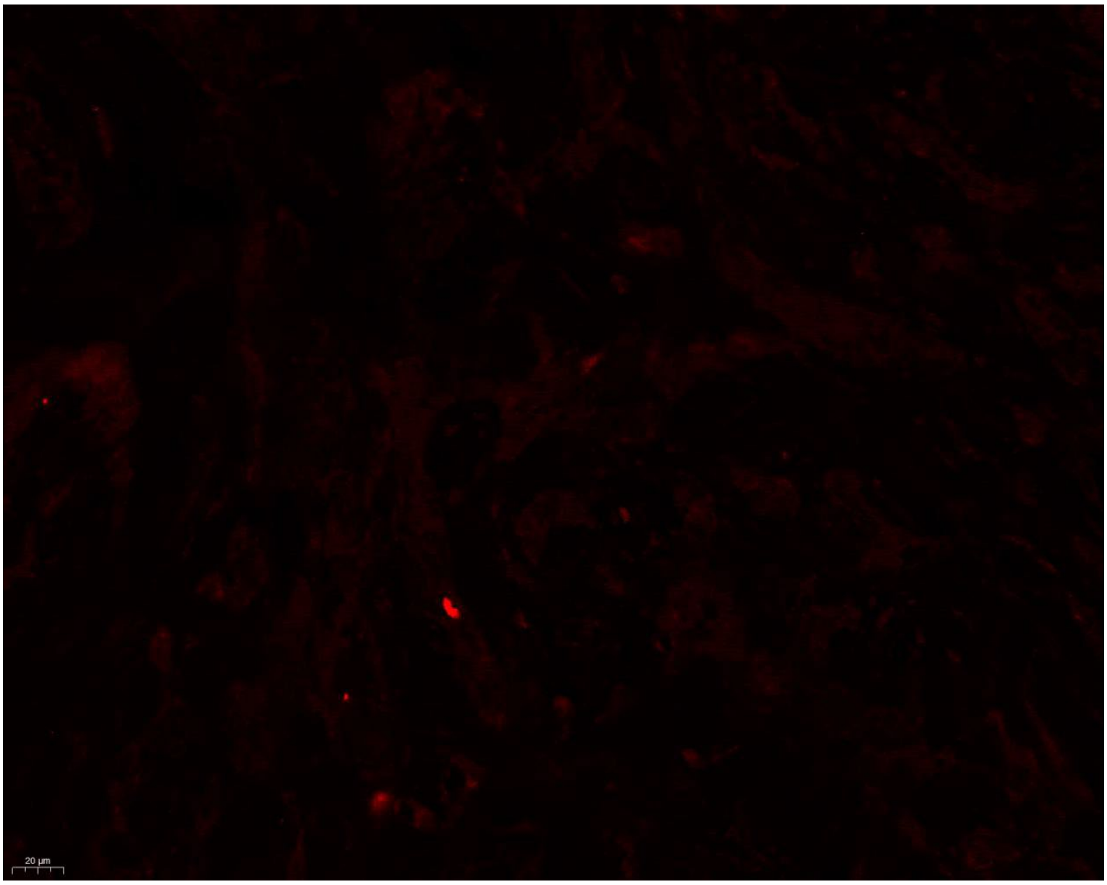

(b)

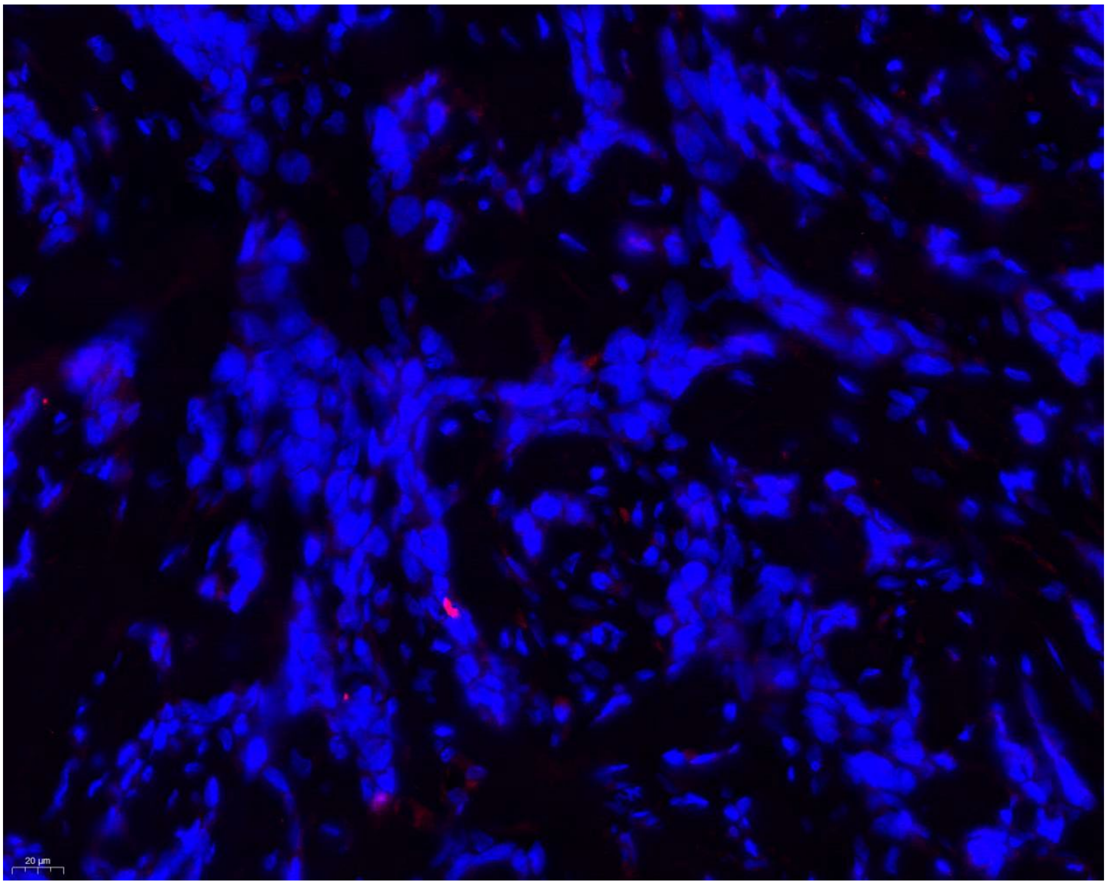

(c)

Figure S15. TUNEL test results of Saline group. (a) DAPI. (b) TUNEL. (c) Merge.

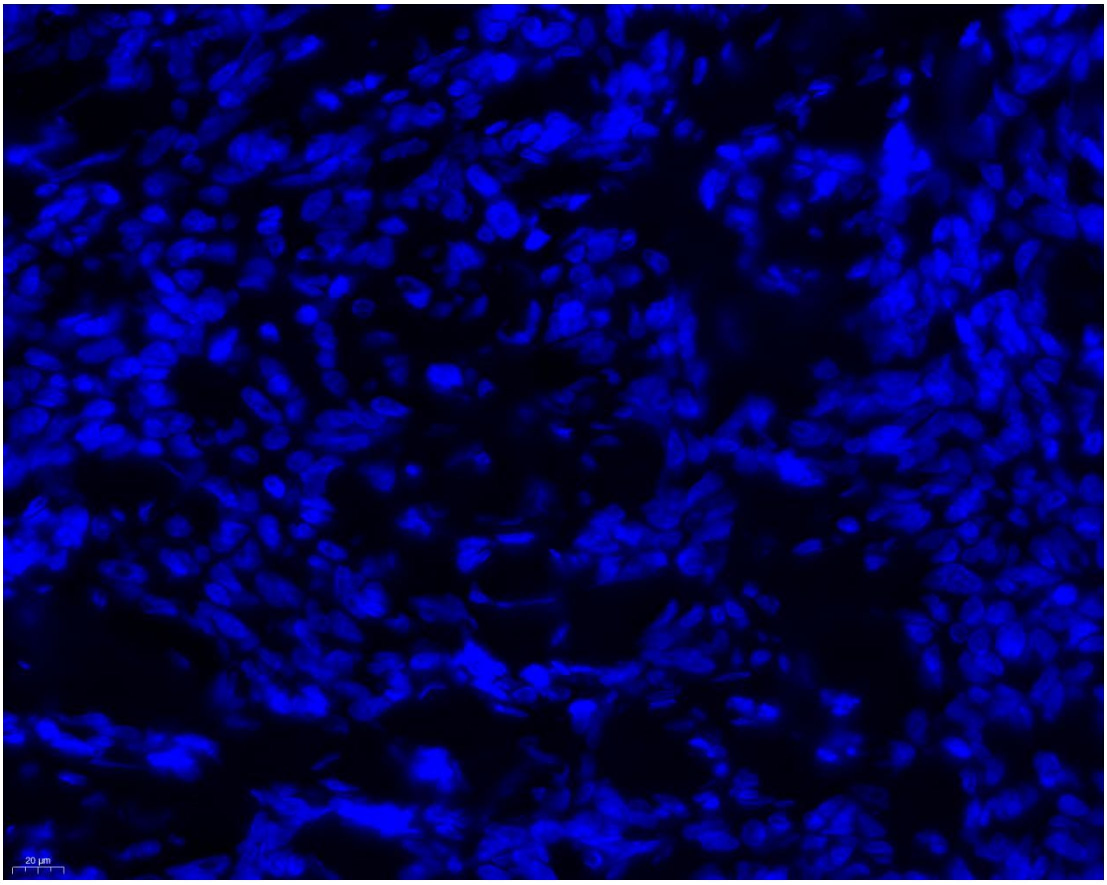

(a)

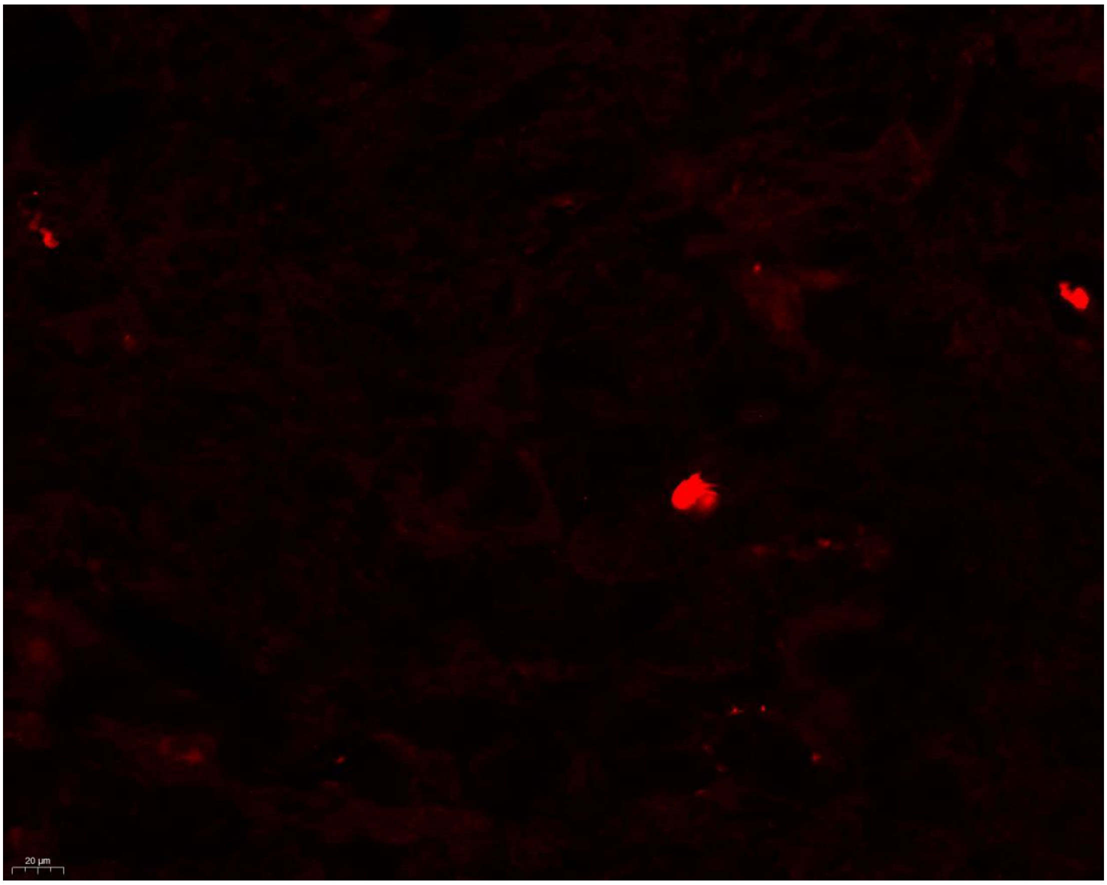

(b)

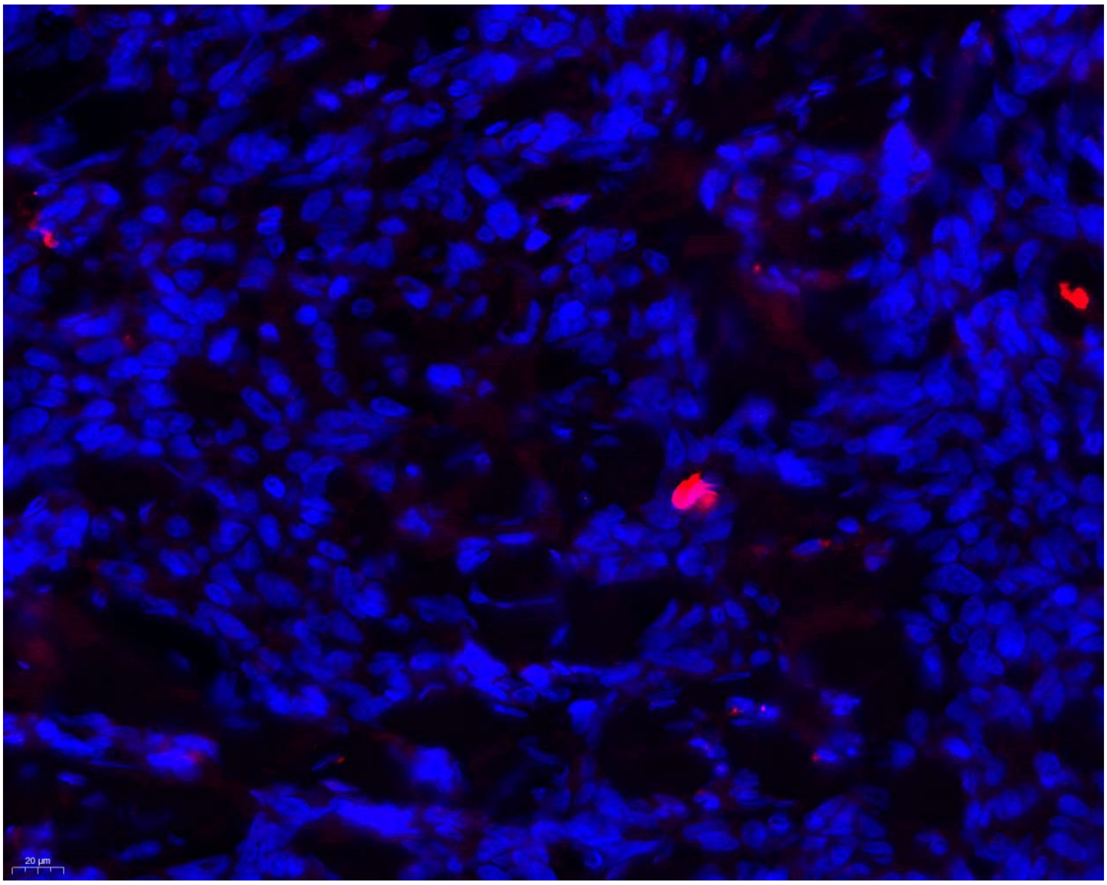

(c)

Figure S16. TUNEL test results of CUR sol group. (a) DAPI. (b) TUNEL. (c) Merge.

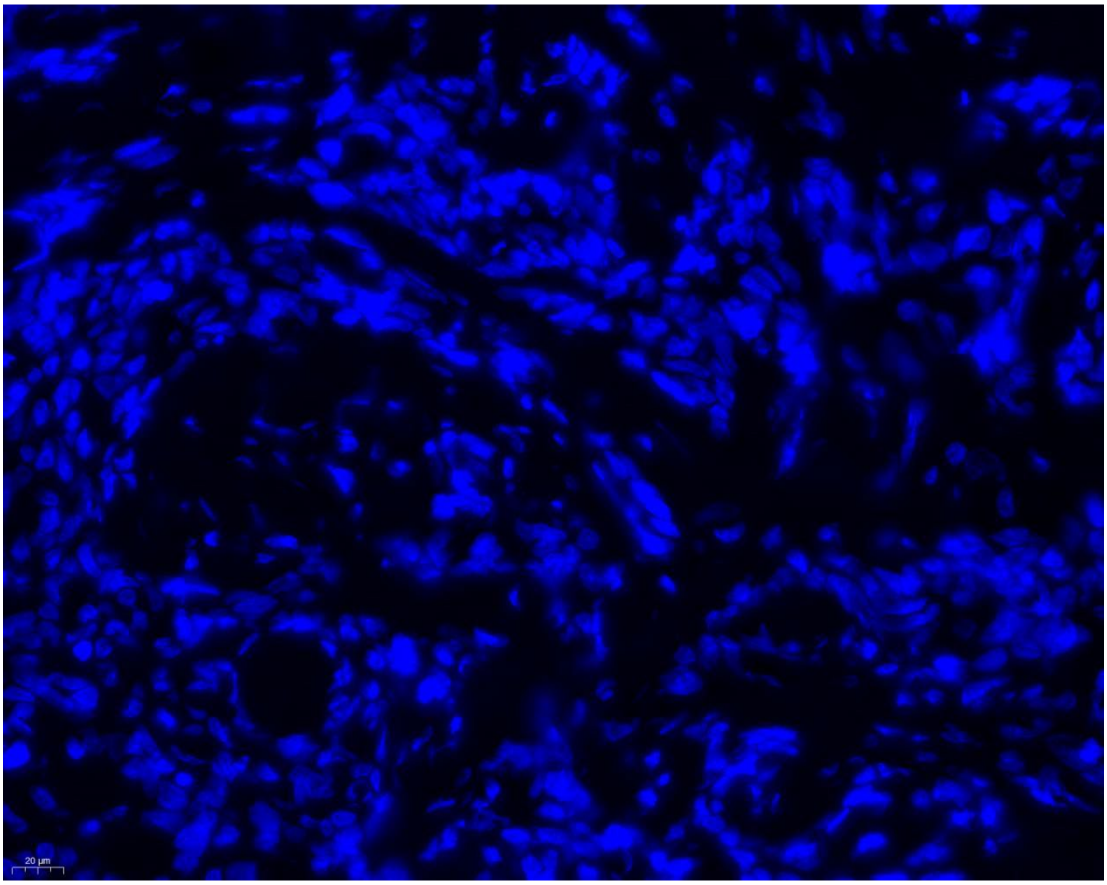

(a)

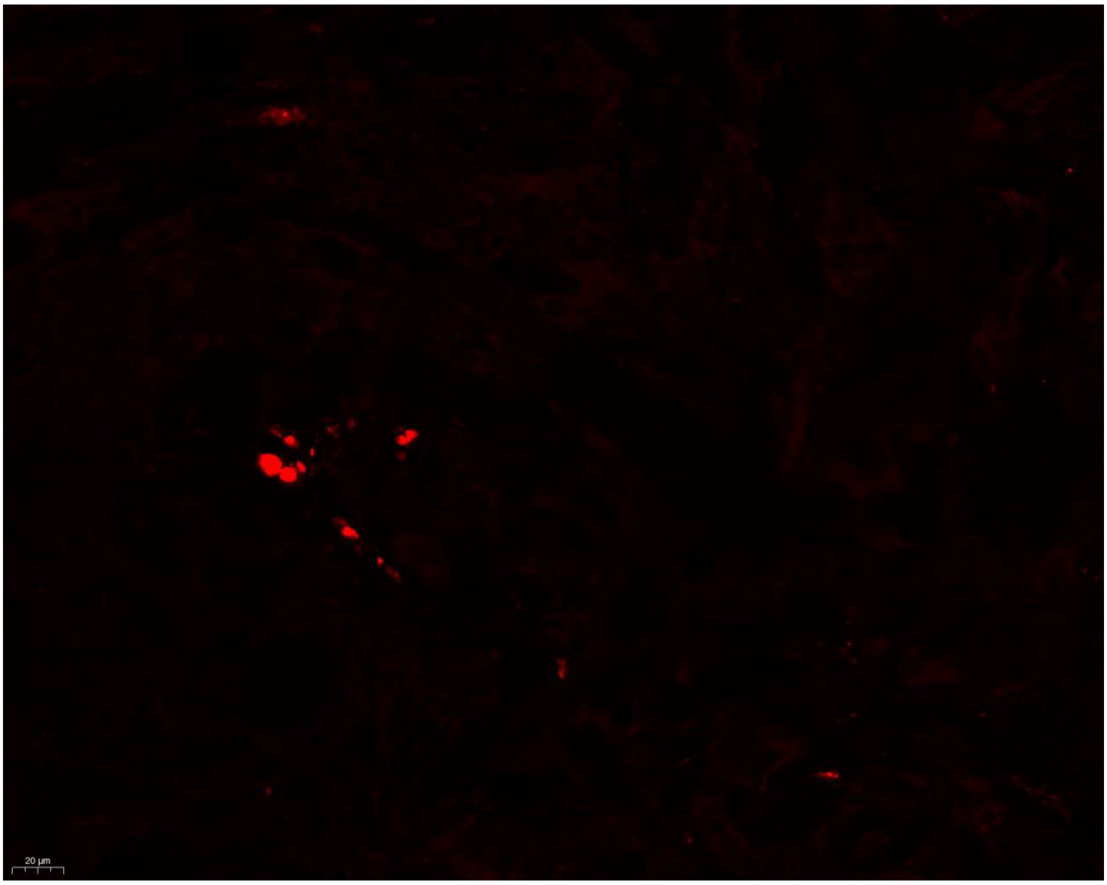

(b)

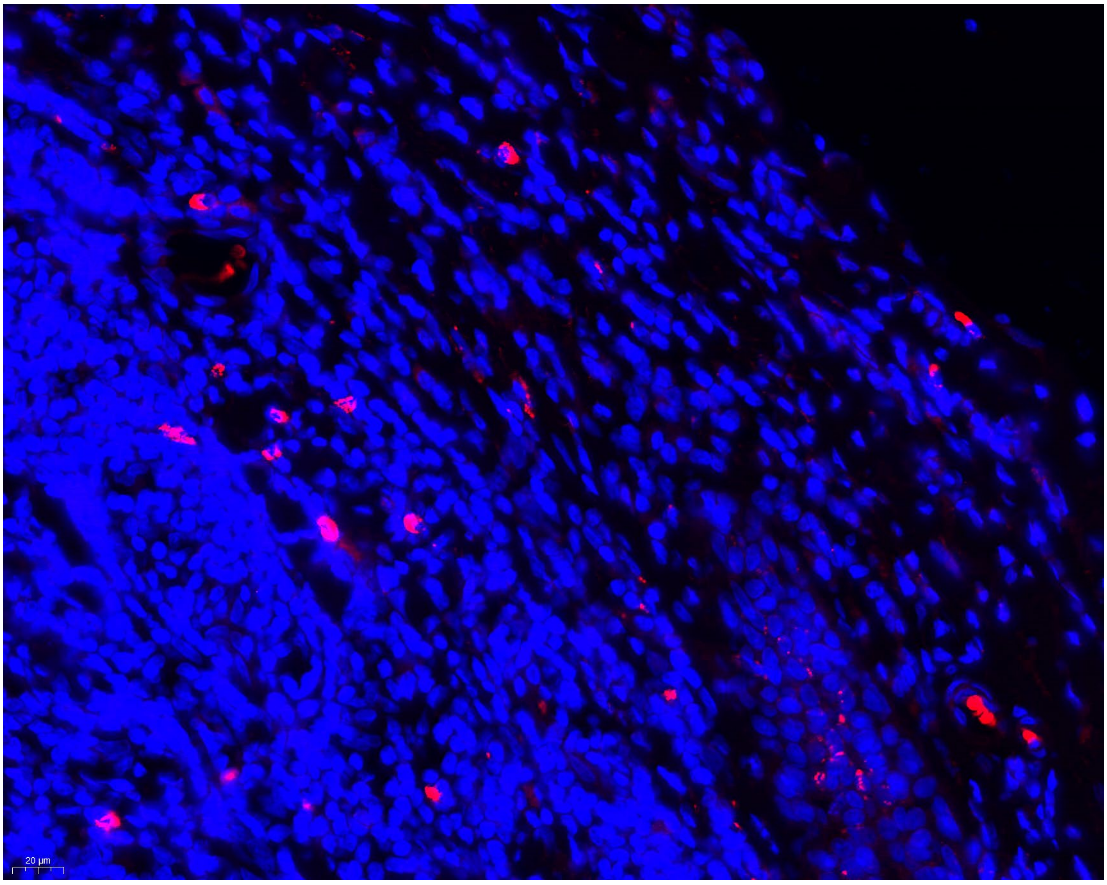

(c)

Figure S17. TUNEL test results of GA - GL micelles group. (a) DAPI. (b) TUNEL. (c) Merge.

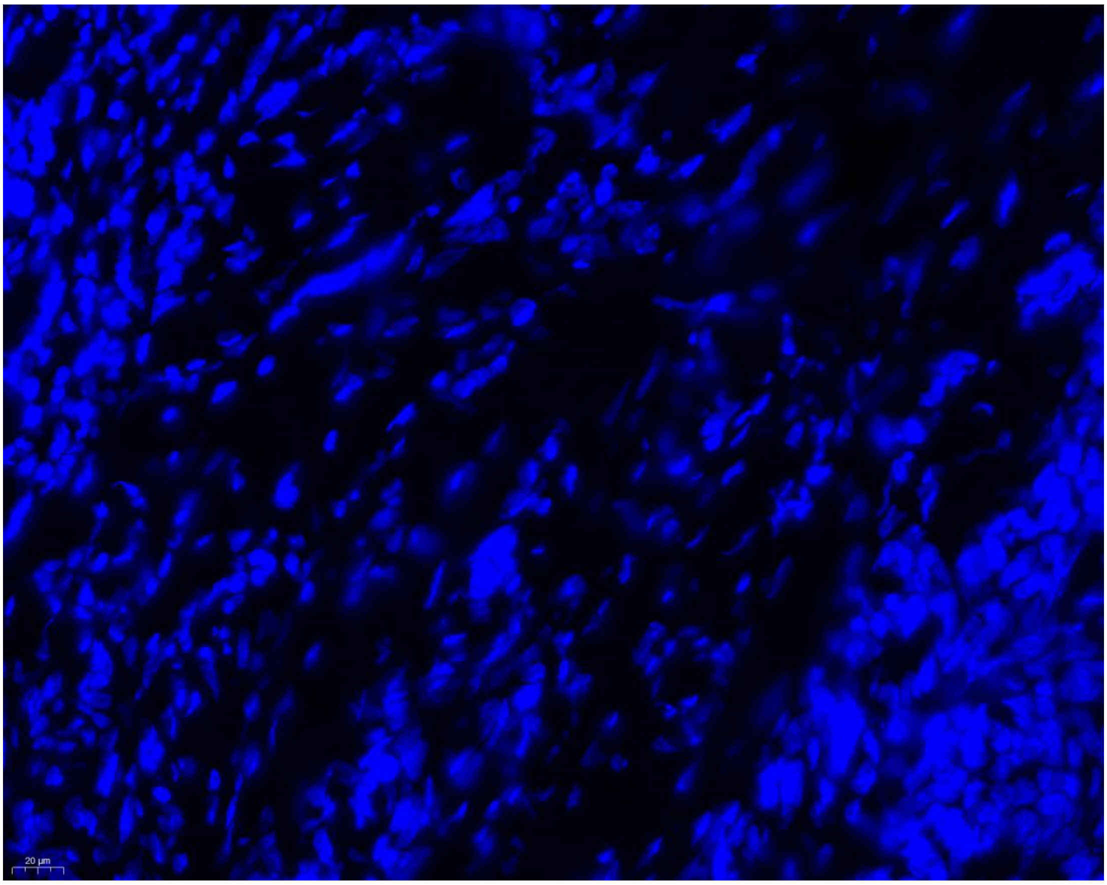

(a)

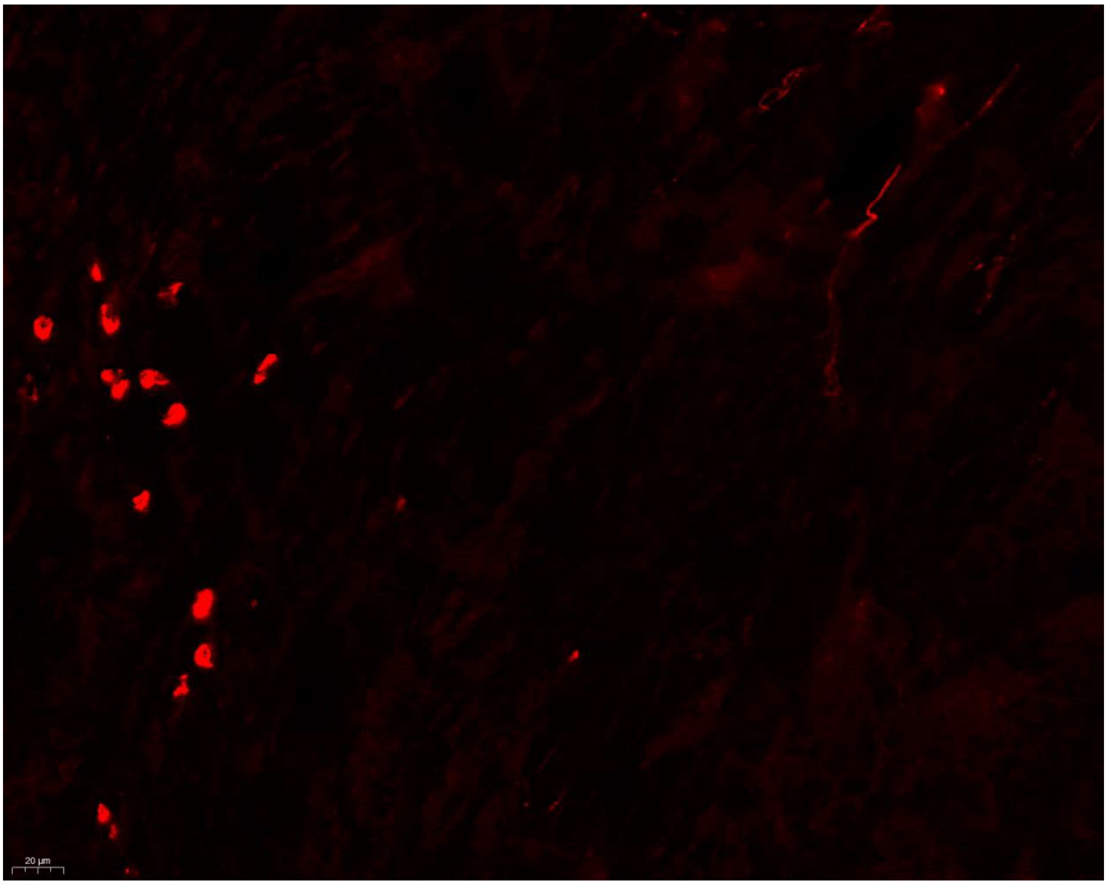

(b)

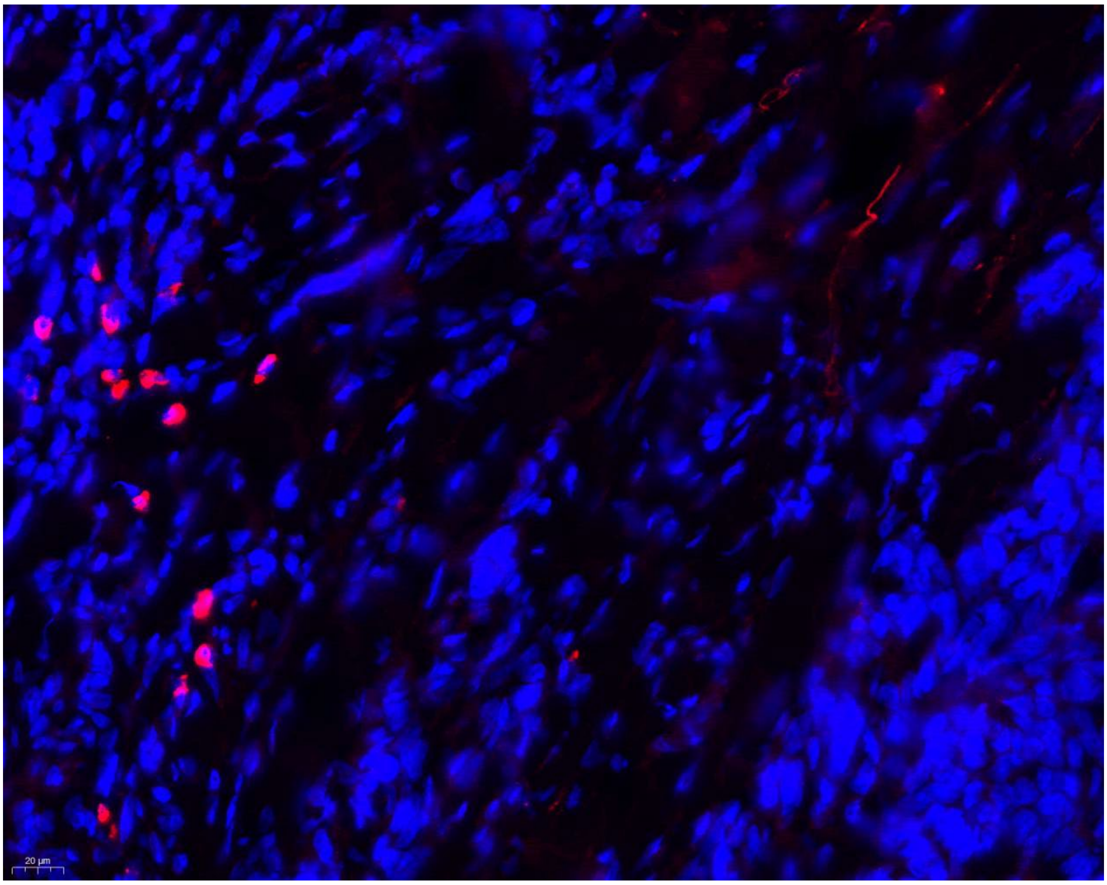

(c)

Figure S18. TUNEL test results of CUR/GA - GL micelles group. (a) DAPI. (b) TUNEL. (c) Merge.

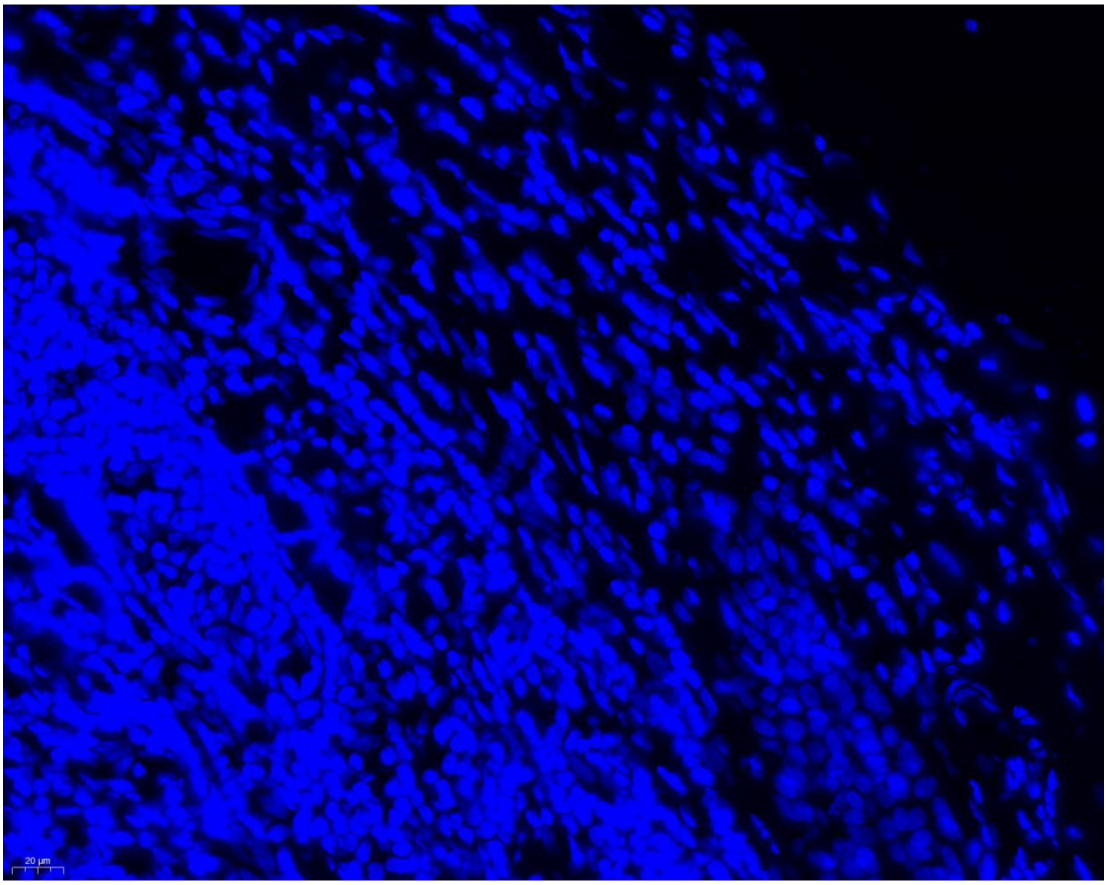

(a)

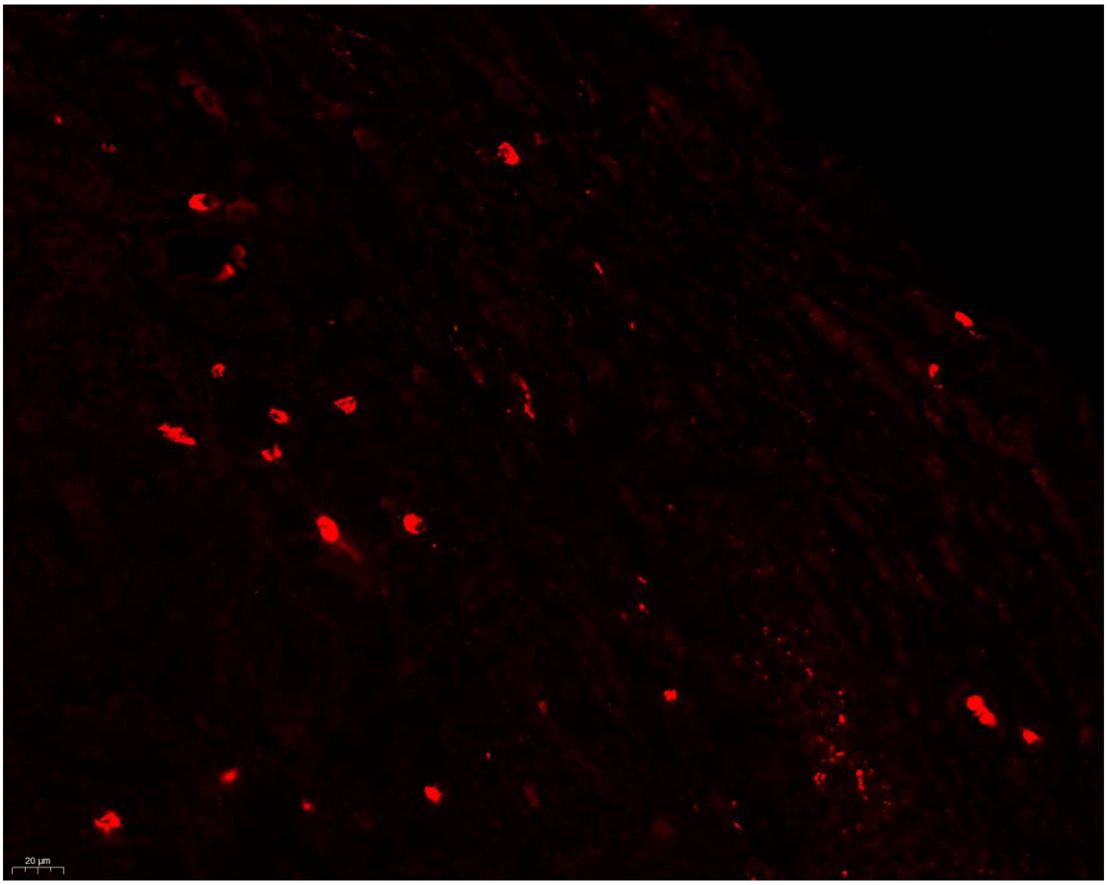

(b)

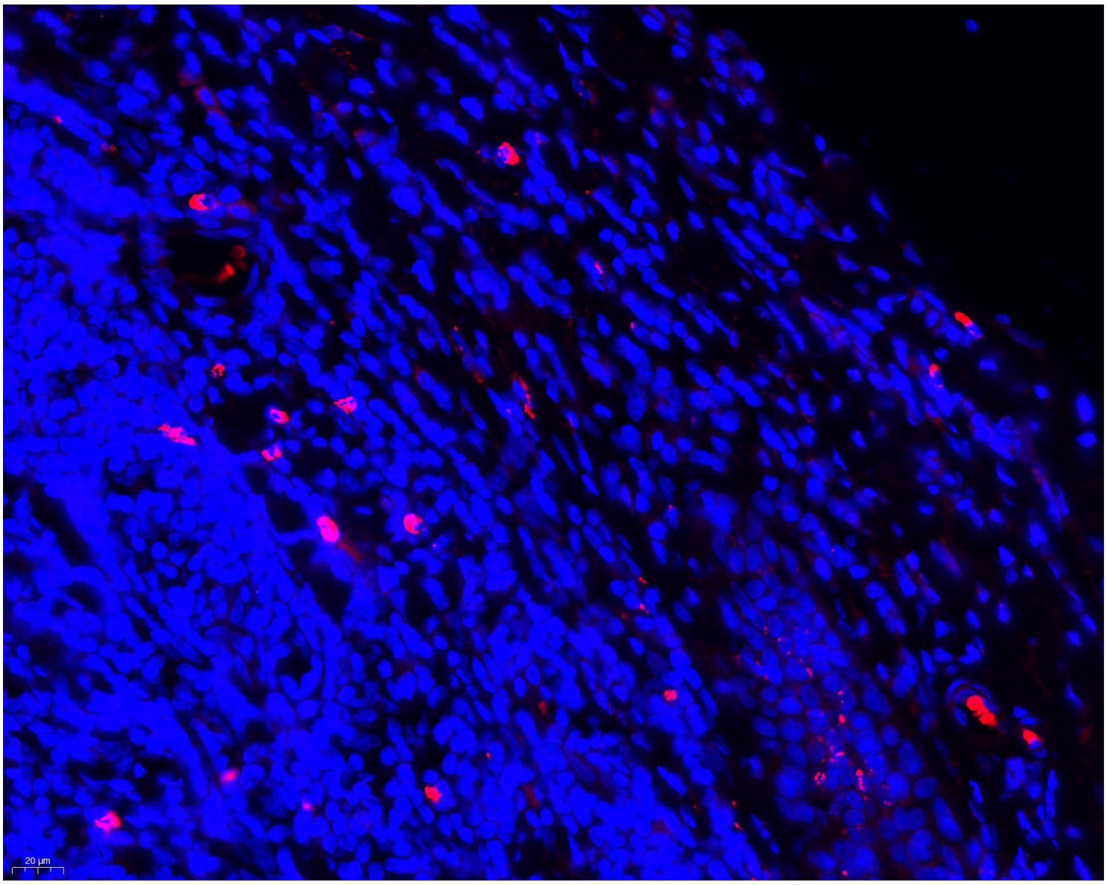

(c)

Figure S19. TUNEL test results of CGA - GL micelles solution group. (a) DAPI. (b) TUNEL. (c) Merge.

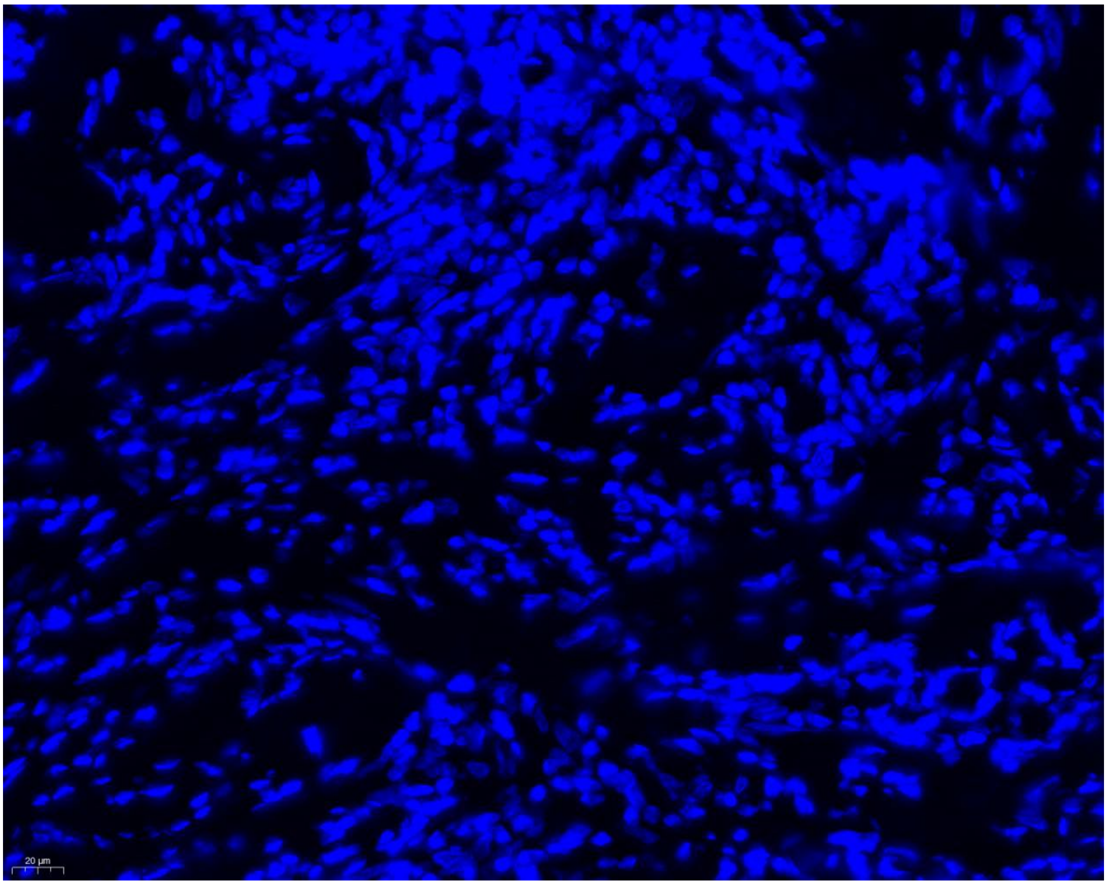

(a)

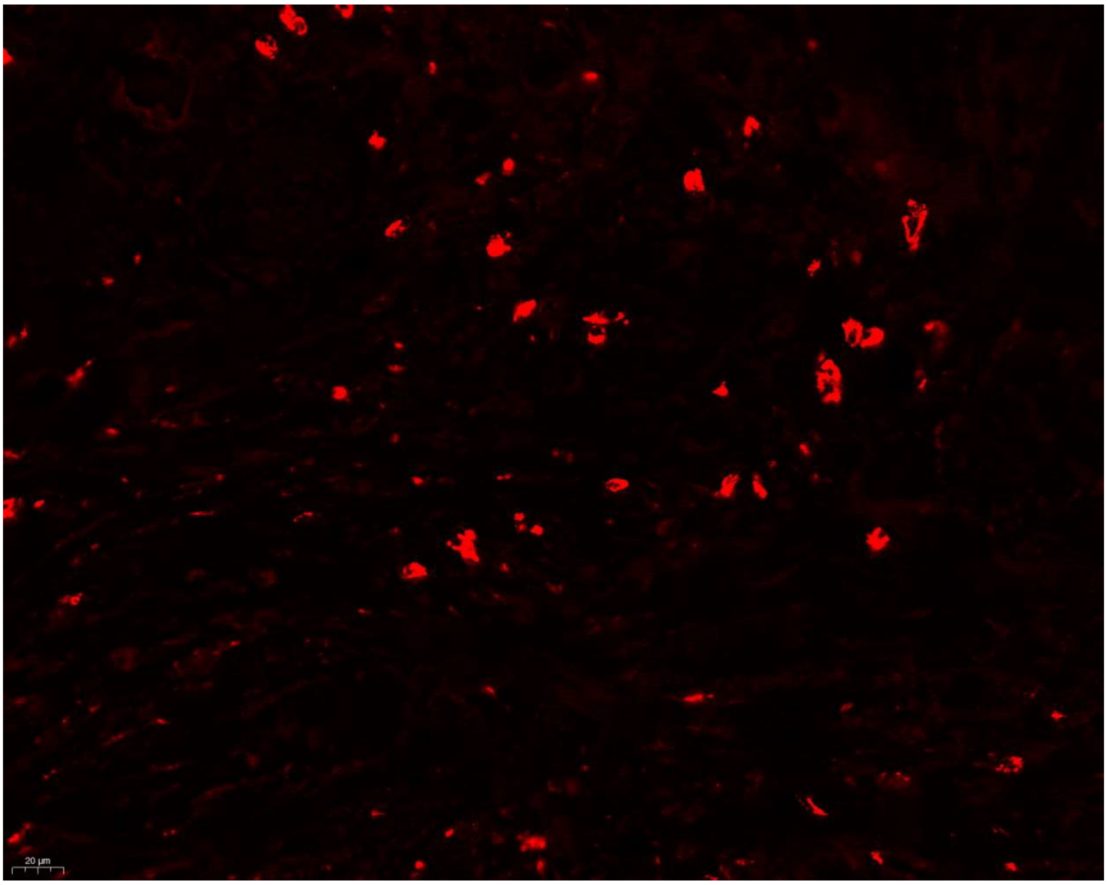

(b)

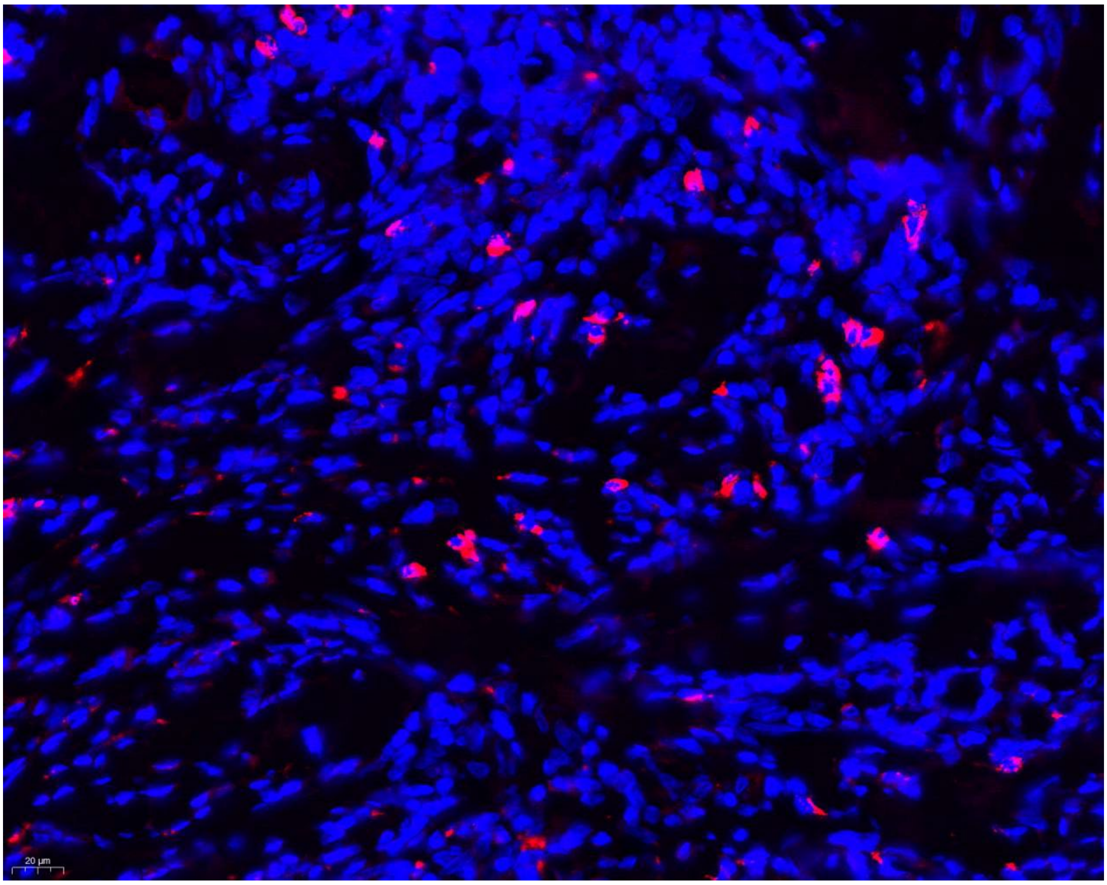

(c)

Figure S20. TUNEL test results of PTX solution group. (a) DAPI. (b) TUNEL. (c) Merge.

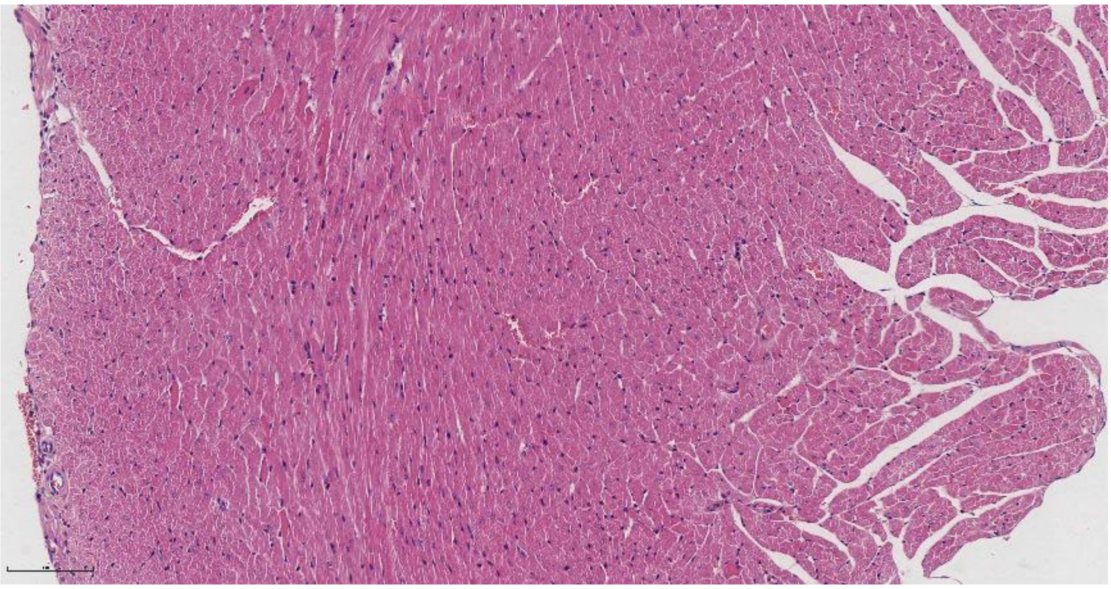

(a)

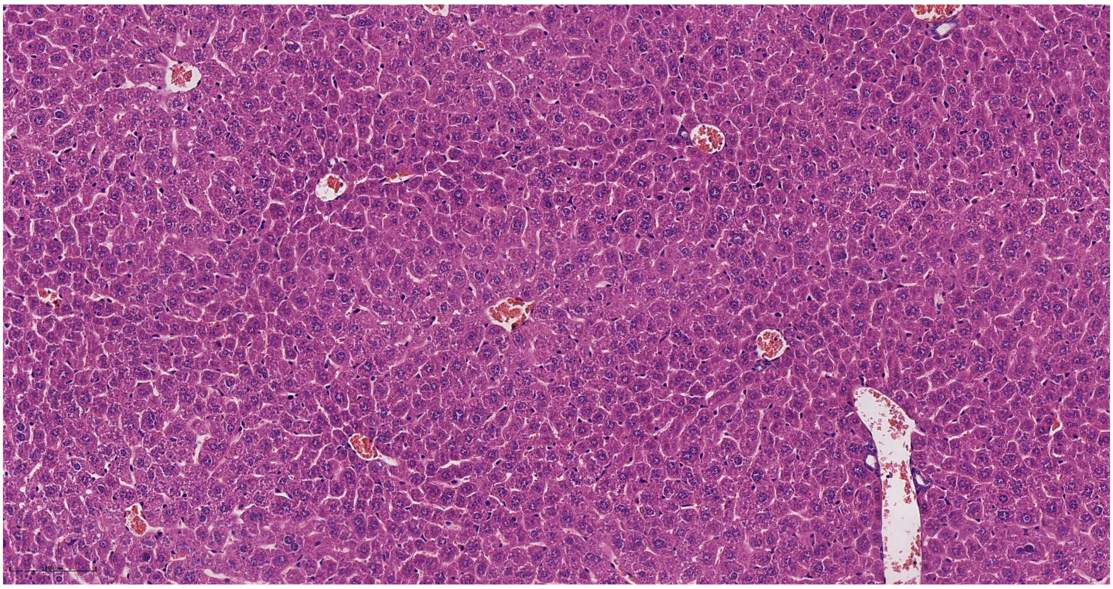

(b)

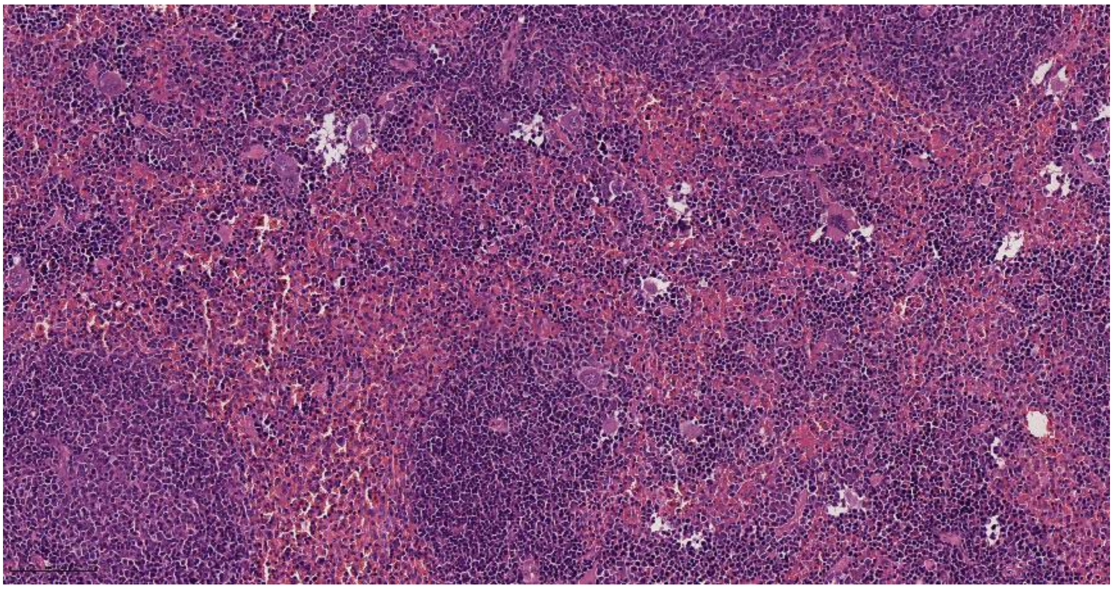

(c)

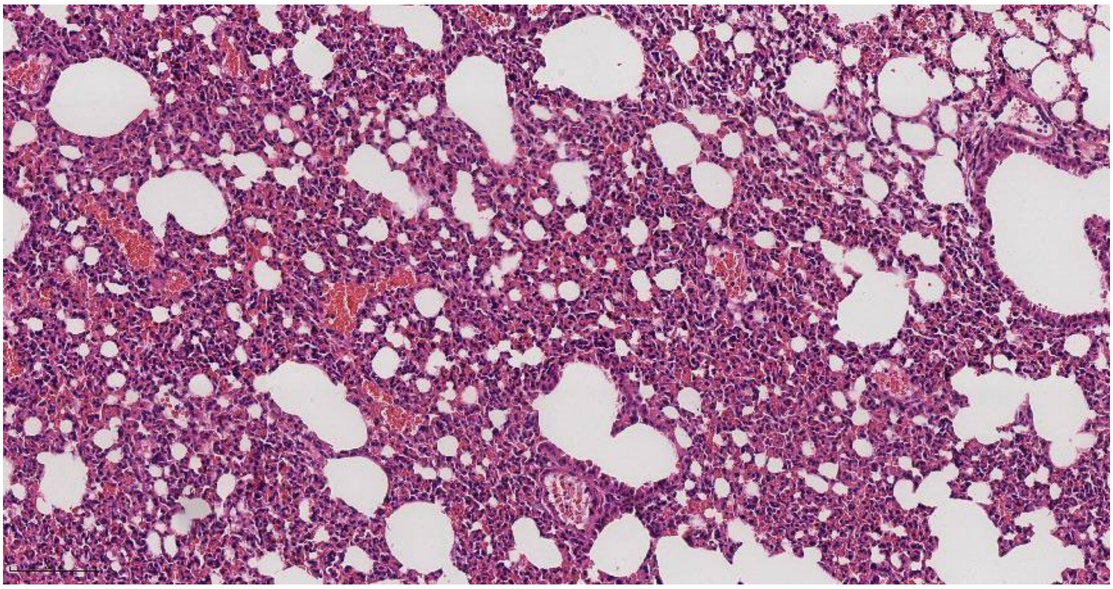

(d)

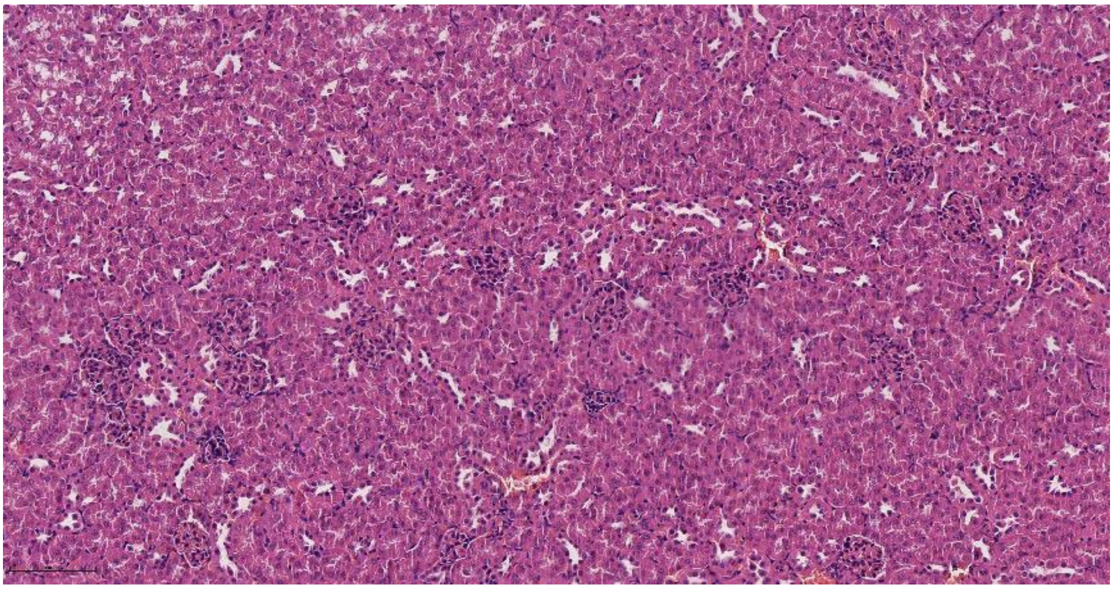

(e)

Figure S21. Analysis of tissue H&E in Saline group. (a)Heart. (b)Liver. (c)Spleen. (d)Lung. (e)Kidney.

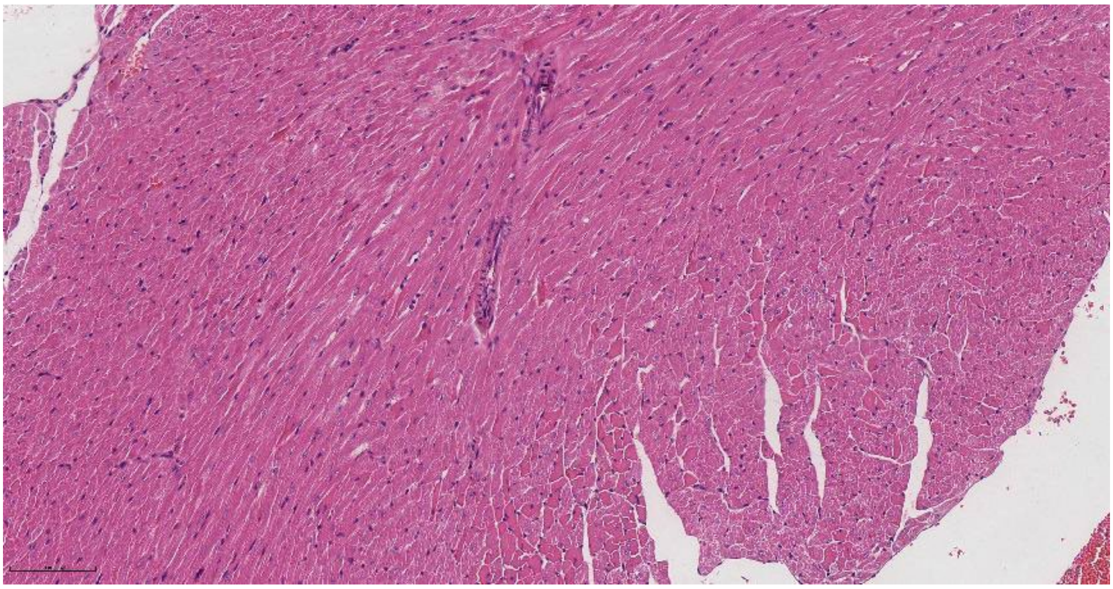

(a)

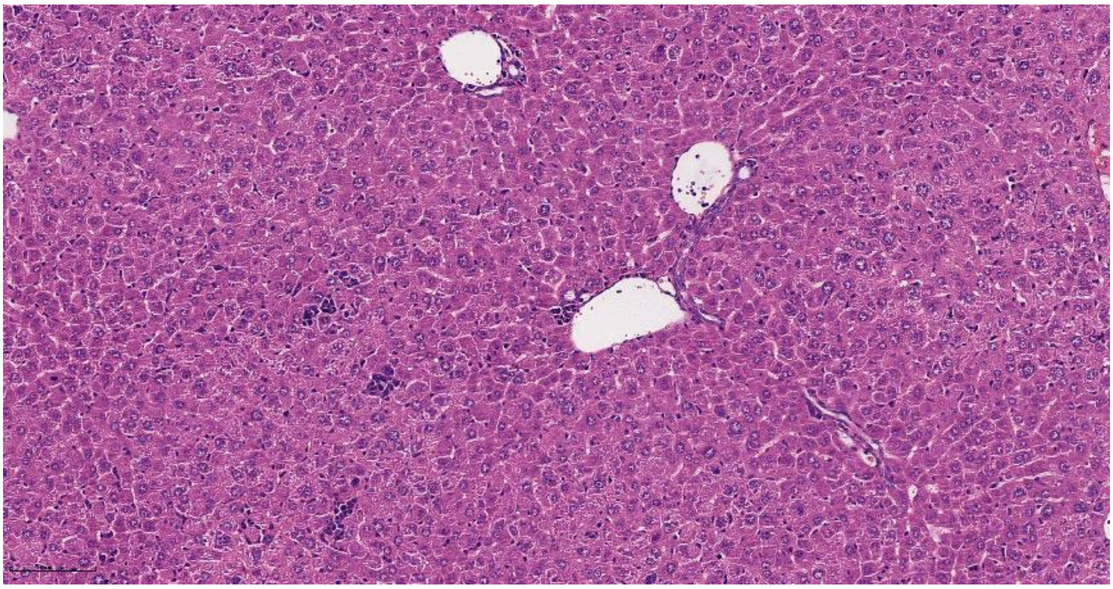

(b)

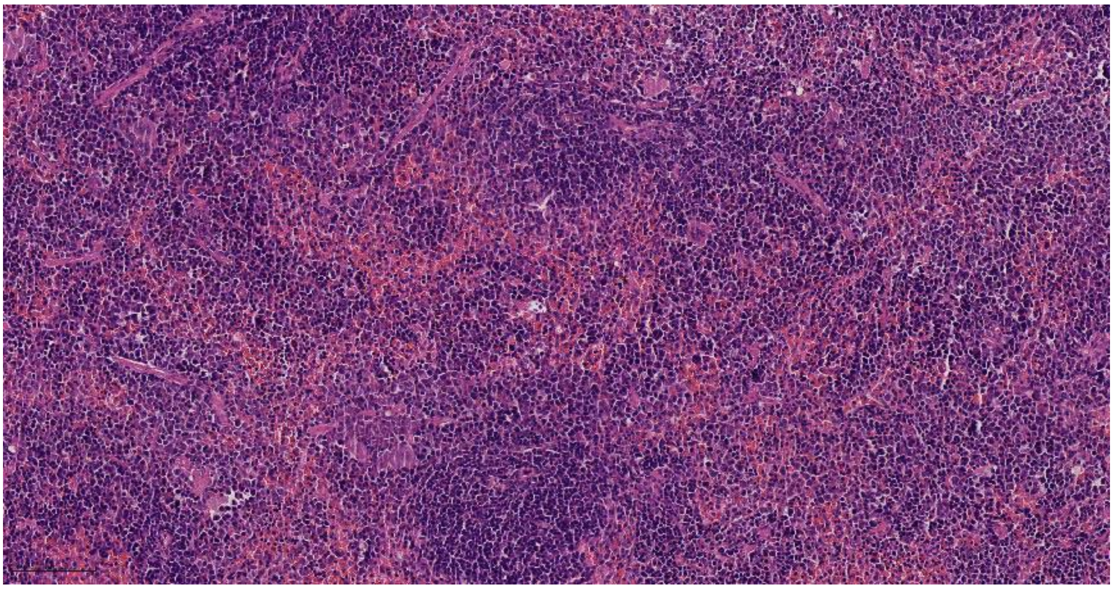

(c)

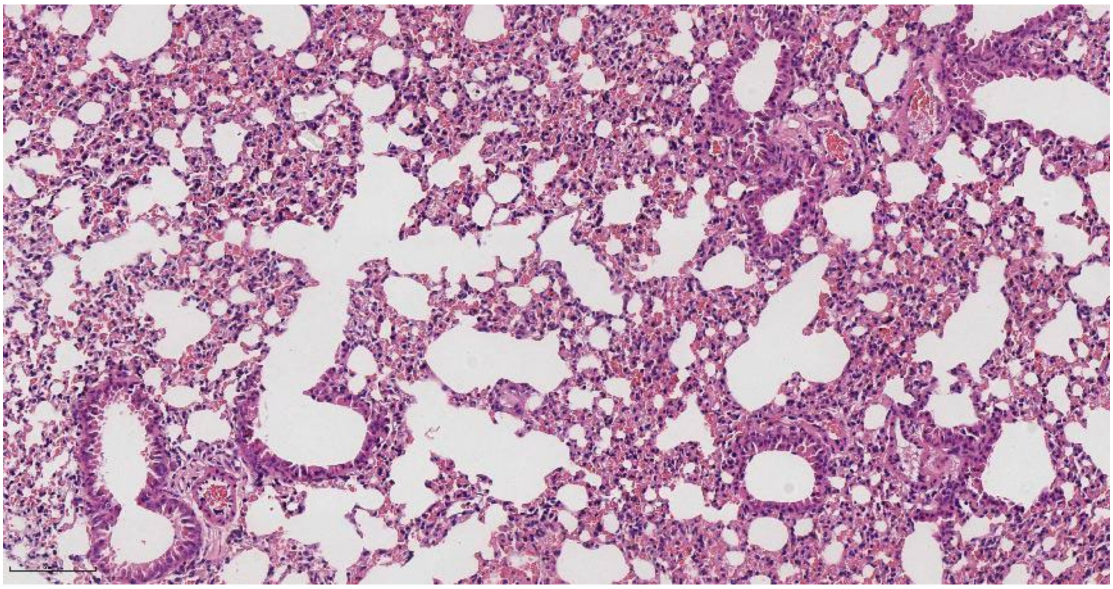

(d)

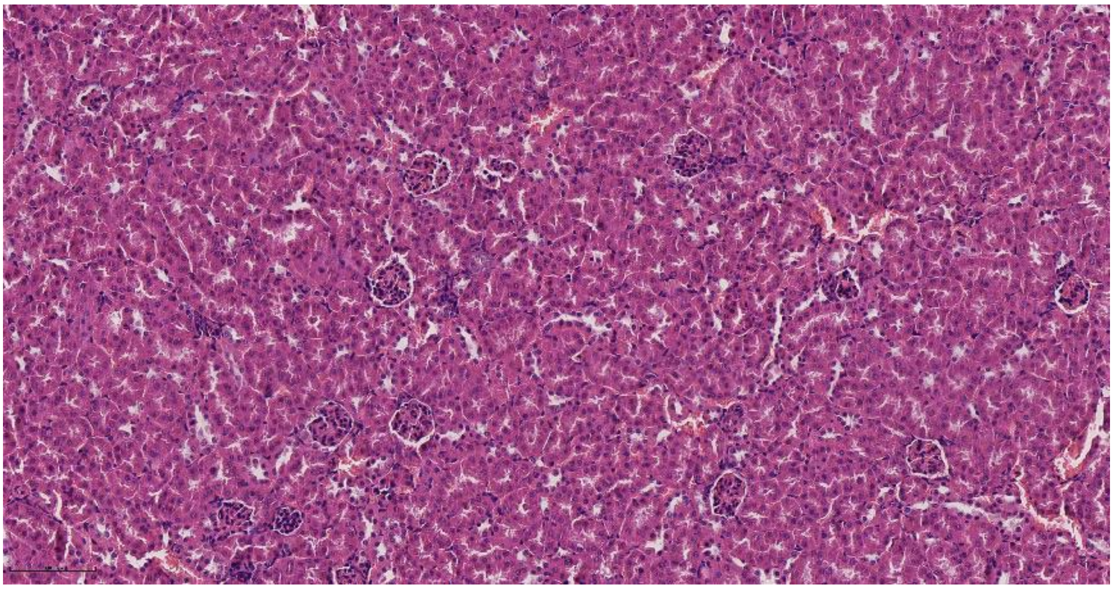

(e)

Figure S22. Analysis of tissue H&E in PTX solution group. (a)Heart. (b)Liver. (c)Spleen. (d)Lung. (e)Kidney.

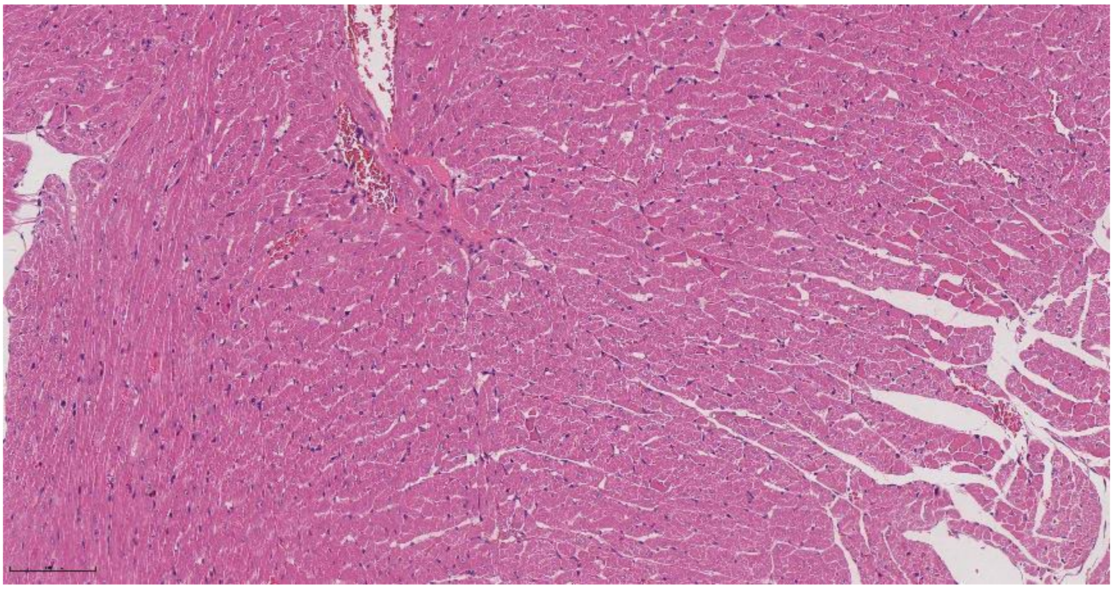

(a)

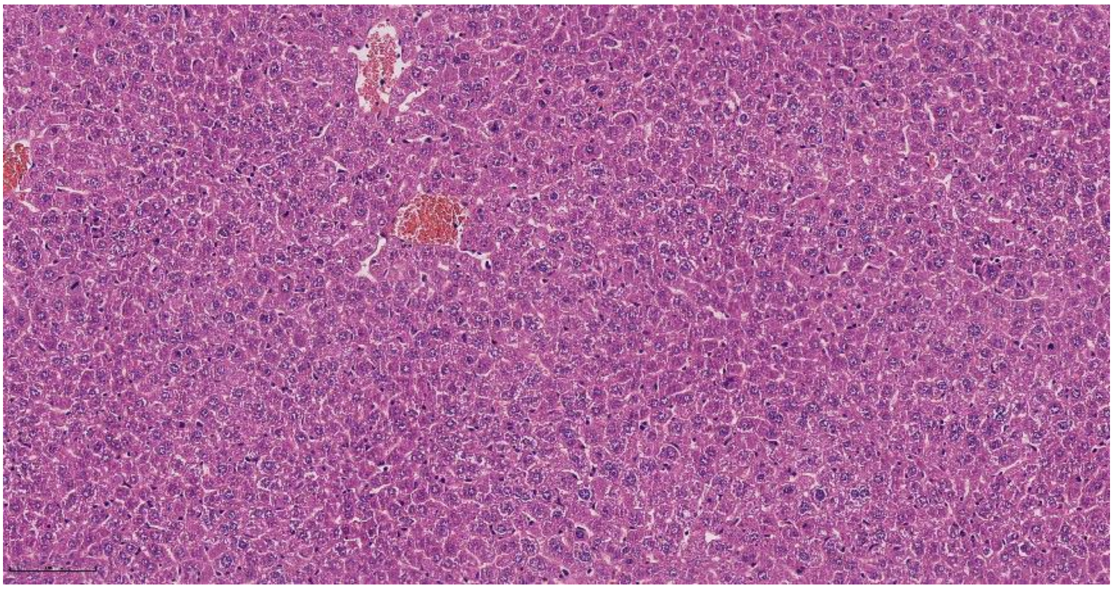

(b)

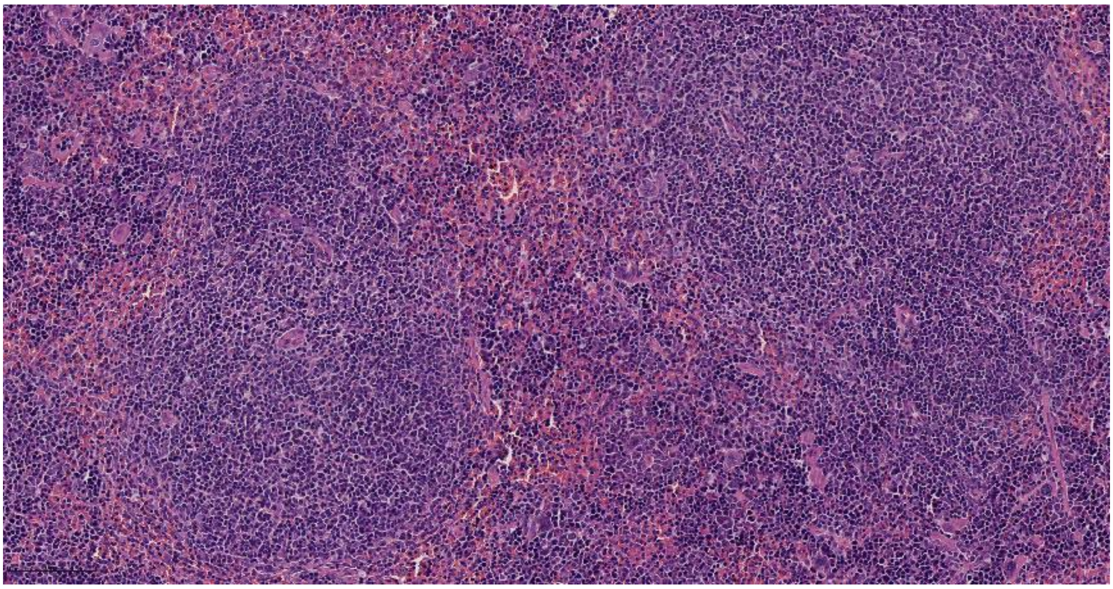

(c)

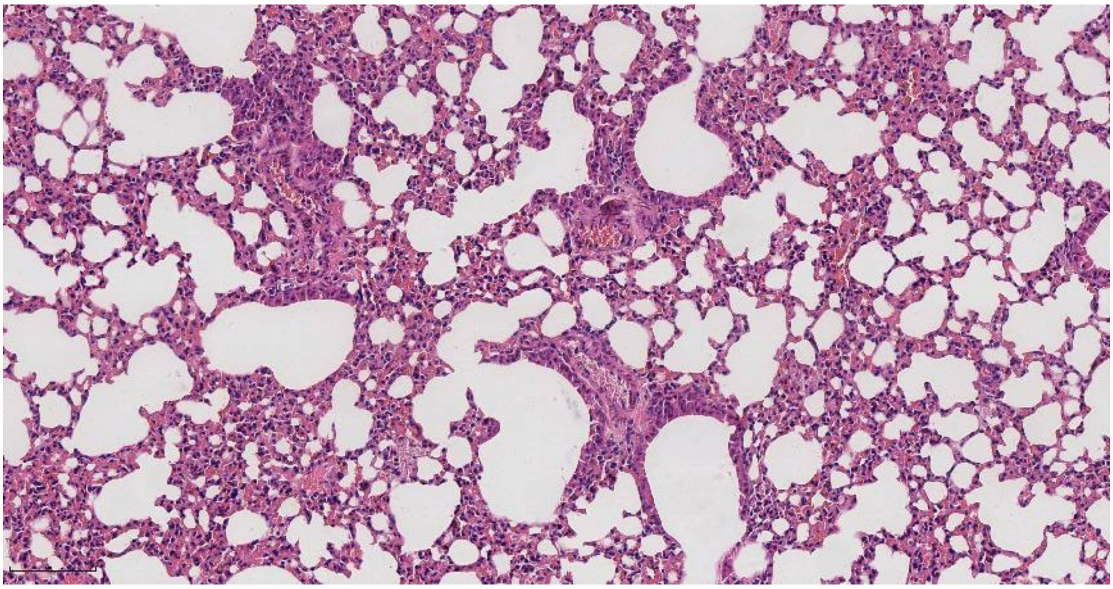

(d)

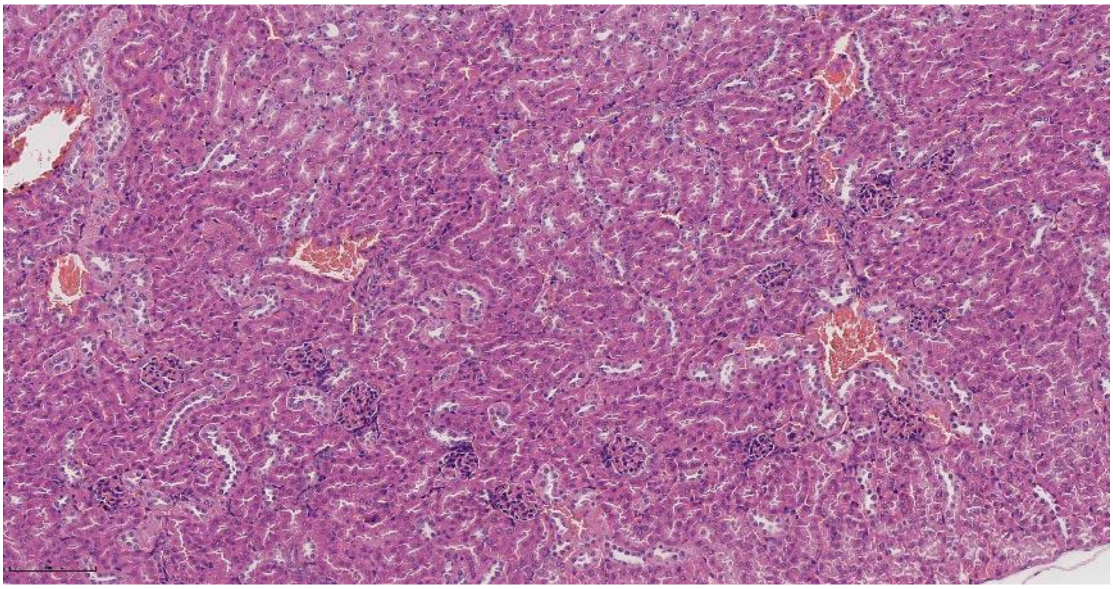

(e)

Figure S23. Analysis of tissue H&E in CUR solution group. (a)Heart. (b)Liver. (c)Spleen. (d)Lung. (e)Kidney.

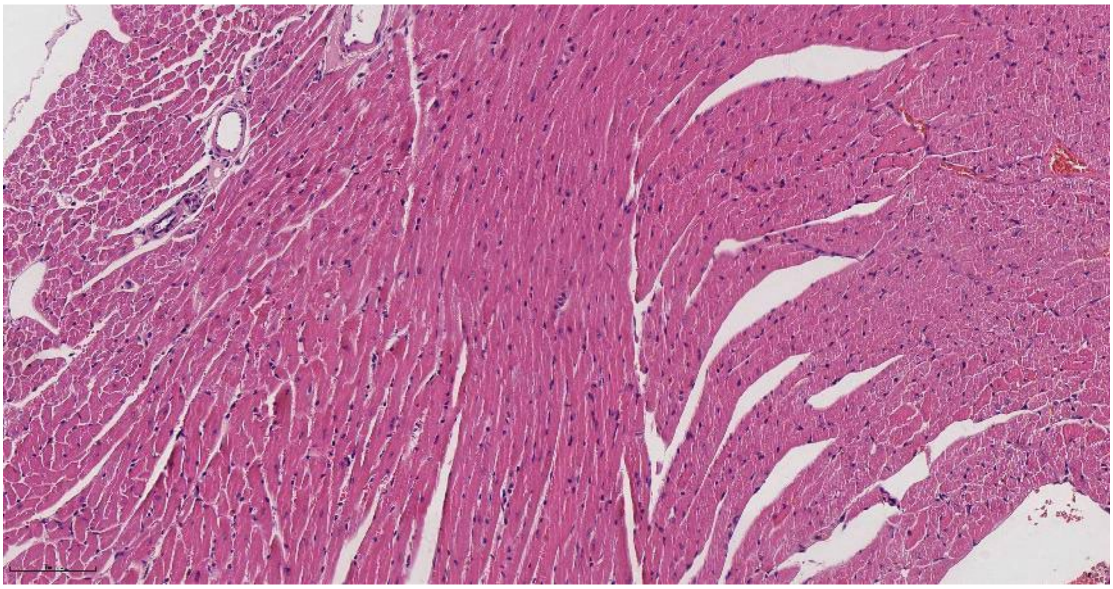

(a)

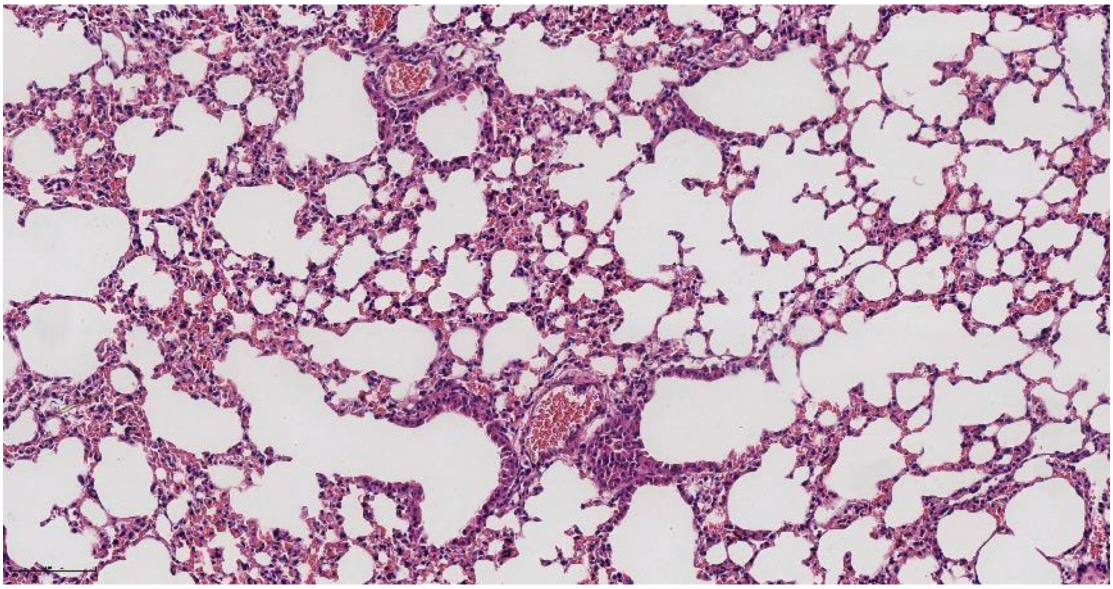

(b)

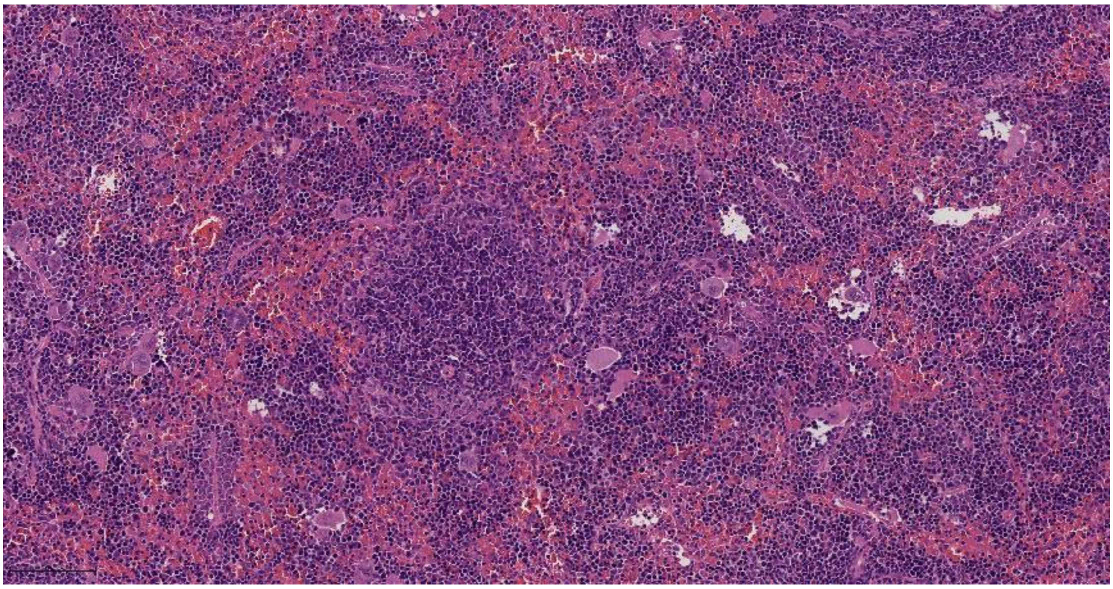

(c)

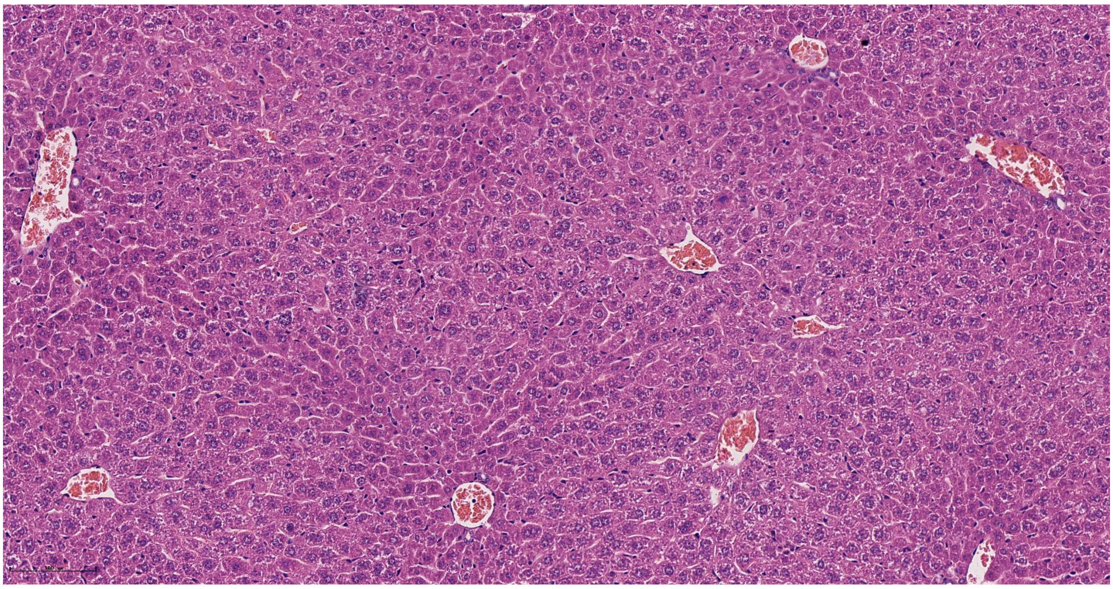

(d)

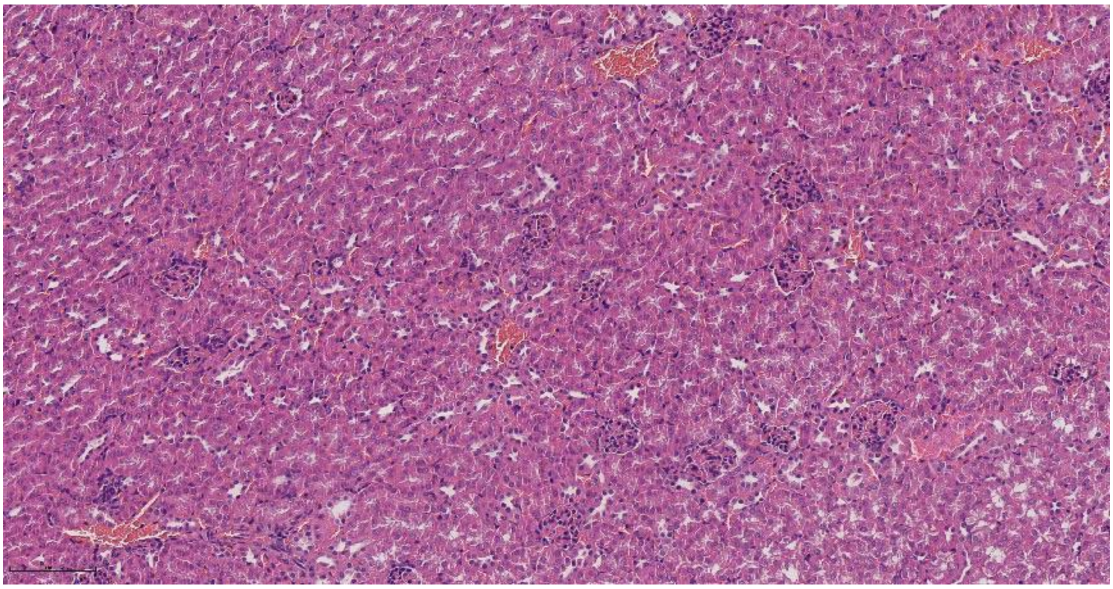

(e)

Figure S24. Analysis of tissue H&E in GA - GL micelles group. (a)Heart. (b)Liver. (c)Spleen. (d)Lung. (e)Kidney.

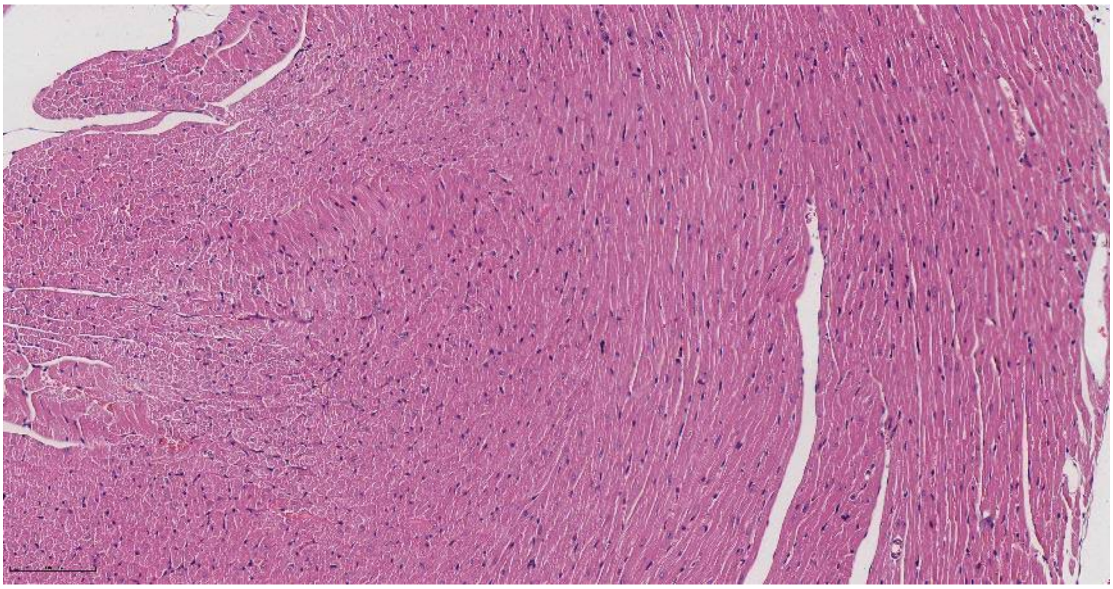

(a)

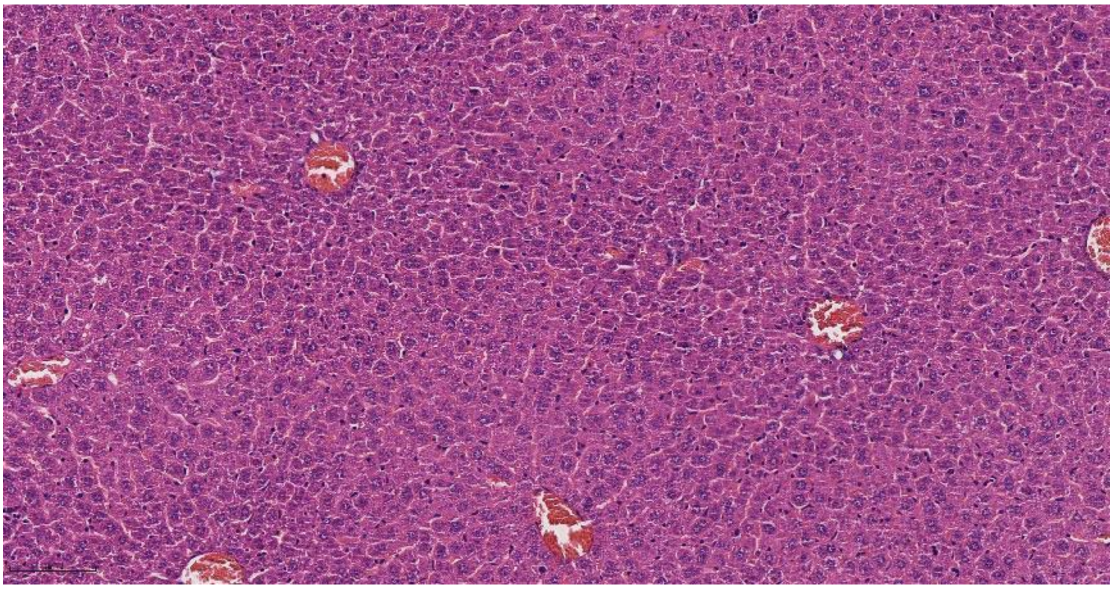

(b)

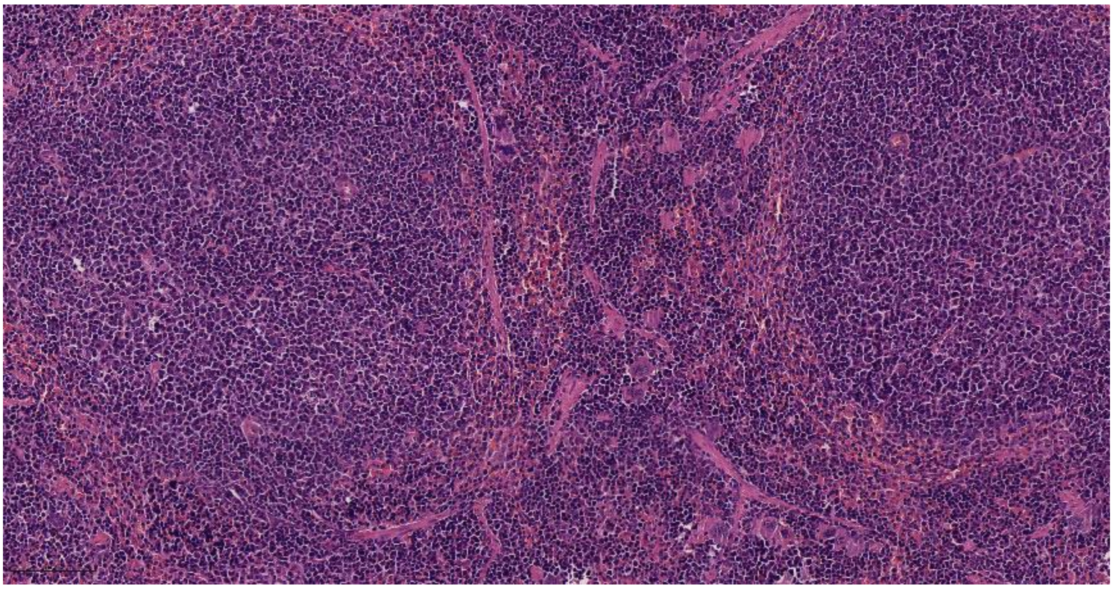

(c)

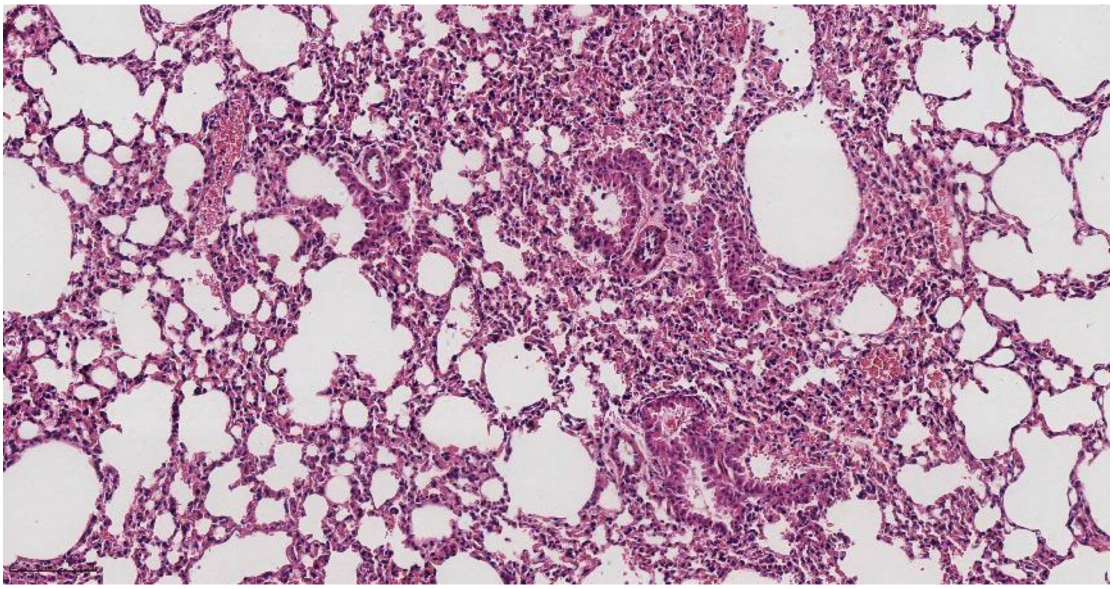

(d)

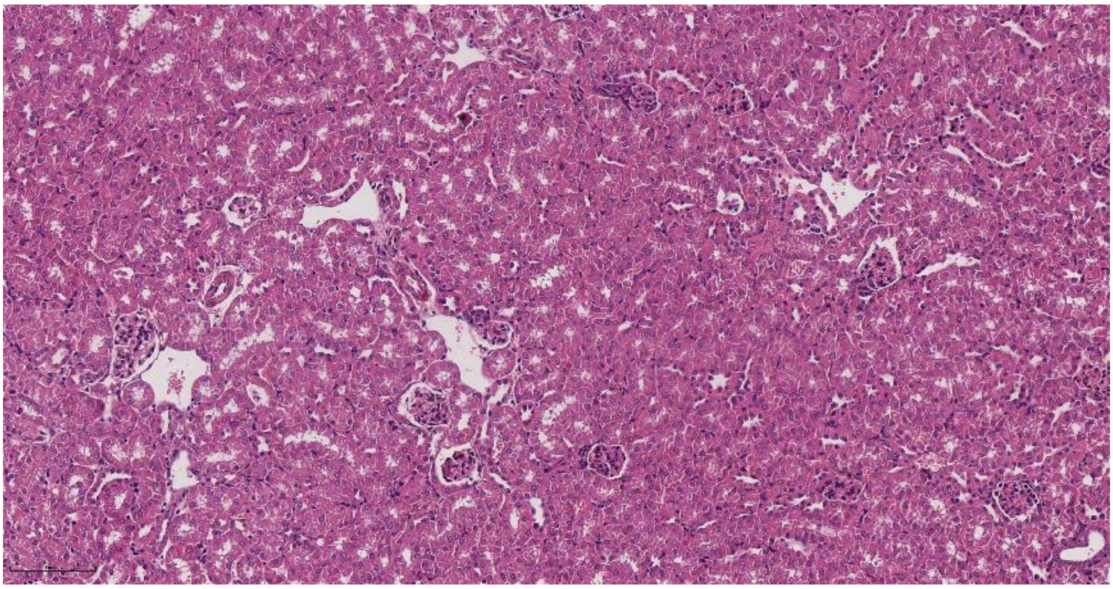

(e)

Figure S25. Analysis of tissue H&E in CUR/GA - GL micelles group. (a)Heart. (b)Liver. (c)Spleen. (d)Lung. (e)Kidney.

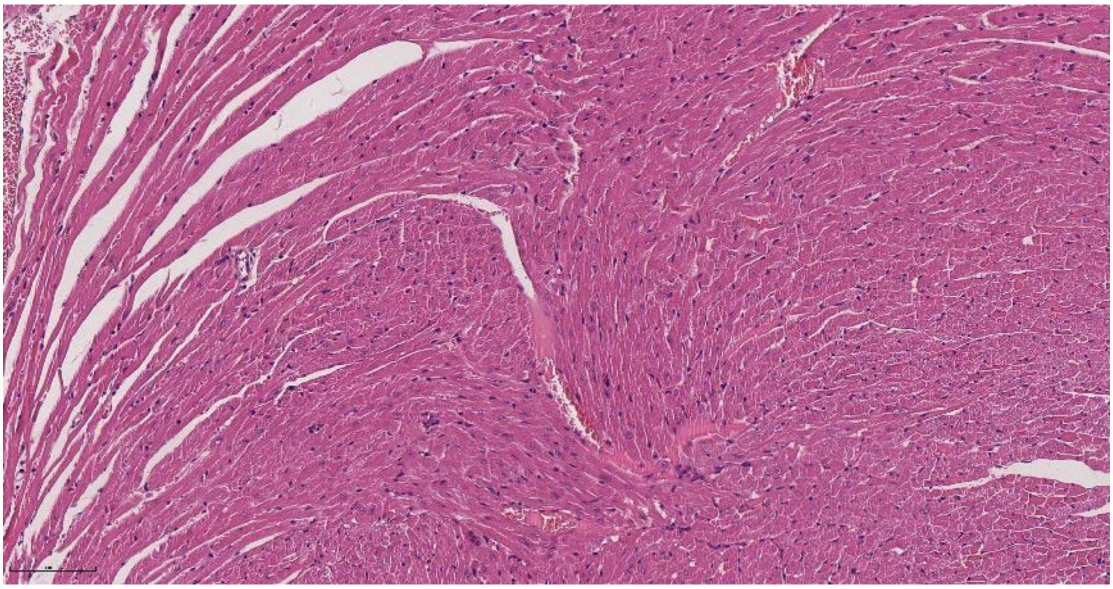

(a)

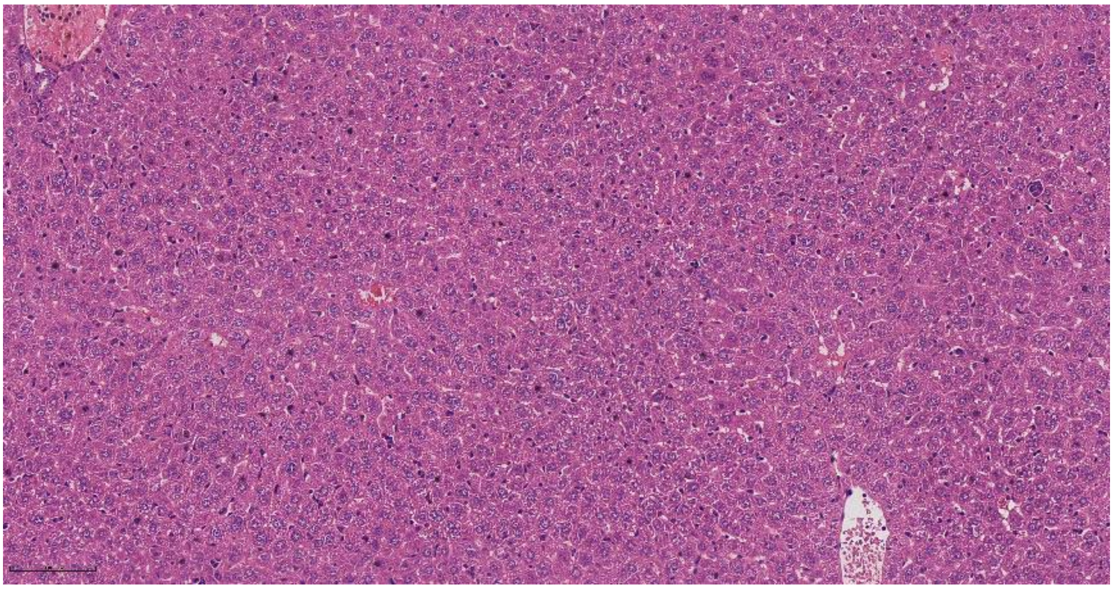

(b)

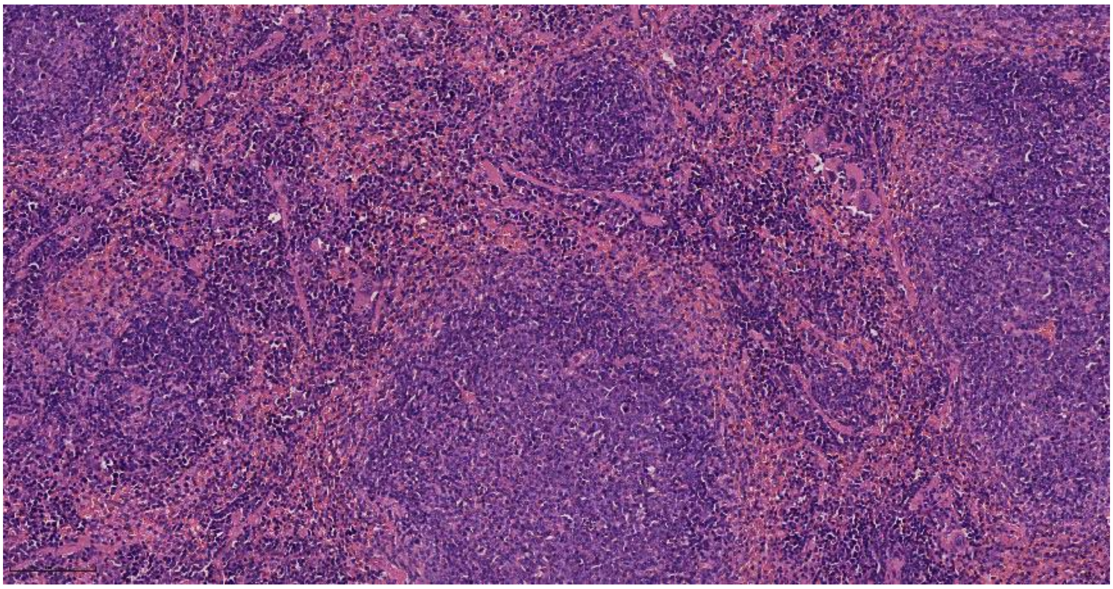

(c)

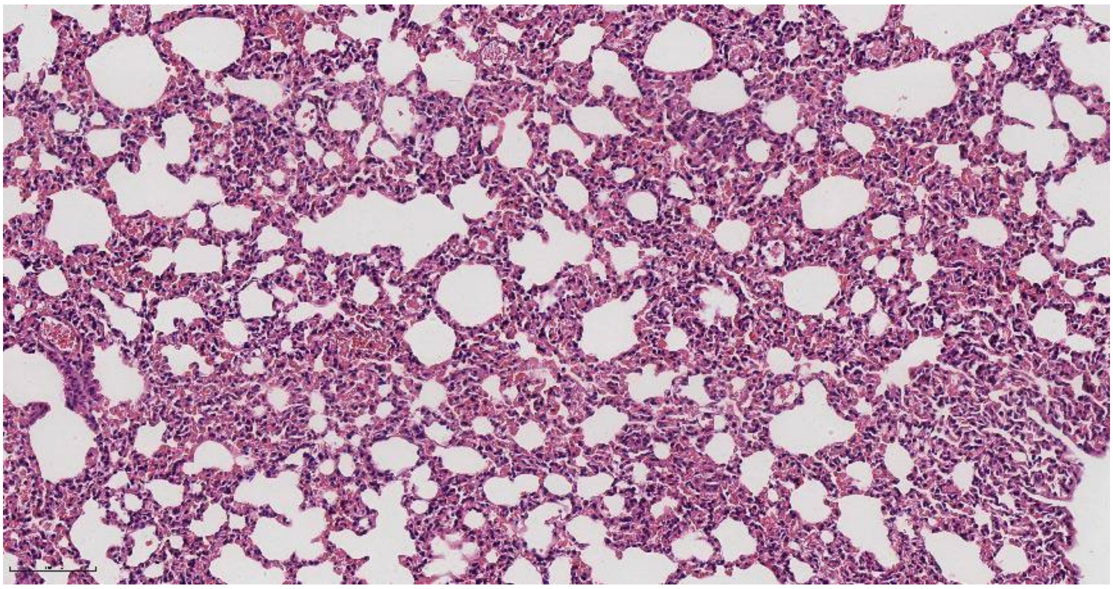

(d)

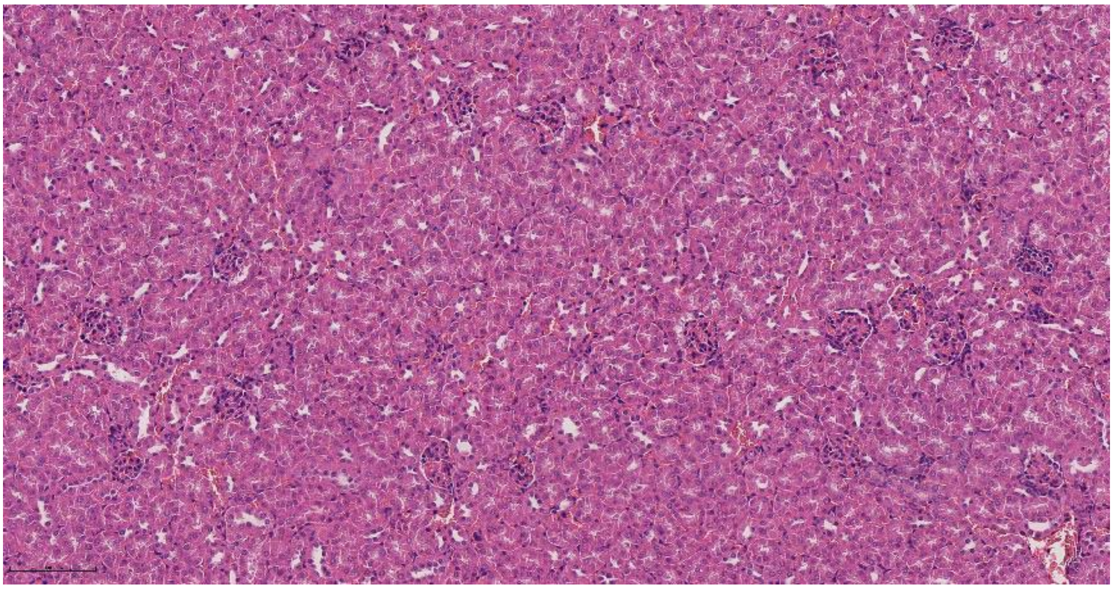

(e)

Figure S26. Analysis of tissue H&E in CGA - GL micelles group. (a)Heart. (b)Liver. (c)Spleen. (d)Lung. (e)Kidney.
